# A Macrophage Differentiation-Mediated Gene: DDX20 as a Molecular Biomarker Encompassing the Tumor Microenvironment, Disease Staging, and Prognoses in Hepatocellular Carcinoma

**DOI:** 10.1155/2022/9971776

**Published:** 2022-10-05

**Authors:** Yi Yang, Ming Yang, Huasheng Pang, Yiwen Qiu, Ting Sun, Tao Wang, Shu Shen, Wentao Wang

**Affiliations:** ^1^Department of Liver Surgery, West China Hospital of Sichuan University, Chengdu, China; ^2^Tibet Center of Disease Control and Prevention, Tibet Autonomous Region, Lhasa 850000, China

## Abstract

**Background:**

DDX20 involves the mechanism of cell proliferate, mitogenic Ets transcriptional suppressor (METS), which can arrest the cell cycle of macrophages. However, little is known about DDX20 expression, clinical values, and the relationship with tumor microenvironment in HCC.

**Methods:**

We mined the transcriptional, protein expression and survival data of DDX20 in HCC from online databases. The immunological effects of DDX20 were estimated by bioinformatic algorithms. The RNAi and CRISPR screening were used to assess the gene effect of DDX20 for the EGFR gene in liver tumor cell.

**Results:**

We found that the DDX20 was highly expressed in HCC. The qRT-PCR result shows a significantly upregulated DDX20 expression in HCC samples from the West China Hospital. The high mRNA expression of DDX20 is associated with a poor survival. DDX20 expression is positively correlated with MDSCs in HCC tissues. Moreover, DDX20 has a high predicted ability for the response to immunotherapy. Furthermore, hsa-mir-324-5p could regulate the macrophage differentiation by interacting with DDX20. Meanwhile, the EGFR gene gets a high dependency score for DDX20.

**Conclusion:**

In sum, DDX20 may serve as a prognostic marker for worse clinical outcomes with HCC and potentially enable more precise and personalized immunotherapeutic strategies in the future.

## 1. Introduction

Hepatocellular carcinoma (HCC) is the sixth most frequently diagnosed cancer, with 830,180 deaths worldwide, according to GLOBOCAN 2020 [1]. Despite the advances in clarifying the etiology and molecular basis of HCC, as well as improvements in treatment strategies, the prognosis of patients remains poor [2]. Liver cancer is a multifactorial disease associated with genetic and epigenetic aberrations of the genome [3]. With the progressively advanced biomedical and clinical research, we have witnessed a highlighted role of abnormal molecular and signaling pathway mechanisms in underlying the progression of liver cancer. Presently, two biotherapies, molecular targets, and immunotherapy are available for clinical therapy [4, 5]. However, the heterogeneous nature (intertumoral and intratumoral) of the tumor is a significant feature of HCC and renders the subset of patients that seems to benefit little from those therapies. Thus, defining novel therapeutic target genes and/or predictive markers for liver cancer are urgently needed to decipher the biological complexity of this disease and improve clinical outcomes.

DEAD-box RNA helicase 20 (DDX20) first acts as an ATP-dependent RNA helicase which is involved in more than one cellular process. In gastric cancer, DDX20 promoted MGC-803 and AGS cell growth, migration, and invasion in vitro functional experiments [6]. There are reports on the regulatory function of DDX20 for the transcription of targeted genes and splicing through binding some transcription factors or interacting with the survival of motor neurons (SMN) protein [7, 8]. Tumorigenesis is a complex, multifactorial process involving changes in genetic and environmental factors, among which posttranscriptional regulatory plays an essential regulatory role [9]. Given the close relationship between DDX20 protein and the mRNA stability in cells, we premise that abnormal DDX20 expression may be critical in the pathogenesis of HCC. However, no studies have yet characterized the tumor biology of DDX20 in liver cancer.

Here, we systematically analyzed the relationships between the DDX20 expression level and HCC patients' clinical outcomes, tumor multiomics, and microenvironment using publicly available transcriptome data. We were able to demonstrate that DDX20 is an important cancer-promoting molecule in liver tumors and exhibits a therapeutic target and tumor immune-suppressive features.

## 2. Material and Methods

### 2.1. Data Processing and Analysis of DDX20 Expression

The DDX20 transcriptional and protein data in human normal tissues and cancer cell lines are included in proteomicsDB database (http://www.proteomicsdb.org/). HCC patients' clinical information and RNA-seq data were obtained from The Cancer Genome Atlas (TCGA, https://portal.gdc.cancer.gov/). DifferentialDDX20 expression analysis among cirrhotic liver tissues, liver cancerous tissues, and noncancerous tissue was performed at TCGA cohort and HCCDB cohort (http://lifeome.net/database/hccdb). The matched noncancerous tissue was obtained from Genotype-Tissue Expression (GTEx, http://gtexportal.org) projects. The correlation between DDX20 expression and HCC patients' clinical characteristics, including cancer T stage, pathologic stage, weight, height, race, and albumin, were analyzed based on the HCC-TCGA cohort. UALCAN (http://ualcan.path.uab.edu/home) was used to examine the DDX20 DNA methylation status in liver cancer and normal samples and investigate the relationship between the methylation of DDX20 gene promoter and different clinical characteristics.

### 2.2. Survival Analysis of DDX20 in Liver Cancer

The Kaplan-Meier (KM) method was used to explore the prognostic value of DDX20, and the clinical outcome mainly consisted of overall survival (OS), progression-free interval (PFI), and disease-free survival (DSS).

### 2.3. Tumor Immunology Analysis of DDX20 in Liver Cancer

We first used the Single-Sample GSEA (ssGSEA) tool [10] to quantify the enrichment levels of 24 immune cells and then analyzed the correlation of the immune cells scores with DDX20 expression. A microenvironment comprehensive score [11] was calculated to investigate the effect of DDX20 on the tumor microenvironment (TME). Next, the coexpression analysis of classical immune checkpoint molecules with DDX20 was performed. Because myeloid-derived suppressor cells (MDSCs) induce detrimental immunosuppression [12], we conducted a correlation analysis of DDX20 and MDSC and evaluated the survival impact between DDX20 and MDSCs for HCC at the Tumor IMmune Estimation Resource (TIMER) online database (http://cistrome.shinyapps.io/timer). Furthermore, we ran the biomarker relevance of DDX20 compared to standardized cancer immune evasion biomarkers in multiple cancer cohorts treated with immune checkpoint blockade (ICB) at TIDE database (tide. dfci. harvard. edu). The relationship between cytotoxic T-cell levels (CTLs), dysfunctional T-cell phenotypes, and DXX20 expression levels was also explored concurrently.

### 2.4. Genome-Scale shRNA and CRISPR Screening Data Analysis of DDX20 in Liver Tumor Cell

To detect the gene effect of DDX20 for liver tumor cells, in vivo shRNA and CRISPR screening was performed using a previously published shRNA library. The gene effect analysis was achieved at Dependency Map (DepMap) portal (https://depmap.org/portal/). The Cancer Gene and Pathway Explorer (CGPE) provides gene-level dependency scores across hundreds of cell lines (https://depmap.org/portal/).

### 2.5. miRNA-mRNA Network Analysis of DDX20 in Liver Cancer

Using the PathCards tool (http://pathcards.genecards.org), we found that DDX20 involves a mechanism of cell proliferate mechanism, mitogenic Ets transcriptional suppressor (METS), which regulated the cell cycle of macrophages. For finding key mRNA, the top 300 mRNAs positively related with DDX20 based on HCC RNA-seq data were selected to take the intersection of core genes from mitogenic Ets transcriptional suppressor (METS). Finally, DDX20 and coexpression genes were input NetworkAnalyst software (https://www.networkanalyst.ca/) to identify a miRNA-mRNA network.

### 2.6. Functional Enrichment Analysis of DDX20 in Liver Cancer

We next determined the functional annotation of DDX20 in HCC. Gene Ontology (GO) and Kyoto Encyclopedia of Genes and Genomes (KEGG) pathway enrichment analysis were performed for the selected genes (the differential expression genes from HCC-TCGA which are grouped by the expression of DDX20). To further verify the enrichment analysis of the KEGG pathway, gene set enrichment analysis was further conducted. The gene sets were downloaded from the GSEA database (https://www.gsea-msigdb.org/) including Curated gene sets, Computational gene sets, Ontology gene sets, Oncogenic signature gene sets, Immunologic signature gene sets, and Hallmarker gene sets.

### 2.7. Liver Tissue Collection and qRT-PCR

Thirty pairs of hepatocellular carcinoma tissue and adjacent normal liver tissue were obtained from patients undergoing liver resection at the West China Hospital. These patients are all infected with hepatitis B virus (HBV) and have not been vaccinated against HBV. The protocol of this study was approved by the Ethics Committee of West China Hospital, Sichuan University. After quality testing, the RNAs were reversely transcribed into cDNAs. Real-time quantitative fluorescence PCR (qRT-PCR) assay was used to detect the expression levels of DDX20. The following primer sequences for this assay were used: DDX20 (forward): 5′-CTTCGAGTCACTGCTGCTTTC-3′ and (reverse): 5′-GTGCCAGATTTAGCTTGAACAA-3′; ACTB (forward): 5′-CGATCCGCCGCCCGTCCACA-3′ and (reverse): 5′-ACGCAGCTCATTGTAGAAGGGTGGTG-3′.

### 2.8. Statistical Analysis

The R language (R version 4.1.0) was used for data processing and graphics' drawing. Briefly, a comparison of mRNA expression in normal tissue and cancer tissue used Student's *t*-test. The statistical method that was used in the Kaplan-Meier curve analysis was the log-rank test. The relationships between the various variable and DDX20 expression were analyzed using Spearman's or Pearson's test. *P* < 0.05 was considered to indicate a statistically significant difference (^∗^*P* < 0.05,  ^∗∗^*P* < 0.01, and^∗∗∗^*P* < 0.001).

## 3. Results

### 3.1. DDX20 Expression Profiles in Human Normal Tissues and Cancer Cell Lines

**Figure 1:**
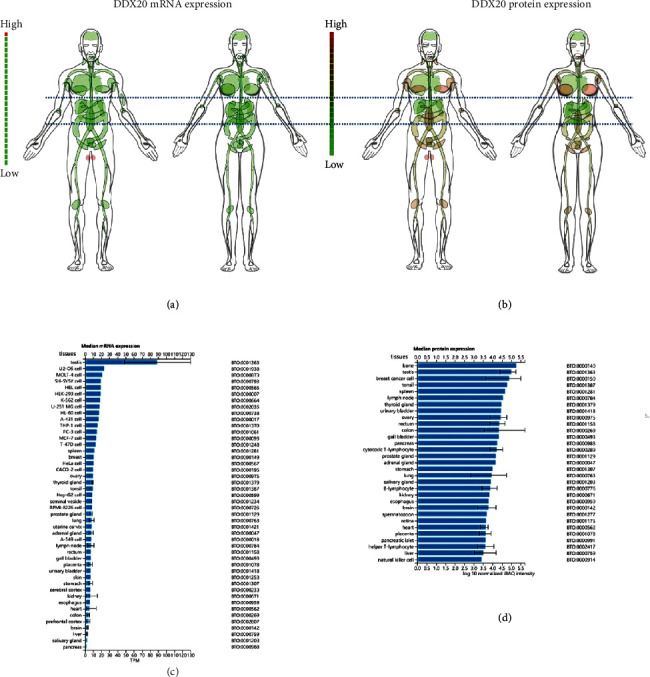
DDX20 expression profiles. (a, b) DDX20 expression profiles in normal human tissues and cancer cell lines. (c, d) The protein expression profiles of DDX20 in human normal tissues. Data was obtained via proteomicsDB.

Analysis of data from the proteomicsDB databases revealed that the DDX20 gene has tissue-specific mRNA and protein expression in different organs of humans. Comparing other tissues of humans, the DDX20 mRNA expression and protein are at very low in normal liver. Note here that the translational level of DDX20 is also markedly different in various cancer cell lines (Figures 1(a)–1(d)).

**Figure 2:**
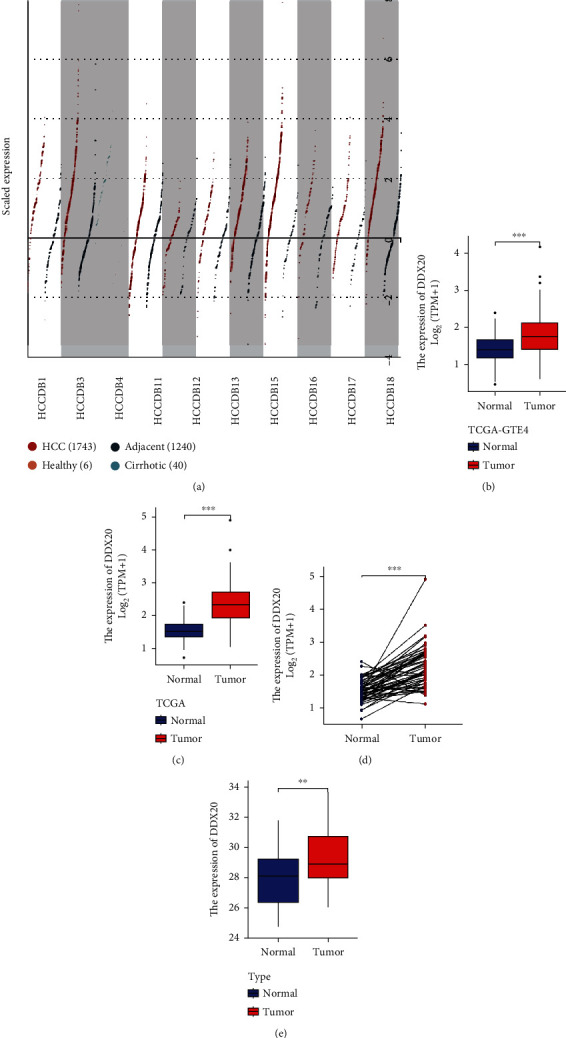
DDX20 expression levels in liver cancer. (a) Increased DDX20 in different datasets of liver cancer compared with normal tissues and cirrhotic in the HCCDB database. (b) Comparisons of CD96 expression levels between tumor tissues from TCGA database and normal tissues from the GTEx database. (c) Differential DDX20 expression in tumor tissue and matching normal tissue from TCGA database. (d) DDX20 expression in indicated paired tumor and normal tissues in LIHC data of TCGA. (e) qRT-PCR result showed the expression of DDX20 in liver cancer tissues. Data was obtained via West China Hospital. ^∗^*P* < 0.05,  ^∗∗^*P* < 0.01, and^∗∗∗^*P* < 0.001.

Next, we explored the transcriptional level of DDX20 in LIHC, finding a series of differences that may be associated with primary lesions. As shown in Figure 2(a), the DDX20 gene is highly upregulated in tumor tissues of nine HCC cohorts compering with adjacent, cirrhotic, and healthy liver tissues. We found a marked elevation in DDX20 expression in LIHC-TCGA samples (either integrate with GTEz data or not, both elevated) compared to normal liver tissues (Figures 2(b) and 2(c)). The paired analysis result was also consistent with the above finding (Figure 2(d)). To verify these results, we further performed quantitative reverse transcription PCR on our liver cancer patient samples. The DDX20 expression pattern was overexpressed in the liver tumor tissues (Figure 2(e)).

### 3.2. Association of DDX20 Expression with Clinical Parameters and Influence on Liver Cancer Patient Survival

**Figure 3:**
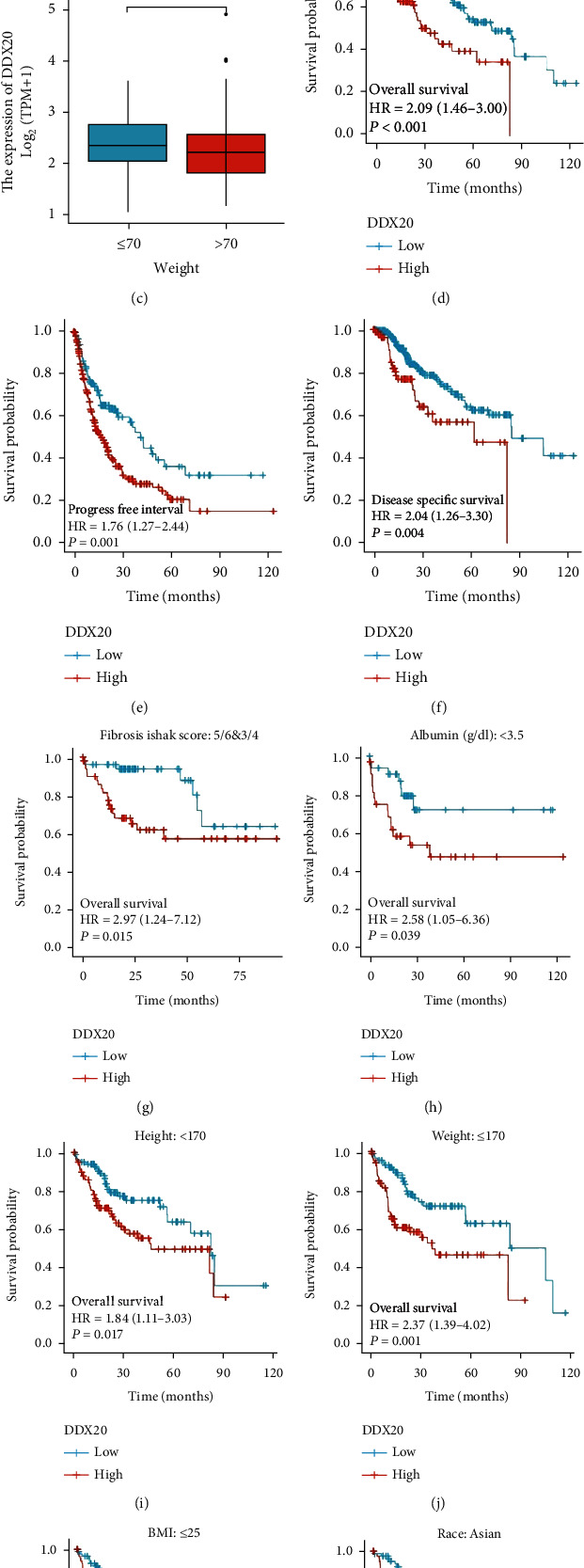
The association of DDX20 expression with clincopathological factors. (a) Expression of DDX20 in LIHC with different T stages. (b) Association between DDX20 expression and pathological stage. (c) The relative expression levels of DDX20 in weight > 70 or <70 patients with LIHC. (d–f) Prognostic value of DDX20 in all patients with HCC based on OS, PFI, and DSS. (g–l) Survival curves of OS with significance in fibrosis Ishak score, albumin, height, weight, BMI, and race subgroups between liver cancer cohorts with high and those with low expression levels of DDX20. Data was obtained via TCGA.

Results of the differential expression analysis of the DDX20 gene indicated it is likely playing an oncogene role in the liver tumors. Thus, we employed the RNA-seq data of TCGA-LIHC to determine the correlations between DDX20 and clinical indices. In Figures 3(a) and 3(b), patients with the T3 stage or AJCC stage had higher DDX20 transcriptional levels than patients who were T1 stage or AJCC stage I, respectively (*P* < 0.05). In Figure 3(c), lower expression of DDX20 is associated with a heavier weight of patients.

Next, to analyze the prognostic impact of DDX20 on OS, PFI, and DSS, we used KM curve analysis. LIHC patients with high DDX20 expression in these analyses had a worse prognosis than those with low DDX20 expression, including OS (HR = 2.09, 95% CI: 1.46-3.00, *P* < 0.001), PFI (HR = 1.76, 95% CI: 1.27-2.44, *P* = 0.001), and DSS (HR = 2.04, 95% CI: 1.26-3.30, *P* = 0.004) (Figures 3(d)–3(f)). The results for these prognostic models can be found in the additional file (supplementary file 1).

In addition, we investigated the association between the DDX20 expression level and OS of a liver cancer patients in six subgroups. The result showed a significant reduction in the survival time of patients with DDX20 overexpressed in six subgroups including those patients with fibrosis Ishak score: 3-6, albumin < 3.5, height < 170 cm, weight ≤ 70 kg, and BMI ≤ 25 (Figures 3(g)–3(l)).

### 3.3. DDX20 DNA Methylation Status in Liver Cancer

**Figure 4:**
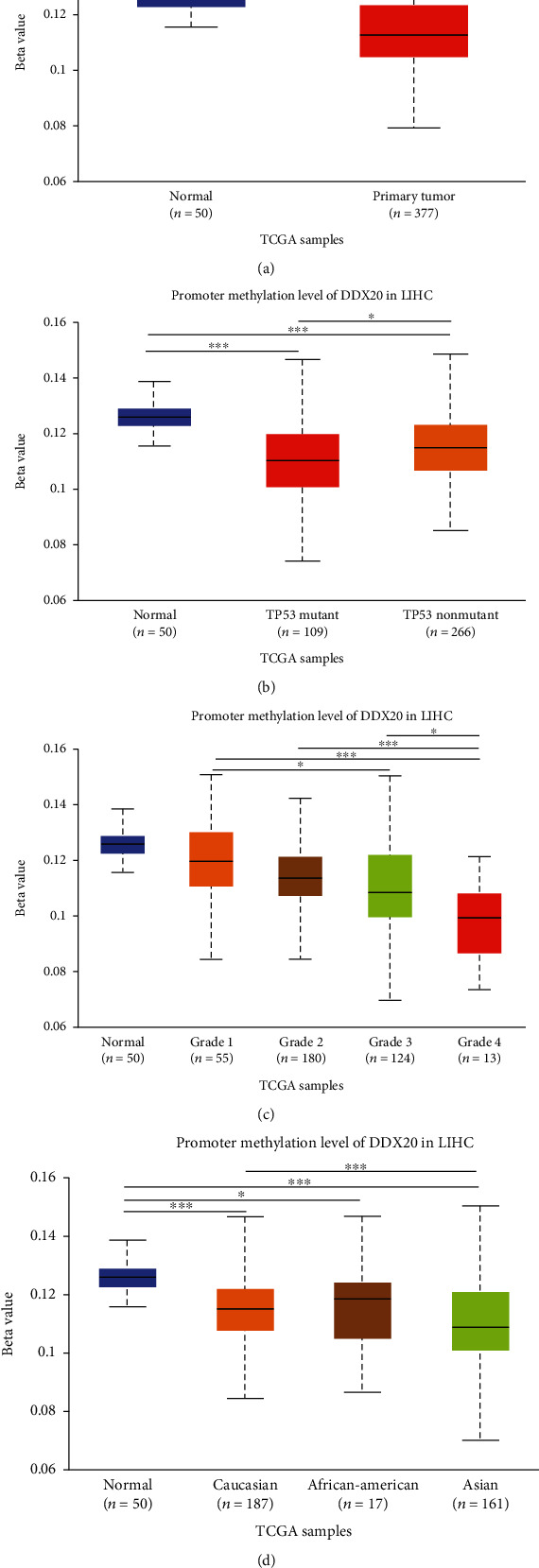
Analysis of the relationship between DDX20 expression and promoter methylation level of DDX20. (a) Differential analysis of between LIHC and Control. (b–d) Correlation between promoter methylation level of DDX20 between TP53 statue, tumor grade, and patient's race. ^∗^*P* < 0.05,  ^∗∗^*P* < 0.01, and^∗∗∗^*P* < 0.001. Data was obtained via UALCAN.

UALCAN analysis of DNA methylation provided us a piece of important information regarding the DDX20 methylation level of liver cancer patients with different clinical features. Compared to normal groups, promoter hypomethylation of DDX20 gene occurred in the primary tumor group (Figure 4(a)) Compared with LICH patients without TP53 mutations (*n* = 266), a significantly low promoter methylated of DDX20 in patients with mutations in TP53 (*n* = 109) was found (Figure 4(b)) In addition, promoter hypomethylation of DDX20 in liver cancer patients was significantly decreased with tumor pathological grade (Figure 4(c)). Besides, Asian patients with liver tumor had a lower level of promoter methylation of DDX20 than that Caucasian patients with liver tumor (Figure 4(d)). In summary, DNA gene promoter methylation might contribute to the abnormal upregulation of DDX20 in liver cancer.

### 3.4. Immune Correlates of DDX20 Expression in Liver Cancer

**Figure 5:**
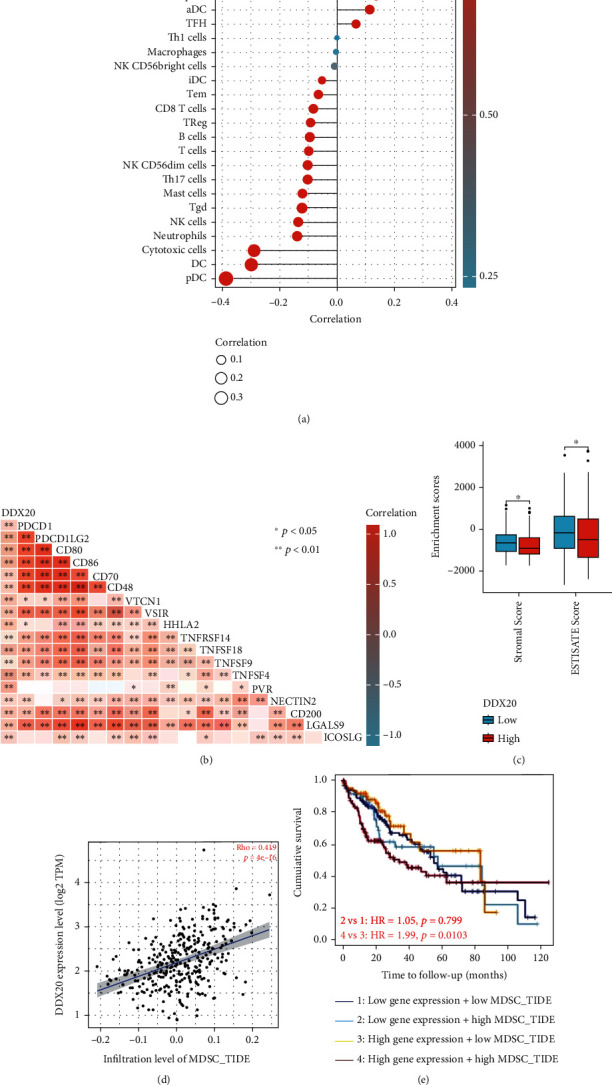
Correlation analysis of DDX20 level and immune microenvironment in LIHC. (a) DDX20 expression in LIHC tissues negative correlates with 9 immune cell types. (b) The correlations between DDX20 and confirmed immune checkpoints in LIHC. (c) The StromalScore and ESTIMATEScore in upregulated DDX20 tumor tissues were significantly lower than those in downregulated DDX20 tumor tissues. (d) Correlation between DDX20 and infiltrated MDSC in LIHC; (e) KM curves according DDX20 expression and MDSC Infiltrating level in LIHC. ^∗^*P* < 0.05,  ^∗∗^*P* < 0.01, and^∗∗∗^*P* < 0.001. Data was obtained via TCGA.

As cancer progresses, the complexity of the network between tumor cells and cells of the tumor microenvironment is gradually increased [13]. Tumor biology and immunology also change over the course of the tumor transformation process and the response to immunotherapy [14]. We, therefore, conducted correlational analyses between the DDX20 expression and immune cell infiltration of LIHC using TCGA data. Notably, the number of T helper cells, Th2 cells, Tcm, Tgd, and macrophages both have a positive relationship with the level of DDX20 mRNA transcription. In contrast, Treg, pDC, and CD56bright cells were negatively correlated with DDX20 expression in liver tumor. Next, we found that multiple classic immune checkpoint expression was directly proportional to DDX20 expression in the liver tumor, such as PDCD1, PDCD1LG2, and VTCN1 (Figures 5(a) and 5(b)).

The StromalScore and ESTIMATEScore represented the immune and tumor purity in TME separately [15]. The above two scores were calculated in this study, and the result showed that liver cancer patients with DDX20 upregulated had lower scores both of two compared with those with DDX20 downregulated (Figure 5(c)).

As a major immune cell that squelches overactive antitumor immune responses in TME, myeloid-derived suppressor cell (MDSC) was closely associated with cancer patients' clinical outcomes [13]. Thus, we used TIDE arithmetic to determine the abundance of MDSC in the liver tumor at the TIMER database (https://cistrome.shinyapps.io/timer/). The results showed that the DDX20 expression was positively related MDSC infiltration. In addition, for patients with overexpression of DDX20, a higher MDSC infiltrated level means a worse survival status (Figures 5(d) and 5(e)).

### 3.5. DDX20 Expression Is Associated with Immune-Oncological Phenotypes

To date, the application of checkpoint inhibitors in cancer therapy achieved a striking improvement in survival [16]. But the niche and heterogeneity of tumor may result in immune checkpoint-targeting drugs being inefficacious [17]. To assess the value of DDX20 gene as an immuno-tumor biomarker, we used the TIDE algorithm, base TIDE framework (tide. dfci. harvard. edu), to quantify its predictive power of treatment response for immune checkpoint inhibitors (ICBs).

**Figure 6:**
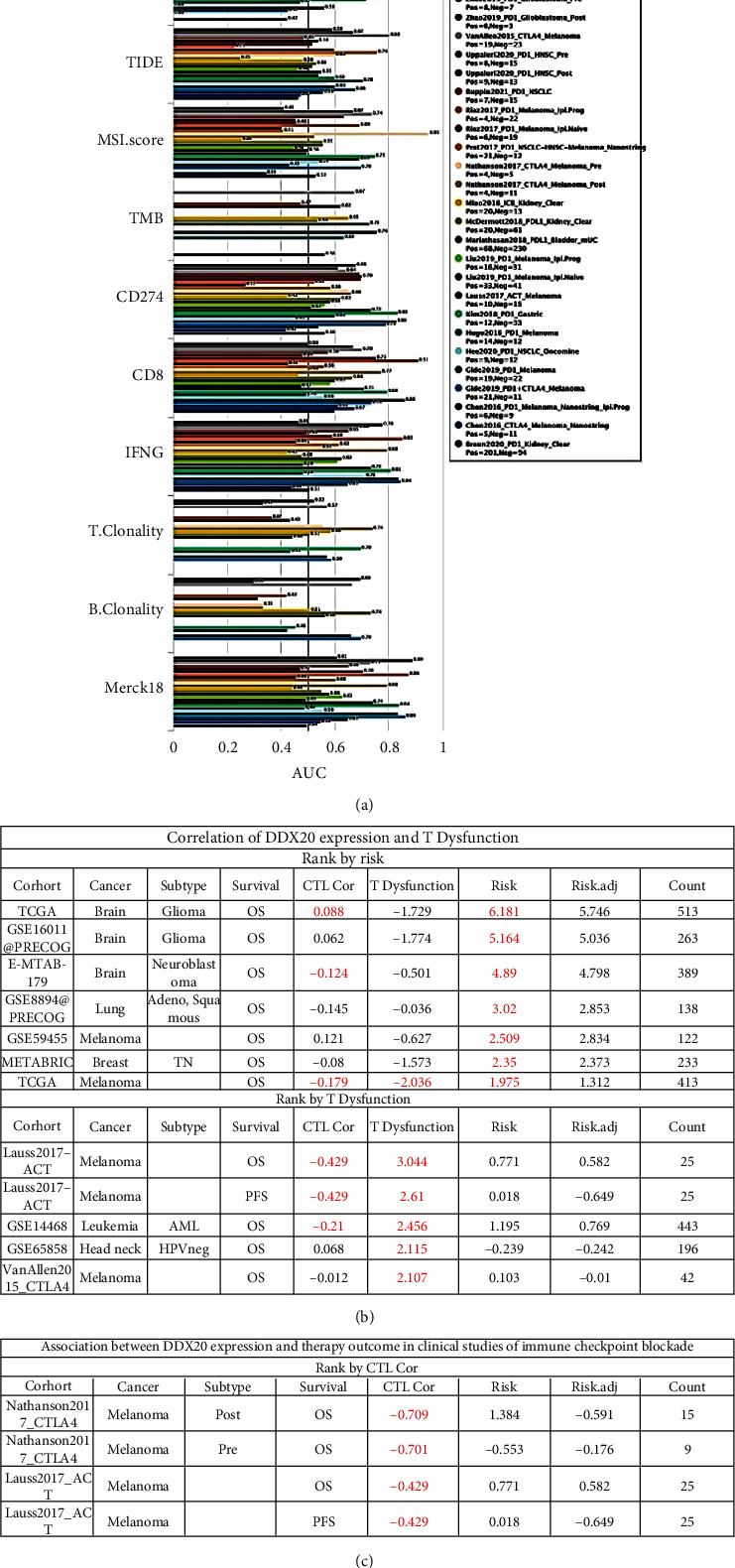
DDX20 expression independently predicts immunotherapy. (a) Bar plot showing the biomarker relevance of DDX20 compared to standardized cancer immune evasion biomarkers in immune checkpoint blockade (ICB) subcohorts. The area under the receiver operating characteristic curve (AUC) was applied to evaluate the predictive performances of the test biomarkers on the ICB response status. (b) Correlation between the DDX20 expression and cytotoxic T-cell levels (CTLs), dysfunctional T-cell phenotypes, and risk factors in multiple cancer cohorts. (c) Correlation between the DDX20 expression and cytotoxic T-cell levels (CTLs), dysfunctional T-cell phenotypes, and risk factors in multiple cancer cohorts received immune checkpoint blockade. Data was obtained via TIDE database. If the statistics in table are statistically significant (*P* < 0.05), the numbers will be redden with red font.

The area under the receiver operating characteristic curve (AUC) value is exhibited in Figure 6(a); these statistics can represent the predicted ICB responsiveness of DDX20 and existing biomarkers in 25 ICB cohorts. First, DDX20 alone had an AUC of >0.5 in 10 ICB cohorts. Compared with TIDE, MIS score, CD274, CD8, IFNG, and Merck18, DDX20 alone had a higher AUC (0.71) in head and neck squamous cell carcinoma (Uppaluri2020_PD1_HNSC_Pre). Compared with TIDE, MIS score, CD8, IFNG, and Merck18, DDX20 alone had a higher AUC (0.62) in head and neck squamous cell carcinoma (Uppaluri2020_PD1_HNSC_Post). Compared with TIDE, MIS score, CD274, CD8, IFNG, T.Clonality, B.Clonality, and Merck18, DDX20 alone had a higher AUC (0.62) in melanoma (Riaz2017_PD1_Mealnoma_lpi.Navie). Compared with TIDE, CD8, IFNG, T.Clonality, B.Clonality, and Merck18, DDX20 alone had a higher AUC (0.65) in melanoma (Nathanson2017_CTLA4_Mealnoma_Pre). Compared with TIDE, MSI score, CD274, CD8, IFNG, and Merck18, DDX20 alone had a higher AUC (0.68) in melanoma (Liu2019_PD1_Mealnoma_lip_Naive). Compared with TIDE, T.Clonality, and B.Clonality, DDX20 alone had a higher AUC (0.71) in gastric cancer (Kim2018_PD1_Gastric).

We also evaluate the effect of genetic alterations of DDX20 on dysfunctional T-cell phenotypes, cytotoxic T-cell levels (CTLs), and tumor patient outcomes using the Query module in TIDE. As shown in Figure 6(b), sorting by risk value, we found that high expressed DDX20 was a risk factor of poor prognosis in tumor of brain, lung, breast, and melanoma (*P* < 0.05). Next, we found positive, statistically significant correlations between expression of DDX20 and T dysfunction in 5 tumor cohorts. Analysis on two ICB cohort data (Nathanson2017_CTLA4, Lauss2017_ACT) showed that DDX20 was strongly positively correlated with CTLs.

### 3.6. The DDX20 Dependence and the EGFR Gene Effect at Hepatocellular Carcinoma (HCC) Cell Lines

Tumor cell-essential genes can be screened through CRISPR-based [18] and shRNA-based [19] genome editing. To investigate DDX20 essentiality in HCC cells, we leveraged CGPE, which can generate genetic dependencies of mRNA in tumor cells by pooled RNAi or CRISPR screening data. In our analysis, liver cancer cell lines were highly dependent on DDX20 (dependency score range −0.636 to −1.336). Among them, SNU182 and HUH7 cell lines both express high DDX20 with a high dependency score (-1.336 and -1.276, respectively).

**Figure 7:**
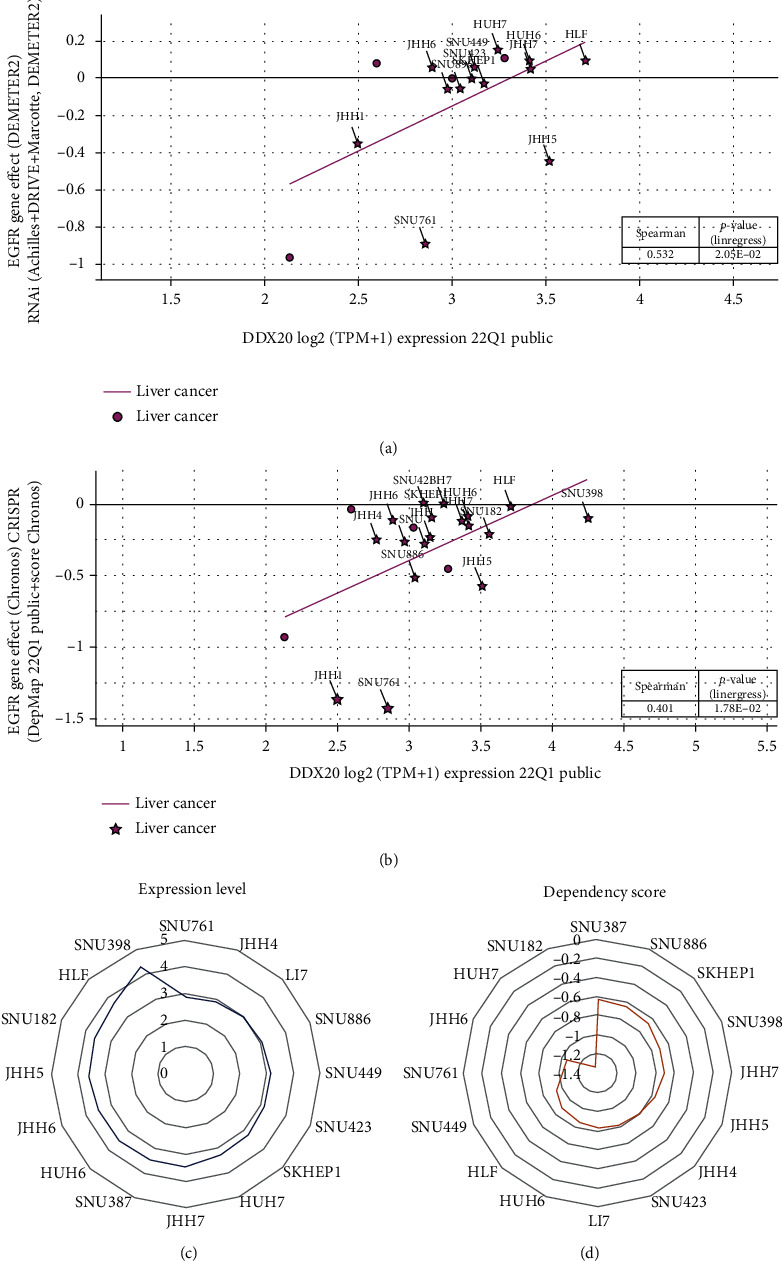
Analysis of DDX20 dependence and correlation between the expression of DDX20 and the gene effect of epidermal growth factor receptor (EGFR) in liver cancer cell lines. (a) DDX20 expression in liver tumor cell lines positive correlates with the gene effect of EGFR from the RNAi project. (b) DDX20 expression in liver tumor cell lines positive correlates with the gene effect of EGFR from the CRISPR project. Data was obtained via the DepMap database. (c, d) Expression and dependency score for DDX20 in liver tumor cell lines.

Given that knockout EGFR can improve the sensitivity of HCC cells to Lenvatinib in HCC cells [20], we utilized CRISPR and RNAi to alter DDX20 transcript levels and then to observe EGFR gene effect on HCC cell, which is implemented in the DepMap database. The results summarized in Figures 7(a) and 7(b) show that the EGFR gene effect is increased in 17 HCC cell lines along with the expression of DDX20 elevated. These data further support that DDX20 essentiality across liver tumor cell lines and aberrant DDX20 expression might influence LIHC patients to benefit from the first-line targeted therapy.

### 3.7. Coexpression and Regulatory Network Construction between DDX20 and METS in Liver Cancer

**Figure 8:**
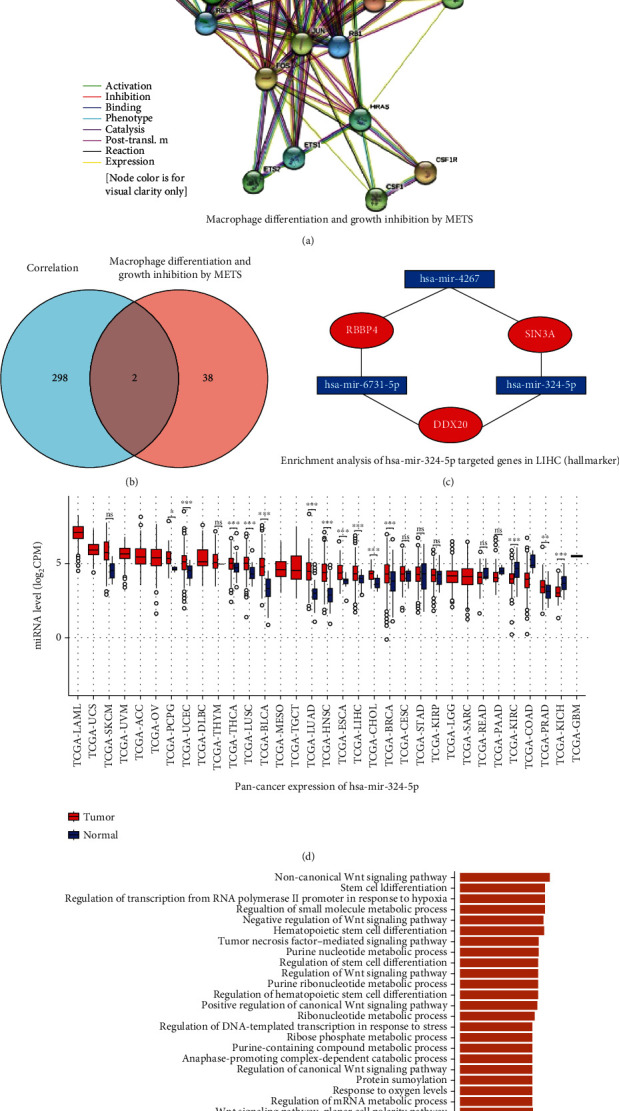
The construction of DDX20-related gene regulatory network. (a) DDX20 related the pathway network of macrophage differentiation and growth inhibition by METS. (b) Two genes were overlapped in the intersection of DDX20-positive correlated genes and genes that involved macrophage differentiation and growth inhibition by METS. (b) Three miRNA–two overlapped genes and DDX20 network generated by NetworkAnalyst software. The regulation network of 3 miRNAs (has-mir-4267, has-mir-6731-Sp, and has-mir-324-5p) and 3 mRNAs (DDX20, RBBP4, and SIN3A). (d) Pan-cancer expression analysis of has-mir-324-5p. (e) The has-mir-324-5p-targeted gene enrichment analysis via the CancerMIRNome database.

Tumor-associated macrophages are a major tumorigenic immune cell infiltrated in the tumorous environment [12]. We noted that DDX20 involved a significant macrophage differentiation pathway: DDX20 is related to the pathway network of macrophage differentiation and growth inhibition by METS (Figure 8(a)). In order to demonstrate the potential regulatory mechanism related to DDX20 in this pathway, we analyzed the intersection between the top 300 positively DDX20-correlated genes and the list of 40 genes that belong to the signaling pathway of macrophage differentiation and growth inhibition by METS. In this approach, we identified two important genes, RBBP4, and SIN3A (Figure 8(b)).

Then, we call the miRTarBase database through the NetworkAnalyst software to construct a miRNA-mRNA network, which contained RBBP4, SIN3A, and DDX20 gene. Finally, has-mir-4267, has-mir-6731-Sp, and has-mir-324-5p, the most important miRNA, are found in this network. Furthermore, the pancancer expression and the genes enrichment analysis on each miRNA were performed at the CancerMIRNome database (http://bioinfo.jialab-ucr.org/CancerMIRNome/). Importantly, has-mir-324-5p was found to have a significant differential expression in multiple cancer tissues (Figure 8(d)) In addition, has-mir-324-5p-targeted genes mainly enriched regulation of Wnt signaling pathway, tumor necrosis factor-mediated signaling pathway, and mRNA metabolic process (Figure 8(e)).

### 3.8. DDX20 Overexpression Facilitated the Malignant Behaviors and Oncogenic Signaling in Liver Cancer

The data presented above suggest DDX20 may serve as a promising therapeutic target in HCC; thus, we further define the biological meaning of DDX20. A total of six gene sets were used to perform GSEA analysis for DDX20, including Curated gene sets, Computational gene sets, Ontology gene sets, Oncogenic signature gene sets, Immunologic signature gene sets, and Hallmarker gene sets.

**Figure 9:**
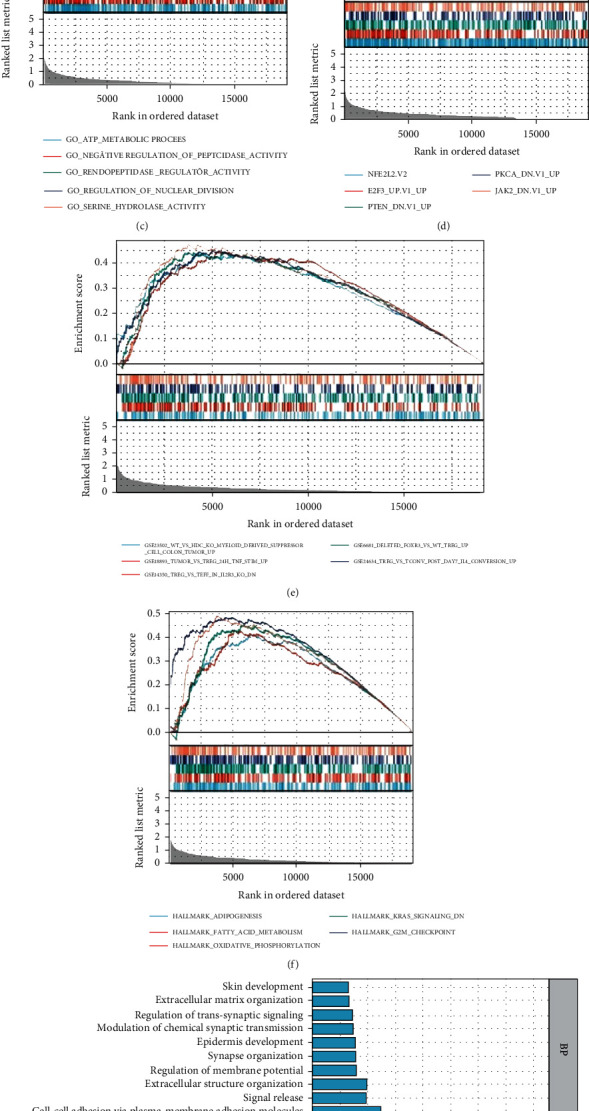
Functional enrichment analysis of DDX20-related genes in LIHC. (a–f) Gene Set Enrichment Analysis of DDX20-related genes and also present with top 5 annotation entries of every single gene sets (Curated gene sets, Computational gene sets, Ontology gene sets, Oncogenic signature gene sets, Immunologic signature gene sets and Hallmarker gene sets). (g) Gene Ontology analyses, including biological process, molecular function, and cellular component, were performed by clusterProfiler R package.

The GSEA results suggested that the most involved oncogenic pathways included liver cancer with H3K27ME3 and KOBAYASHI_EGFR_SIGNALING_24HR_DN (Figure 9(a)). Some cancer gene neighborhoods, such as GNF2_HPX, GNF2_HPN, and MORF_FLT1, were also significantly enriched (Figure 9(b)). Genes upregulated upon PTEN knockdown, PKCA knockdown, and JAK2 knockdown were mainly enriched (Figure 9(d)). Analysis of the Hallmark gene sets indicated significant enrichment of multiple oncogenic pathways, including the KRAS signaling and G2M checkpoint (Figure 9(f)).

Moreover, reports of the GO and KEGG analysis demonstrated that signal release pathways, the neuroactive ligand-receptor interaction pathway, metal ion transmembrane transporter activity, and ion channel complex were significantly related to DDX20 upregulated in liver cancer (Figure 9(g)). Overall, DDX20 might promote the proliferation and migration of oncogenic characteristics in liver cancer cells.

## 4. Discussion

DEAD-box RNA helicases engaged in various cellular processes and in numerous cancer have been embroiled in pro-proliferative and neoplastic transformation functions [21]. To date, the aberrantly activated DDX20 has been reported to be correlated with invasiveness and metastatic behavior in multiple tumors, including prostate cancer [22], breast cancer [23], and oral squamous cell carcinoma [24]. Here, we revealed that DDX20 is overexpressed in 1655 HCC tissues and 40 cirrhotic liver tissues, respectively, compared with normal samples, and that high DDX20 expression is linked to poor prognosis. Furthermore, we also detected a consistent trend that DDX20 was up-regulated in 40 liver cancer tissues from patients in our hospital. Below, we discuss the results in more detail.

According to the RNA-seq data from TCGA, we found that DDX20 expression level was increasing with increasing stage, but decreasing with weight. Aberrant expression of DDX20 affects the OS, PFI, and DSS in HCC patients, and poor survival was observed in those with high expression level.

Taking patients stratified according to the cirrhosis score, health status (albumin, height, weight, and BMI), and race, we analyzed those patients in different subgroups using the KM curve. In inadequate health patients, overexpressed DDX20 can predict worse OS. Improved survival following a diagnosis of liver cancer is an important task in modern medicine. The health status outcomes for the individual primary tumors are a powerful index for following patients after treatment exposures [25]. A previous study has reported the liver fibrosis is inversely correlated with overall survival in HCC patients [26]. In our results, upregulated DDX20 reflected a worse prognosis for patients with higher fibrosis scores. In addition, the majority of liver cancer deaths were mainly contributed by infected HBV/HCV patients [27], and hepatitis virus-related cirrhosis is common in Asia [28]. Given the above that we done a survival analysis for Asian patients and found that high DDX20 expression levels also predicted a shorted OS. These data suggest that DDX20 could be responsible for predicting prognosis in some subgroups of liver tumors.

Widespread loss of DNA methylation is a hallmark of human cancers and is often accompanied by activated oncogenes [29, 30]. Thus, we detected the methylation level in UALCAN and found a decreasing trend of the promoter methylation level of DDX20 with increasing tumor grade and stage. It is indicated that transcriptional activation of DDX20 is associated with lost DNA methylation.

Apoptosis resistance is closely correlated with carcinogenesis, affecting the prognosis of liver cancer patients [31]. TP53 is also known as a tumor suppressor gene and involved apoptosis of high proliferative tumor cell [32]. Previous genome-wide analyses suggest that gastric cancer patients with TP53 mutation carrier a distinct methylation signature and that is a key cancer susceptibility [33]. It is interesting to note that DDX20 promoter methylated relates to the TP53 mutation level for HCC closely. We hypothesize that this may be one of the ways in which DDX20 was deregulated. In addition, high expression of DDX20 may participate in TP53-mediated apoptosis of liver tumor cells.

The foremost influential factor in immunotherapy is the complexity tumor microenvironment (TME), and the differential immunophenotype was associated with the worst pathological status [34]. In this study, we reported that the DDX20 expression had a strong molecular connection with immune infiltrate statuses such as Treg, macrophages, DC, and other tumor-associated immune cells. As we know, immune checkpoints are one of the most important targets for immunotherapy strategies [35]. In our own further explored study, we found that a positive correlation exists between DDX20 and immune checkpoint, and a significant difference in TMEscore was also presented in the low DDX20 and high DDX20 group. It is suggested that there still has a crucial molecular mechanism for participating the interaction of DDX20 and TME. MDSCs are highly immunosuppressive in TME [36]. In HCC, the MDSC abundance has been positively correlated with DDX20, and the higher the value, the worse the prognosis. In addition, we used the TIDE algorithm to explore the relationship between the DDX20 and response to treatment in multiple cancer cohorts treated with immune checkpoint inhibitors. These findings provide a novel insight into antitumor immunity for HCC with highly expressed DDX20.

Epidermal growth factor receptor (EGFR) is a catalytic activator protooncogene that can trigger oncogenic transformation [37]. Previous research also reported that inhibiting EGFR phosphorylation levels can decrease tumor cell proliferation [38]. In view of that, the RNAi and CRISPR analyses were done through DepMap; we found DDX20 is an essential gene for EGFR in liver tumor cells scoring a high gene effect. According to above results, the DDX20 may be a potential predicted biomarker and EGFR target gene for liver cancer.

Interestingly, we noticed a DDX20 governing mechanism of cell proliferate, mitogenic Ets transcriptional suppressor (METS), can arrest the cell cycle of macrophages [39]. In particular, when taking the intersection of METS related gene set with DDX20 related HCC gene set and built the miRNA-RNA network, two key genes and three miRNAs were identified. Among them, has-mir-324-5p has been reported that involve the process of alternative macrophage activation [40]. We further uncovered that the has-mir-324-5p was aberrant expressed in pancancer and was associated with various cancerous signaling pathways. Therefore, DDX20 and the network construed in the present study have a great value to kill liver tumor cells and remodel TME.

The study further revealed several candidate pathways possibly regulated by DDX20, including the H3K27ME3, EGFR, PTEN, and JAK2 signaling pathways. The H3K27 methylation controls the extrachromosomal amplification of EGFR, driving the drug resistance for cancer [41]. Thus, our finding suggested that targeted DDX20 may be a therapeutic strategy for controlling EGFR copy number heterogeneity in cancer. Nevertheless, the current study also had some shortcomings. Patients with liver cancer in our trails all have hepatitis B virus infection, thereby limiting knowledge of potential relationships between nonneoplastic liver diseases and DDX20 expression. Mechanism of DDX20 in acting directly within cancer cells or tumor microenvironment still needed experiments for investigation. The prognostic analysis and function trials of DDX20 in HCC will be performed in our next work.

In summary, our study revealed that DDX20 was aberrantly overexpressed in liver cancer. High DDX20 expression is positively correlated with tumor stage and health condition and predicts a poor prognosis for HCC patients. Various immune analyses showed that DDX20 is a bright immunology marker in HCC. In addition, we identified DDX20 as an essential gene for EGFR in the liver cancer cells. Importantly, we also found that the has-mir-324-5p may play a critical role in the polarization and differentiation of macrophages together with DDX20 in HCC. Overall, our study highlighted the tumor immunology role of DDX20 in liver cancer and provided a series of novel insights into DDX20 in liver cancer.

## Data Availability

Publicly available datasets were analyzed in this study. These data can be found here: TCGA, UALCAN, TIMER, DepMap, and proteomicsDB databases.

## Supplementary Materials

Supplementary Materials Supplementary file 1: prognostic analysis of DDX20.
