# Supplementary material for: A Macrophage Differentiation-Mediated Gene: DDX20 as a Molecular Biomarker Encompassing the Tumor Microenvironment, Disease Staging, and Prognoses in Hepatocellular Carcinoma
Source: Oxid Med Cell Longev. 2022 Oct 5;2022:9971776. doi: 10.1155/2022/9971776 (PMC9556188; doi:10.1155/2022/9971776)
Supplement: Supplementary Materials — Supplementary file 1: prognostic analysis of DDX20. [file 9971776.f1.docx]

**Supplement.1 OS optimal cutoff value**

| sample_id | event | time | expr | gene | group |
| --- | --- | --- | --- | --- | --- |
| TCGA-DD-AADJ-01A-11R-A41C-07 | 1 | 35.5333333 | 1.06421248 | DDX20 | Low |
| TCGA-DD-A3A2-01A-11R-A213-07 | 2 | 71.0333333 | 1.16526421 | DDX20 | Low |
| TCGA-ES-A2HS-01A-11R-A180-07 | 2 | 22.9333333 | 1.17071244 | DDX20 | Low |
| TCGA-FV-A4ZP-01A-12R-A266-07 | 2 | 82.8666667 | 1.25593027 | DDX20 | Low |
| TCGA-DD-A3A7-01A-11R-A22L-07 | 2 | 13.9666667 | 1.26859986 | DDX20 | Low |
| TCGA-2Y-A9H1-01A-11R-A38B-07 | 2 | 40.9666667 | 1.27912311 | DDX20 | Low |
| TCGA-DD-AAEB-01A-11R-A41C-07 | 1 | 15.9333333 | 1.28265443 | DDX20 | Low |
| TCGA-G3-AAV0-01A-11R-A37K-07 | 1 | 15.8666667 | 1.29509613 | DDX20 | Low |
| TCGA-DD-AADS-01A-11R-A41C-07 | 1 | 15.8 | 1.31969782 | DDX20 | Low |
| TCGA-2Y-A9H3-01A-11R-A38B-07 | 1 | 50.5333333 | 1.33416283 | DDX20 | Low |
| TCGA-CC-5259-01A-31R-A213-07 | 1 | 8.33333333 | 1.35141987 | DDX20 | Low |
| TCGA-DD-A3A9-01A-11R-A266-07 | 2 | 31.0333333 | 1.3974612 | DDX20 | Low |
| TCGA-DD-AADO-01A-11R-A41C-07 | 1 | 15.1 | 1.40066352 | DDX20 | Low |
| TCGA-ES-A2HT-01A-12R-A180-07 | 2 | 14.6 | 1.43357022 | DDX20 | Low |
| TCGA-WQ-AB4B-01A-11R-A41C-07 | 1 | 13.1666667 | 1.45679822 | DDX20 | Low |
| TCGA-RC-A7SH-01A-11R-A38B-07 | 1 | 15.6 | 1.46730412 | DDX20 | Low |
| TCGA-2Y-A9H6-01A-11R-A39D-07 | 1 | 11.9 | 1.49686387 | DDX20 | Low |
| TCGA-DD-A3A3-01A-11R-A22L-07 | 2 | 17.8333333 | 1.5023969 | DDX20 | Low |
| TCGA-2Y-A9GU-01A-11R-A38B-07 | 1 | 64.6333333 | 1.51033494 | DDX20 | Low |
| TCGA-DD-A73G-01A-22R-A32O-07 | 1 | 115.933333 | 1.52872998 | DDX20 | Low |
| TCGA-DD-A39X-01A-11R-A213-07 | 2 | 56.4666667 | 1.5473309 | DDX20 | Low |
| TCGA-NI-A4U2-01A-11R-A28V-07 | 2 | 59.7 | 1.5491896 | DDX20 | Low |
| TCGA-DD-A11C-01A-11R-A131-07 | 1 | 22.0666667 | 1.55170997 | DDX20 | Low |
| TCGA-BC-A10R-01A-11R-A131-07 | 2 | 10.2666667 | 1.55641699 | DDX20 | Low |
| TCGA-DD-AAE1-01A-11R-A41C-07 | 1 | 18.4 | 1.56164519 | DDX20 | Low |
| TCGA-DD-AADU-01A-11R-A41C-07 | 1 | 18.4666667 | 1.58463853 | DDX20 | Low |
| TCGA-NI-A8LF-01A-11R-A36F-07 | 1 | 26.6333333 | 1.60386912 | DDX20 | Low |
| TCGA-DD-AAC9-01A-11R-A41C-07 | 1 | 11.5666667 | 1.61501197 | DDX20 | Low |
| TCGA-DD-A4NG-01A-11R-A27V-07 | 2 | 26.7333333 | 1.6262543 | DDX20 | Low |
| TCGA-DD-A119-01A-11R-A131-07 | 2 | 7.43333333 | 1.63397767 | DDX20 | Low |
| TCGA-DD-A11B-01A-11R-A131-07 | 2 | 0.46666667 | 1.6353811 | DDX20 | Low |
| TCGA-DD-AADN-01A-11R-A41C-07 | 1 | 29.9333333 | 1.63830221 | DDX20 | Low |
| TCGA-DD-AADM-01A-11R-A41C-07 | 2 | 0.4 | 1.64586394 | DDX20 | Low |
| TCGA-DD-A1EK-01A-11R-A213-07 | 2 | 18.6 | 1.65049789 | DDX20 | Low |
| TCGA-EP-A3JL-01A-11R-A213-07 | 1 | 10.1 | 1.65474362 | DDX20 | Low |
| TCGA-G3-A5SM-01A-12R-A28V-07 | 1 | 17.3333333 | 1.6669265 | DDX20 | Low |
| TCGA-2Y-A9GT-01A-11R-A38B-07 | 2 | 54.1333333 | 1.66704031 | DDX20 | Low |
| TCGA-RC-A7SF-01A-11R-A352-07 | 1 | 19.3 | 1.67023264 | DDX20 | Low |
| TCGA-WX-AA47-01A-11R-A39D-07 | 2 | 18.5333333 | 1.67117576 | DDX20 | Low |
| TCGA-DD-A11D-01A-11R-A131-07 | 2 | 52 | 1.67386501 | DDX20 | Low |
| TCGA-G3-A7M5-01A-11R-A33R-07 | 1 | 14.9 | 1.67885141 | DDX20 | Low |
| TCGA-KR-A7K7-01A-11R-A33J-07 | 1 | 31.7 | 1.68311326 | DDX20 | Low |
| TCGA-DD-A4NP-01A-11R-A28V-07 | 1 | 110.266667 | 1.69338093 | DDX20 | Low |
| TCGA-EP-A2KB-01A-11R-A180-07 | 2 | 19.8666667 | 1.69444014 | DDX20 | Low |
| TCGA-5C-A9VH-01A-11R-A37K-07 | 1 | 10.7333333 | 1.71231545 | DDX20 | Low |
| TCGA-DD-AAE3-01A-11R-A41C-07 | 1 | 18.8666667 | 1.72677035 | DDX20 | Low |
| TCGA-DD-A3A6-01A-11R-A22L-07 | 2 | 108.6 | 1.72700077 | DDX20 | Low |
| TCGA-LG-A9QD-01A-11R-A38B-07 | 1 | 12.2 | 1.72751901 | DDX20 | Low |
| TCGA-G3-A5SK-01A-11R-A27V-07 | 1 | 24.8 | 1.73190712 | DDX20 | Low |
| TCGA-DD-A4NL-01A-11R-A28V-07 | 1 | 57.0333333 | 1.73620405 | DDX20 | Low |
| TCGA-BC-A10S-01A-22R-A131-07 | 2 | 47.4333333 | 1.75346611 | DDX20 | Low |
| TCGA-G3-AAUZ-01A-11R-A38B-07 | 1 | 16 | 1.76058938 | DDX20 | Low |
| TCGA-DD-AACD-01A-11R-A41C-07 | 2 | 12.7 | 1.76241874 | DDX20 | Low |
| TCGA-BC-A110-01A-11R-A131-07 | 2 | 70.5333333 | 1.775718 | DDX20 | Low |
| TCGA-RC-A7SB-01A-21R-A352-07 | 1 | 19.6 | 1.78342143 | DDX20 | Low |
| TCGA-DD-A39Z-01A-11R-A213-07 | 2 | 20.0333333 | 1.79499311 | DDX20 | Low |
| TCGA-FV-A3R3-01A-11R-A22L-07 | 2 | 12.2 | 1.80245974 | DDX20 | Low |
| TCGA-G3-AAV6-01A-21R-A37K-07 | 2 | 2.16666667 | 1.80575721 | DDX20 | Low |
| TCGA-DD-AADF-01A-11R-A41C-07 | 2 | 3.83333333 | 1.81358773 | DDX20 | Low |
| TCGA-DD-AADK-01A-11R-A41C-07 | 1 | 34.9666667 | 1.81421643 | DDX20 | Low |
| TCGA-G3-A3CJ-01A-11R-A213-07 | 1 | 19.8 | 1.81619652 | DDX20 | Low |
| TCGA-G3-A25Z-01A-11R-A16W-07 | 1 | 21.8333333 | 1.81932796 | DDX20 | Low |
| TCGA-DD-A4NE-01A-11R-A27V-07 | 2 | 22 | 1.82307083 | DDX20 | Low |
| TCGA-K7-A6G5-01A-11R-A311-07 | 1 | 17.0666667 | 1.82630909 | DDX20 | Low |
| TCGA-G3-A3CI-01A-11R-A213-07 | 1 | 6 | 1.83228142 | DDX20 | Low |
| TCGA-CC-A123-01A-11R-A131-07 | 1 | 7.3 | 1.8325137 | DDX20 | Low |
| TCGA-DD-AADA-01A-11R-A41C-07 | 1 | 41.1 | 1.8352515 | DDX20 | Low |
| TCGA-DD-AAE4-01A-11R-A41C-07 | 1 | 20.2666667 | 1.84067771 | DDX20 | Low |
| TCGA-DD-A73A-01A-12R-A32O-07 | 1 | 24.2666667 | 1.84249609 | DDX20 | Low |
| TCGA-HP-A5MZ-01A-21R-A27V-07 | 2 | 3.03333333 | 1.8425587 | DDX20 | Low |
| TCGA-2Y-A9GW-01A-11R-A38B-07 | 2 | 42.3666667 | 1.84391737 | DDX20 | Low |
| TCGA-DD-A4NI-01A-11R-A27V-07 | 1 | 27.2 | 1.86146197 | DDX20 | Low |
| TCGA-WJ-A86L-01A-12R-A39D-07 | 1 | 11.5 | 1.87332093 | DDX20 | Low |
| TCGA-DD-AAE7-01A-11R-A41C-07 | 1 | 21.4666667 | 1.88061718 | DDX20 | Low |
| TCGA-DD-AAVW-01A-11R-A41C-07 | 1 | 77.2333333 | 1.88507653 | DDX20 | Low |
| TCGA-DD-AACI-01A-11R-A41C-07 | 1 | 53.9333333 | 1.8859475 | DDX20 | Low |
| TCGA-EP-A2KC-01A-11R-A213-07 | 2 | 0.63333333 | 1.88937676 | DDX20 | Low |
| TCGA-DD-A3A8-01A-11R-A22L-07 | 2 | 0.36666667 | 1.89197789 | DDX20 | Low |
| TCGA-DD-AACE-01A-11R-A41C-07 | 1 | 72.8 | 1.89855749 | DDX20 | Low |
| TCGA-KR-A7K2-01A-12R-A33R-07 | 1 | 27.6333333 | 1.89881097 | DDX20 | Low |
| TCGA-DD-A39V-01A-11R-A213-07 | 2 | 21.4333333 | 1.90960075 | DDX20 | Low |
| TCGA-G3-A3CH-01A-11R-A22L-07 | 1 | 26 | 1.91064494 | DDX20 | Low |
| TCGA-CC-A7IF-01A-11R-A33J-07 | 2 | 21.6333333 | 1.91110577 | DDX20 | Low |
| TCGA-MI-A75C-01A-11R-A32O-07 | 1 | 9.7 | 1.91900343 | DDX20 | Low |
| TCGA-BC-A69H-01A-11R-A311-07 | 1 | 14.8 | 1.92523076 | DDX20 | Low |
| TCGA-K7-A5RF-01A-11R-A28V-07 | 1 | 21.0333333 | 1.92642247 | DDX20 | Low |
| TCGA-BD-A3ER-01A-11R-A213-07 | 1 | 37.1666667 | 1.92819697 | DDX20 | Low |
| TCGA-DD-AAEI-01A-11R-A41C-07 | 1 | 51.0333333 | 1.92919097 | DDX20 | Low |
| TCGA-FV-A495-01A-11R-A266-07 | 1 | 0.03333333 | 1.93166149 | DDX20 | Low |
| TCGA-DD-AACW-01A-11R-A41C-07 | 1 | 47.4666667 | 1.93227997 | DDX20 | Low |
| TCGA-DD-AAVR-01A-11R-A41C-07 | 1 | 83.7666667 | 1.93472901 | DDX20 | Low |
| TCGA-DD-AACT-01A-11R-A41C-07 | 1 | 52.0666667 | 1.94267086 | DDX20 | Low |
| TCGA-ZS-A9CD-01A-11R-A37K-07 | 2 | 46.2 | 1.94312951 | DDX20 | Low |
| TCGA-DD-AAEG-01A-11R-A39D-07 | 1 | 23.9666667 | 1.95077057 | DDX20 | Low |
| TCGA-WX-AA46-01A-11R-A39D-07 | 1 | 25.2 | 1.95098088 | DDX20 | Low |
| TCGA-DD-AAE9-01A-11R-A41C-07 | 1 | 24.0666667 | 1.95367449 | DDX20 | Low |
| TCGA-G3-A7M8-01A-11R-A33R-07 | 1 | 14.3333333 | 1.95872743 | DDX20 | Low |
| TCGA-G3-AAV5-01A-11R-A37K-07 | 1 | 11.8 | 1.96055629 | DDX20 | Low |
| TCGA-FV-A3I0-01A-11R-A22L-07 | 1 | 28.2666667 | 1.97020285 | DDX20 | Low |
| TCGA-2Y-A9H4-01A-11R-A38B-07 | 1 | 48.4 | 1.99854291 | DDX20 | Low |
| TCGA-DD-AAVQ-01A-11R-A41C-07 | 1 | 90.9333333 | 2.00592941 | DDX20 | Low |
| TCGA-DD-A1ED-01A-11R-A155-07 | 1 | 76.7 | 2.00717668 | DDX20 | Low |
| TCGA-5R-AAAM-01A-12R-A41C-07 | 2 | 1.53333333 | 2.00737971 | DDX20 | Low |
| TCGA-DD-AAD2-01A-11R-A41C-07 | 1 | 21.9333333 | 2.01474322 | DDX20 | Low |
| TCGA-DD-A115-01A-11R-A131-07 | 2 | 84.7333333 | 2.01624575 | DDX20 | Low |
| TCGA-MI-A75G-01A-11R-A32O-07 | 1 | 23.2666667 | 2.01787599 | DDX20 | Low |
| TCGA-DD-A3A4-01A-11R-A22L-07 | 2 | 20.4 | 2.03754666 | DDX20 | Low |
| TCGA-DD-AACY-01A-11R-A41C-07 | 1 | 48.3333333 | 2.03755383 | DDX20 | Low |
| TCGA-RC-A6M6-01A-11R-A32O-07 | 1 | 0.3 | 2.04509128 | DDX20 | Low |
| TCGA-MR-A8JO-01A-12R-A36F-07 | 1 | 11 | 2.04534158 | DDX20 | Low |
| TCGA-DD-A118-01A-11R-A131-07 | 1 | 114.566667 | 2.04763012 | DDX20 | Low |
| TCGA-BD-A2L6-01A-11R-A213-07 | 1 | 45.4333333 | 2.04980543 | DDX20 | Low |
| TCGA-MR-A520-01A-11R-A266-07 | 1 | 7.63333333 | 2.05514707 | DDX20 | Low |
| TCGA-CC-A3MB-01A-11R-A213-07 | 2 | 10.5 | 2.05762217 | DDX20 | Low |
| TCGA-5C-AAPD-01A-21R-A39D-07 | 1 | 0.66666667 | 2.05811555 | DDX20 | Low |
| TCGA-O8-A75V-01A-11R-A32O-07 | 1 | 17.9333333 | 2.06548958 | DDX20 | Low |
| TCGA-DD-A113-01A-11R-A131-07 | 1 | 80.8333333 | 2.06874972 | DDX20 | Low |
| TCGA-DD-AAEA-01A-11R-A41C-07 | 1 | 19.1666667 | 2.07086742 | DDX20 | Low |
| TCGA-DD-A116-01A-11R-A131-07 | 2 | 54.0666667 | 2.07171149 | DDX20 | Low |
| TCGA-BC-A10X-01A-11R-A131-07 | 2 | 25.6666667 | 2.07414015 | DDX20 | Low |
| TCGA-ED-A7PZ-01A-11R-A33R-07 | 1 | 0.2 | 2.07757736 | DDX20 | Low |
| TCGA-BC-A69I-01A-11R-A311-07 | 1 | 12.9 | 2.07805984 | DDX20 | Low |
| TCGA-ZS-A9CG-01A-11R-A37K-07 | 1 | 11.3666667 | 2.07959572 | DDX20 | Low |
| TCGA-DD-AAW1-01A-11R-A41C-07 | 1 | 66.3 | 2.0867939 | DDX20 | Low |
| TCGA-MI-A75E-01A-11R-A32O-07 | 1 | 16.9 | 2.09083744 | DDX20 | Low |
| TCGA-G3-A7M7-01A-12R-A352-07 | 1 | 12.0333333 | 2.0971436 | DDX20 | Low |
| TCGA-DD-A4NV-01A-11R-A311-07 | 1 | 79.9333333 | 2.09864647 | DDX20 | Low |
| TCGA-DD-AAEK-01A-11R-A41C-07 | 1 | 35.5666667 | 2.10325697 | DDX20 | Low |
| TCGA-CC-A3MC-01A-11R-A22L-07 | 1 | 12.1 | 2.10581808 | DDX20 | Low |
| TCGA-2Y-A9H5-01A-11R-A38B-07 | 2 | 18.5 | 2.12304557 | DDX20 | Low |
| TCGA-DD-AACA-02B-11R-A41C-07 | 1 | 76.7 | 2.12679422 | DDX20 | Low |
| TCGA-XR-A8TC-01A-11R-A36F-07 | 1 | 44.6333333 | 2.13003238 | DDX20 | Low |
| TCGA-DD-AACG-01A-11R-A41C-07 | 2 | 15.6333333 | 2.1312006 | DDX20 | Low |
| TCGA-BC-A10T-01A-11R-A131-07 | 2 | 27.9 | 2.13162481 | DDX20 | Low |
| TCGA-DD-A1EI-01A-11R-A131-07 | 1 | 6.1 | 2.13198571 | DDX20 | Low |
| TCGA-DD-AAD6-01A-11R-A41C-07 | 1 | 22.4 | 2.13510422 | DDX20 | Low |
| TCGA-DD-A3A5-01A-11R-A22L-07 | 2 | 104.166667 | 2.1355512 | DDX20 | Low |
| TCGA-DD-AACO-01A-11R-A41C-07 | 1 | 62.5333333 | 2.13725626 | DDX20 | Low |
| TCGA-DD-AACU-01A-11R-A41C-07 | 1 | 52.2333333 | 2.13767705 | DDX20 | Low |
| TCGA-DD-AAE0-01A-11R-A41C-07 | 1 | 18.5 | 2.13935378 | DDX20 | Low |
| TCGA-G3-A25V-01A-11R-A16W-07 | 1 | 28.6666667 | 2.14745332 | DDX20 | Low |
| TCGA-DD-AAE6-01A-11R-A41C-07 | 1 | 4.7 | 2.15565003 | DDX20 | Low |
| TCGA-XR-A8TD-01A-12R-A39D-07 | 1 | 34.3333333 | 2.15906288 | DDX20 | Low |
| TCGA-DD-AAD3-01A-11R-A41C-07 | 1 | 43.1666667 | 2.16604645 | DDX20 | Low |
| TCGA-EP-A2KA-01A-11R-A180-07 | 2 | 20.9 | 2.16935578 | DDX20 | Low |
| TCGA-DD-A1EH-01A-11R-A131-07 | 1 | 49.8333333 | 2.17955971 | DDX20 | Low |
| TCGA-RC-A7SK-01A-11R-A352-07 | 1 | 15.7333333 | 2.19200955 | DDX20 | Low |
| TCGA-RC-A7S9-01A-11R-A33R-07 | 1 | 21.3333333 | 2.19223796 | DDX20 | Low |
| TCGA-DD-AACQ-01A-11R-A41C-07 | 2 | 14.4 | 2.19242032 | DDX20 | Low |
| TCGA-ZP-A9CY-01A-11R-A38B-07 | 1 | 26.0666667 | 2.20261411 | DDX20 | Low |
| TCGA-BC-A5W4-01A-11R-A28V-07 | 2 | 18.2333333 | 2.20780244 | DDX20 | Low |
| TCGA-DD-A4NB-01A-12R-A266-07 | 1 | 32.9666667 | 2.20842151 | DDX20 | Low |
| TCGA-DD-A3A1-01A-11R-A213-07 | 2 | 7.76666667 | 2.21299134 | DDX20 | Low |
| TCGA-G3-A25X-01A-11R-A16W-07 | 1 | 59.3 | 2.21446914 | DDX20 | Low |
| TCGA-GJ-A6C0-01A-12R-A311-07 | 2 | 1.03333333 | 2.21803674 | DDX20 | Low |
| TCGA-MI-A75H-01A-11R-A32O-07 | 1 | 24.9 | 2.21857284 | DDX20 | Low |
| TCGA-G3-A3CG-01A-11R-A213-07 | 1 | 22.4333333 | 2.22601874 | DDX20 | Low |
| TCGA-ED-A7PY-01A-11R-A33R-07 | 1 | 13 | 2.22685311 | DDX20 | Low |
| TCGA-2Y-A9H9-01A-21R-A39D-07 | 1 | 23.2333333 | 2.23072292 | DDX20 | Low |
| TCGA-WQ-A9G7-01A-11R-A37K-07 | 1 | 1 | 2.23117955 | DDX20 | Low |
| TCGA-DD-AAW2-01A-11R-A41C-07 | 1 | 61.8333333 | 2.23619973 | DDX20 | Low |
| TCGA-DD-AAE2-01A-11R-A41C-07 | 1 | 21.2666667 | 2.2368352 | DDX20 | Low |
| TCGA-ZP-A9D1-01A-11R-A38B-07 | 1 | 0.7 | 2.25444745 | DDX20 | Low |
| TCGA-DD-AACC-01A-11R-A41C-07 | 2 | 56.1666667 | 2.25558876 | DDX20 | Low |
| TCGA-GJ-A3OU-01A-31R-A38B-07 | 1 | 29.3 | 2.25846097 | DDX20 | Low |
| TCGA-UB-A7MF-01A-11R-A33J-07 | 2 | 7.13333333 | 2.25945197 | DDX20 | Low |
| TCGA-KR-A7K8-01A-11R-A33J-07 | 1 | 30.2 | 2.25982063 | DDX20 | Low |
| TCGA-DD-A73F-01A-11R-A32O-07 | 1 | 36.1666667 | 2.26182069 | DDX20 | Low |
| TCGA-DD-A4NK-01A-11R-A28V-07 | 2 | 40.3333333 | 2.26976828 | DDX20 | Low |
| TCGA-G3-A25S-01A-11R-A16W-07 | 2 | 13.8666667 | 2.27031298 | DDX20 | Low |
| TCGA-DD-AADQ-01A-11R-A41C-07 | 1 | 14.5333333 | 2.27348195 | DDX20 | Low |
| TCGA-DD-AAEH-01A-11R-A41C-07 | 1 | 26.1333333 | 2.27445258 | DDX20 | Low |
| TCGA-EP-A26S-01A-11R-A16W-07 | 1 | 20.2666667 | 2.27479464 | DDX20 | Low |
| TCGA-ZP-A9D4-01A-11R-A37K-07 | 1 | 13.1666667 | 2.28266078 | DDX20 | Low |
| TCGA-UB-A7MD-01A-12R-A352-07 | 2 | 1.73333333 | 2.29409094 | DDX20 | Low |
| TCGA-G3-A25T-01A-11R-A16W-07 | 1 | 51.7666667 | 2.29638197 | DDX20 | Low |
| TCGA-T1-A6J8-01A-11R-A32O-07 | 1 | 0.76666667 | 2.29783041 | DDX20 | Low |
| TCGA-ZS-A9CF-01A-11R-A38B-07 | 1 | 80.4 | 2.29894047 | DDX20 | Low |
| TCGA-G3-AAV2-01A-11R-A37K-07 | 1 | 12.4 | 2.30842601 | DDX20 | Low |
| TCGA-DD-A4NH-01A-11R-A27V-07 | 1 | 30.5666667 | 2.30906933 | DDX20 | Low |
| TCGA-FV-A496-01A-11R-A266-07 | 1 | 0.33333333 | 2.31474076 | DDX20 | Low |
| TCGA-UB-AA0V-01A-11R-A38B-07 | 1 | 10.4666667 | 2.31963818 | DDX20 | Low |
| TCGA-DD-AADG-01A-11R-A41C-07 | 1 | 38.1666667 | 2.32085513 | DDX20 | Low |
| TCGA-5R-AA1D-01A-11R-A38B-07 | 1 | 14.9666667 | 2.32095323 | DDX20 | Low |
| TCGA-BC-A10Q-01A-11R-A131-07 | 2 | 37.8333333 | 2.32704767 | DDX20 | Low |
| TCGA-ZS-A9CE-01A-11R-A37K-07 | 1 | 41.3666667 | 2.33780245 | DDX20 | Low |
| TCGA-DD-AAVU-01A-11R-A41C-07 | 1 | 73.4 | 2.34320727 | DDX20 | Low |
| TCGA-G3-AAV3-01A-11R-A37K-07 | 1 | 13.7333333 | 2.34369174 | DDX20 | Low |
| TCGA-RC-A6M5-01A-11R-A32O-07 | 1 | 0.5 | 2.34605602 | DDX20 | Low |
| TCGA-2Y-A9GZ-01A-11R-A39D-07 | 2 | 28.2666667 | 2.35638238 | DDX20 | Low |
| TCGA-CC-A7IE-01A-21R-A38B-07 | 2 | 7.23333333 | 2.35910521 | DDX20 | Low |
| TCGA-KR-A7K0-01A-12R-A33R-07 | 2 | 2.16666667 | 2.36314355 | DDX20 | Low |
| TCGA-DD-AAVX-01A-11R-A41C-07 | 1 | 57.2666667 | 2.36368313 | DDX20 | Low |
| TCGA-ZP-A9D0-01A-11R-A37K-07 | 1 | 36.3666667 | 2.3669836 | DDX20 | Low |
| TCGA-DD-AACN-01A-11R-A41C-07 | 1 | 43.4 | 2.36741985 | DDX20 | Low |
| TCGA-2Y-A9H8-01A-11R-A39D-07 | 2 | 21.1 | 2.37191745 | DDX20 | Low |
| TCGA-CC-A9FW-01A-11R-A37K-07 | 1 | 8.26666667 | 2.38038362 | DDX20 | Low |
| TCGA-DD-A1EG-01A-11R-A213-07 | 2 | 45.7333333 | 2.38077114 | DDX20 | Low |
| TCGA-CC-A9FS-01A-11R-A37K-07 | 1 | 7.03333333 | 2.38198901 | DDX20 | Low |
| TCGA-DD-AACF-01A-11R-A41C-07 | 2 | 12.1666667 | 2.38768645 | DDX20 | Low |
| TCGA-DD-AAED-01A-12R-A41C-07 | 1 | 25.4333333 | 2.39434918 | DDX20 | Low |
| TCGA-HP-A5N0-01A-11R-A28V-07 | 2 | 38.2333333 | 2.39688455 | DDX20 | Low |
| TCGA-RC-A6M4-01A-11R-A32O-07 | 1 | 0.73333333 | 2.39808537 | DDX20 | Low |
| TCGA-ED-A627-01A-12R-A311-07 | 1 | 14.1 | 2.40010195 | DDX20 | Low |
| TCGA-ED-A4XI-01A-11R-A266-07 | 1 | 27.3 | 2.40253189 | DDX20 | Low |
| TCGA-BC-A10Z-01A-11R-A131-07 | 2 | 1.13333333 | 2.41348937 | DDX20 | Low |
| TCGA-DD-A73B-01A-12R-A32O-07 | 2 | 9.43333333 | 2.41876534 | DDX20 | Low |
| TCGA-RG-A7D4-01A-12R-A33R-07 | 1 | 36.6 | 2.42090174 | DDX20 | Low |
| TCGA-CC-A5UC-01A-11R-A28V-07 | 2 | 11.5666667 | 2.42408522 | DDX20 | Low |
| TCGA-3K-AAZ8-01A-12R-A39D-07 | 1 | 13.2 | 2.42503516 | DDX20 | Low |
| TCGA-G3-A5SI-01A-31R-A27V-07 | 2 | 25.6 | 2.42776141 | DDX20 | Low |
| TCGA-DD-A4NQ-01A-21R-A28V-07 | 2 | 12.4333333 | 2.42900478 | DDX20 | Low |
| TCGA-DD-AACJ-01A-11R-A41C-07 | 1 | 70.0666667 | 2.43955117 | DDX20 | Low |
| TCGA-ED-A7XO-01A-11R-A352-07 | 1 | 14.2333333 | 2.44054676 | DDX20 | Low |
| TCGA-2Y-A9GV-01A-11R-A38B-07 | 2 | 84.4 | 2.4446353 | DDX20 | Low |
| TCGA-PD-A5DF-01A-11R-A27V-07 | 2 | 21.3 | 2.44699301 | DDX20 | Low |
| TCGA-DD-AAVP-01A-11R-A41C-07 | 1 | 91.7333333 | 2.45585713 | DDX20 | Low |
| TCGA-DD-A1EB-01A-11R-A131-07 | 1 | 67.2333333 | 2.45707609 | DDX20 | Low |
| TCGA-FV-A2QR-01A-11R-A213-07 | 2 | 19.3666667 | 2.4657471 | DDX20 | Low |
| TCGA-ED-A66Y-01A-11R-A311-07 | 2 | 9.86666667 | 2.46797307 | DDX20 | Low |
| TCGA-FV-A2QQ-01A-11R-A22L-07 | 1 | 24.3 | 2.46960615 | DDX20 | Low |
| TCGA-2Y-A9H0-01A-11R-A38B-07 | 1 | 122.5 | 2.47385179 | DDX20 | Low |
| TCGA-ED-A8O6-01A-11R-A36F-07 | 2 | 1.86666667 | 2.47501249 | DDX20 | Low |
| TCGA-DD-AADR-01A-11R-A41C-07 | 1 | 67.6 | 2.48074529 | DDX20 | Low |
| TCGA-K7-A5RG-01A-11R-A28V-07 | 1 | 17.3 | 2.48327409 | DDX20 | Low |
| TCGA-G3-A25Y-01A-11R-A16W-07 | 2 | 15.0666667 | 2.48470336 | DDX20 | Low |
| TCGA-LG-A9QC-01A-11R-A37K-07 | 1 | 14.1666667 | 2.48807888 | DDX20 | Low |
| TCGA-DD-A4NO-01A-11R-A28V-07 | 1 | 74.8333333 | 2.48969148 | DDX20 | Low |
| TCGA-CC-A7IH-01A-11R-A33J-07 | 1 | 12.1666667 | 2.49013383 | DDX20 | Low |
| TCGA-DD-A4NN-01A-11R-A28V-07 | 2 | 29.9666667 | 2.49027225 | DDX20 | Low |
| TCGA-DD-AADW-01A-11R-A39D-07 | 1 | 19.5666667 | 2.49097416 | DDX20 | Low |
| TCGA-DD-A73C-01A-12R-A33J-07 | 1 | 23.3666667 | 2.4929807 | DDX20 | Low |
| TCGA-BW-A5NO-01A-11R-A27V-07 | 1 | 0.66666667 | 2.49943225 | DDX20 | Low |
| TCGA-WX-AA44-01A-11R-A39D-07 | 1 | 20.5 | 2.50203007 | DDX20 | Low |
| TCGA-2Y-A9H7-01A-11R-A39D-07 | 1 | 38.9333333 | 2.5041661 | DDX20 | Low |
| TCGA-DD-AACS-01A-11R-A41C-07 | 1 | 60.1333333 | 2.50757931 | DDX20 | Low |
| TCGA-CC-A7IL-01A-11R-A33R-07 | 2 | 9.26666667 | 2.51076097 | DDX20 | Low |
| TCGA-ED-A459-01A-11R-A266-07 | 1 | 30.3333333 | 2.51488025 | DDX20 | Low |
| TCGA-DD-AADL-01A-11R-A41C-07 | 1 | 21.2 | 2.51731886 | DDX20 | Low |
| TCGA-2Y-A9HB-01A-11R-A39D-07 | 1 | 8.66666667 | 2.5184042 | DDX20 | Low |
| TCGA-2V-A95S-01A-11R-A37K-07 | 1 |  | 2.52302893 | DDX20 | Low |
| TCGA-DD-A73D-01A-12R-A32O-07 | 1 | 23.1 | 2.52527239 | DDX20 | Low |
| TCGA-DD-AAD5-01A-11R-A41C-07 | 1 | 44.8333333 | 2.52700514 | DDX20 | Low |
| TCGA-DD-AAEE-01A-11R-A41C-07 | 1 | 27 | 2.53872832 | DDX20 | Low |
| TCGA-DD-AAVY-01A-11R-A41C-07 | 1 | 65.6666667 | 2.54041429 | DDX20 | Low |
| TCGA-DD-A4ND-01A-11R-A266-07 | 1 | 91.5333333 | 2.55027696 | DDX20 | Low |
| TCGA-2Y-A9GX-01A-11R-A38B-07 | 1 | 81.4 | 2.55572003 | DDX20 | Low |
| TCGA-DD-A4NF-01A-11R-A27V-07 | 1 | 31.4 | 2.55639834 | DDX20 | Low |
| TCGA-ED-A7XP-01A-11R-A352-07 | 1 | 13.3333333 | 2.57077309 | DDX20 | Low |
| TCGA-UB-A7ME-01A-11R-A33J-07 | 1 | 16.2 | 2.57194833 | DDX20 | Low |
| TCGA-DD-AACX-01A-11R-A41C-07 | 1 | 5.66666667 | 2.57247545 | DDX20 | Low |
| TCGA-DD-AAVV-01A-11R-A41C-07 | 1 | 81.8333333 | 2.58469365 | DDX20 | Low |
| TCGA-MI-A75I-01A-11R-A32O-07 | 1 | 21 | 2.58624378 | DDX20 | Low |
| TCGA-FV-A3R2-01A-11R-A22L-07 | 2 | 6.46666667 | 2.5880992 | DDX20 | Low |
| TCGA-5C-A9VG-01A-11R-A37K-07 | 1 | 10.9333333 | 2.59029961 | DDX20 | Low |
| TCGA-G3-A5SL-01A-11R-A27V-07 | 1 | 20.7 | 2.60504863 | DDX20 | Low |
| TCGA-BC-4072-01B-11R-A155-07 | 2 | 49.6666667 | 2.61012056 | DDX20 | Low |
| TCGA-G3-AAV1-01A-11R-A38B-07 | 2 | 11.9666667 | 2.61030846 | DDX20 | Low |
| TCGA-CC-A7IG-01A-11R-A33J-07 | 2 | 9.96666667 | 2.6106817 | DDX20 | Low |
| TCGA-ED-A66X-01A-11R-A311-07 | 1 | 13.5333333 | 2.61279732 | DDX20 | Low |
| TCGA-DD-AAVS-01A-11R-A41C-07 | 1 | 60.7666667 | 2.61489089 | DDX20 | Low |
| TCGA-DD-AACA-01A-11R-A41C-07 | 1 | 76.7 | 2.63097113 | DDX20 | Low |
| TCGA-ED-A7PX-01A-51R-A352-07 | 1 | 0.2 | 2.63267212 | DDX20 | Low |
| TCGA-DD-A1EA-01A-11R-A131-07 | 1 | 80.5 | 2.64467767 | DDX20 | High |
| TCGA-FV-A3I1-01A-11R-A22L-07 | 2 | 8.23333333 | 2.64472458 | DDX20 | High |
| TCGA-BD-A3EP-01A-11R-A22L-07 | 1 | 13.6333333 | 2.66133284 | DDX20 | High |
| TCGA-UB-A7MC-01A-11R-A33R-07 | 1 | 16.6666667 | 2.67891983 | DDX20 | High |
| TCGA-DD-A1EL-01A-11R-A155-07 | 2 | 13.8333333 | 2.68363746 | DDX20 | High |
| TCGA-EP-A3RK-01A-11R-A22L-07 | 1 | 12.1 | 2.68564838 | DDX20 | High |
| TCGA-DD-A1EC-01A-21R-A131-07 | 1 | 20.0666667 | 2.68800547 | DDX20 | High |
| TCGA-CC-A9FV-01A-11R-A37K-07 | 1 | 0 | 2.69757708 | DDX20 | High |
| TCGA-BW-A5NP-01A-11R-A27V-07 | 1 | 0 | 2.70028212 | DDX20 | High |
| TCGA-CC-5262-01A-01R-A131-07 | 2 | 3.43333333 | 2.70812766 | DDX20 | High |
| TCGA-DD-AAD8-01A-11R-A41C-07 | 1 | 40.6333333 | 2.71141609 | DDX20 | High |
| TCGA-DD-A4NR-01A-11R-A311-07 | 2 | 0.3 | 2.71176181 | DDX20 | High |
| TCGA-DD-AACA-02A-11R-A41C-07 | 1 | 76.7 | 2.71713751 | DDX20 | High |
| TCGA-DD-AAD1-01A-11R-A41C-07 | 1 | 18.8 | 2.72085691 | DDX20 | High |
| TCGA-DD-A39Y-01A-11R-A213-07 | 2 | 5.7 | 2.72341779 | DDX20 | High |
| TCGA-DD-AACZ-01A-11R-A41C-07 | 2 | 5.7 | 2.72708378 | DDX20 | High |
| TCGA-DD-A11A-01A-11R-A131-07 | 1 | 2.63333333 | 2.73114929 | DDX20 | High |
| TCGA-BC-A3KG-01A-11R-A213-07 | 1 | 22.6666667 | 2.73254636 | DDX20 | High |
| TCGA-ED-A97K-01A-21R-A38B-07 | 1 | 0.2 | 2.73686887 | DDX20 | High |
| TCGA-DD-AADV-01A-11R-A39D-07 | 1 | 19.1333333 | 2.73965352 | DDX20 | High |
| TCGA-XR-A8TG-01A-11R-A36F-07 | 1 | 29.9333333 | 2.74960049 | DDX20 | High |
| TCGA-G3-A5SJ-01A-11R-A27V-07 | 1 | 23.2666667 | 2.7550386 | DDX20 | High |
| TCGA-CC-5258-01A-01R-A131-07 | 2 | 4.3 | 2.75579361 | DDX20 | High |
| TCGA-DD-A39W-01A-11R-A213-07 | 2 | 27.5666667 | 2.75617396 | DDX20 | High |
| TCGA-2Y-A9GY-01A-11R-A38B-07 | 2 | 25.2333333 | 2.75964706 | DDX20 | High |
| TCGA-BC-A10U-01A-11R-A131-07 | 2 | 27.9 | 2.76122561 | DDX20 | High |
| TCGA-CC-A8HV-01A-11R-A36F-07 | 2 | 9.3 | 2.76586315 | DDX20 | High |
| TCGA-DD-AAW3-01A-11R-A41C-07 | 1 | 54.4333333 | 2.76687975 | DDX20 | High |
| TCGA-GJ-A9DB-01A-11R-A37K-07 | 2 | 2.23333333 | 2.76869336 | DDX20 | High |
| TCGA-FV-A23B-01A-11R-A16W-07 | 2 | 61.7333333 | 2.76917184 | DDX20 | High |
| TCGA-G3-A25U-01A-11R-A16W-07 | 1 | 54.5333333 | 2.7720877 | DDX20 | High |
| TCGA-DD-AAW0-01A-11R-A41C-07 | 1 | 67.1666667 | 2.77846896 | DDX20 | High |
| TCGA-DD-AADP-01A-11R-A39D-07 | 1 | 15.2666667 | 2.78459168 | DDX20 | High |
| TCGA-DD-AADI-01A-11R-A41C-07 | 1 | 36.1666667 | 2.793264 | DDX20 | High |
| TCGA-G3-A3CK-01A-11R-A213-07 | 1 | 19.5 | 2.80130327 | DDX20 | High |
| TCGA-DD-AAC8-01A-11R-A41C-07 | 2 | 0.53333333 | 2.8177149 | DDX20 | High |
| TCGA-DD-AAD0-01A-11R-A41C-07 | 1 | 4.56666667 | 2.83649301 | DDX20 | High |
| TCGA-K7-AAU7-01A-11R-A38B-07 | 1 | 11.9666667 | 2.8373272 | DDX20 | High |
| TCGA-BC-A10Y-01A-11R-A131-07 | 2 | 23.7 | 2.84101124 | DDX20 | High |
| TCGA-DD-A4NS-01A-11R-A311-07 | 2 | 81.8666667 | 2.84680985 | DDX20 | High |
| TCGA-DD-A4NJ-01A-11R-A27V-07 | 1 | 30.9333333 | 2.85441627 | DDX20 | High |
| TCGA-G3-A6UC-01A-21R-A33J-07 | 1 | 22.3666667 | 2.8562461 | DDX20 | High |
| TCGA-4R-AA8I-01A-11R-A38B-07 | 2 | 8.73333333 | 2.86410933 | DDX20 | High |
| TCGA-DD-AACL-01A-11R-A41C-07 | 2 | 3.56666667 | 2.87034405 | DDX20 | High |
| TCGA-DD-AADY-01A-11R-A41C-07 | 1 | 18.5 | 2.87111977 | DDX20 | High |
| TCGA-G3-AAV4-01A-11R-A38B-07 | 2 | 0.9 | 2.87113002 | DDX20 | High |
| TCGA-BC-A217-01A-11R-A155-07 | 2 | 46.5666667 | 2.87252233 | DDX20 | High |
| TCGA-ZS-A9CF-02A-11R-A38B-07 | 1 | 80.4 | 2.8731392 | DDX20 | High |
| TCGA-CC-A8HU-01A-11R-A36F-07 | 2 | 11.4666667 | 2.87405805 | DDX20 | High |
| TCGA-XR-A8TE-01A-11R-A36F-07 | 1 | 30.8333333 | 2.8786682 | DDX20 | High |
| TCGA-XR-A8TF-01A-11R-A36F-07 | 2 | 23.1 | 2.89787789 | DDX20 | High |
| TCGA-DD-A73E-01A-12R-A32O-07 | 1 | 1.46666667 | 2.92392928 | DDX20 | High |
| TCGA-BC-A3KF-01A-11R-A213-07 | 1 | 0.26666667 | 2.92423457 | DDX20 | High |
| TCGA-EP-A12J-01A-11R-A131-07 | 1 | 19 | 2.94675683 | DDX20 | High |
| TCGA-DD-AACB-01A-11R-A41C-07 | 1 | 77.4666667 | 2.94854005 | DDX20 | High |
| TCGA-ZP-A9CV-01A-11R-A38B-07 | 2 | 36.2666667 | 2.95214018 | DDX20 | High |
| TCGA-2Y-A9HA-01A-11R-A39D-07 | 2 | 1.2 | 2.96494192 | DDX20 | High |
| TCGA-UB-A7MA-01A-11R-A33R-07 | 1 | 28.2666667 | 2.96887744 | DDX20 | High |
| TCGA-DD-A1EF-01A-11R-A131-07 | 2 | 13.1333333 | 2.96928521 | DDX20 | High |
| TCGA-DD-A4NA-01A-11R-A266-07 | 1 | 33.6 | 2.99268564 | DDX20 | High |
| TCGA-ZP-A9CZ-01A-11R-A38B-07 | 1 | 23.5333333 | 2.99949464 | DDX20 | High |
| TCGA-CC-A8HT-01A-11R-A36F-07 | 2 | 4.66666667 | 3.00256147 | DDX20 | High |
| TCGA-BC-A216-01A-11R-A155-07 | 1 | 45.0333333 | 3.01102086 | DDX20 | High |
| TCGA-UB-AA0U-01A-11R-A38B-07 | 1 | 10.9 | 3.0261909 | DDX20 | High |
| TCGA-RC-A6M3-01A-11R-A32O-07 | 1 | 0 | 3.03016212 | DDX20 | High |
| TCGA-DD-AACK-01A-11R-A41C-07 | 1 | 0.3 | 3.03743157 | DDX20 | High |
| TCGA-LG-A6GG-01A-11R-A311-07 | 1 | 12.9 | 3.04288179 | DDX20 | High |
| TCGA-DD-AACV-01A-11R-A41C-07 | 1 | 51.0333333 | 3.05366798 | DDX20 | High |
| TCGA-QA-A7B7-01A-11R-A32O-07 | 1 | 3.13333333 | 3.06770897 | DDX20 | High |
| TCGA-YA-A8S7-01A-11R-A37K-07 | 2 | 13.7333333 | 3.07279355 | DDX20 | High |
| TCGA-DD-AADB-01A-11R-A41C-07 | 1 | 41.4 | 3.10607076 | DDX20 | High |
| TCGA-CC-A5UD-01A-11R-A28V-07 | 2 | 10.1333333 | 3.10762008 | DDX20 | High |
| TCGA-CC-5264-01A-01R-A131-07 | 2 | 3.4 | 3.11924293 | DDX20 | High |
| TCGA-CC-A3MA-01A-11R-A213-07 | 2 | 10.1 | 3.14742143 | DDX20 | High |
| TCGA-CC-A3M9-01A-11R-A213-07 | 2 | 10 | 3.16947509 | DDX20 | High |
| TCGA-2Y-A9GS-01A-12R-A38B-07 | 2 | 24.1333333 | 3.18526882 | DDX20 | High |
| TCGA-DD-AA3A-01A-11R-A37K-07 | 2 | 13.6666667 | 3.19443027 | DDX20 | High |
| TCGA-DD-A1EE-01A-11R-A131-07 | 2 | 11.6333333 | 3.19885585 | DDX20 | High |
| TCGA-DD-AADC-01A-11R-A41C-07 | 2 | 14.1666667 | 3.20100255 | DDX20 | High |
| TCGA-DD-AACP-01A-11R-A41C-07 | 1 | 13.8333333 | 3.20593796 | DDX20 | High |
| TCGA-BC-4073-01B-02R-A131-07 | 1 | 28.3 | 3.20730323 | DDX20 | High |
| TCGA-DD-A1EJ-01A-11R-A155-07 | 2 | 33.5 | 3.2304366 | DDX20 | High |
| TCGA-FV-A4ZQ-01A-11R-A266-07 | 1 | 0.4 | 3.23235495 | DDX20 | High |
| TCGA-CC-A9FU-01A-11R-A37K-07 | 1 | 0 | 3.23278842 | DDX20 | High |
| TCGA-ED-A82E-01A-11R-A352-07 | 1 | 13.6 | 3.26699248 | DDX20 | High |
| TCGA-DD-AACH-01A-11R-A41C-07 | 2 | 6.5 | 3.26819925 | DDX20 | High |
| TCGA-CC-5263-01A-01R-A131-07 | 2 | 4.3 | 3.30979823 | DDX20 | High |
| TCGA-5R-AA1C-01A-11R-A41C-07 | 1 | 17.3333333 | 3.31835287 | DDX20 | High |
| TCGA-CC-5261-01A-01R-A131-07 | 2 | 3.23333333 | 3.31928826 | DDX20 | High |
| TCGA-CC-5260-01A-01R-A131-07 | 2 | 2.9 | 3.33327431 | DDX20 | High |
| TCGA-DD-AAVZ-01A-11R-A41C-07 | 1 | 63.3333333 | 3.33351095 | DDX20 | High |
| TCGA-CC-A5UE-01A-11R-A28V-07 | 2 | 9.06666667 | 3.39407369 | DDX20 | High |
| TCGA-G3-AAV7-01A-11R-A38B-07 | 1 | 12.0333333 | 3.3942764 | DDX20 | High |
| TCGA-UB-A7MB-01A-11R-A33R-07 | 1 | 20.0333333 | 3.39767131 | DDX20 | High |
| TCGA-ED-A5KG-01A-11R-A27V-07 | 1 | 28.4666667 | 3.41275128 | DDX20 | High |
| TCGA-CC-A7IK-01A-12R-A33R-07 | 2 | 8.73333333 | 3.45567856 | DDX20 | High |
| TCGA-ED-A8O5-01A-11R-A36F-07 | 1 | 13.5333333 | 3.46618197 | DDX20 | High |
| TCGA-DD-AADD-01A-11R-A41C-07 | 1 | 41.0333333 | 3.47561612 | DDX20 | High |
| TCGA-BC-A8YO-01A-11R-A37K-07 | 1 | 18.7333333 | 3.50942013 | DDX20 | High |
| TCGA-2Y-A9H2-01A-12R-A38B-07 | 1 | 57.7 | 3.52860895 | DDX20 | High |
| TCGA-DD-A114-01A-11R-A131-07 | 2 | 38.3 | 3.5485037 | DDX20 | High |
| TCGA-CC-A7IJ-01A-11R-A33R-07 | 1 | 12.7333333 | 3.56201079 | DDX20 | High |
| TCGA-CC-A8HS-01A-11R-A36F-07 | 2 | 10 | 3.56658175 | DDX20 | High |
| TCGA-CC-A1HT-01A-11R-A131-07 | 2 | 3.36666667 | 3.60334041 | DDX20 | High |
| TCGA-CC-A7II-01A-11R-A33J-07 | 1 | 13.3 | 3.62753035 | DDX20 | High |
| TCGA-BC-A112-01A-11R-A131-07 | 2 | 5.1 | 3.64017422 | DDX20 | High |
| TCGA-ZP-A9D2-01A-11R-A38B-07 | 2 | 25.5 | 3.65838554 | DDX20 | High |
| TCGA-BW-A5NQ-01A-11R-A27V-07 | 1 | 0 | 3.66898541 | DDX20 | High |
| TCGA-G3-A7M9-01A-23R-A352-07 | 2 | 1.86666667 | 4.00236897 | DDX20 | High |
| TCGA-G3-A7M6-01A-11R-A33R-07 | 1 | 21.0666667 | 4.02958059 | DDX20 | High |
| TCGA-BC-A10W-01A-11R-A131-07 | 2 | 3.03333333 | 4.9422319 | DDX20 | High |

**Supplement. 2 PFI optimal cutoff value**

| sample_id | event | time | expr | gene | group |
| --- | --- | --- | --- | --- | --- |
| TCGA-DD-AADJ-01A-11R-A41C-07 | 1 | 35.5333333 | 1.06421248 | DDX20 | Low |
| TCGA-DD-A3A2-01A-11R-A213-07 | 1 | 71.0333333 | 1.16526421 | DDX20 | Low |
| TCGA-ES-A2HS-01A-11R-A180-07 | 1 | 22.9333333 | 1.17071244 | DDX20 | Low |
| TCGA-FV-A4ZP-01A-12R-A266-07 | 1 | 82.8666667 | 1.25593027 | DDX20 | Low |
| TCGA-DD-A3A7-01A-11R-A22L-07 | 2 | 4.8 | 1.26859986 | DDX20 | Low |
| TCGA-2Y-A9H1-01A-11R-A38B-07 | 2 | 10.7 | 1.27912311 | DDX20 | Low |
| TCGA-DD-AAEB-01A-11R-A41C-07 | 1 | 15.9333333 | 1.28265443 | DDX20 | Low |
| TCGA-G3-AAV0-01A-11R-A37K-07 | 1 | 15.8666667 | 1.29509613 | DDX20 | Low |
| TCGA-DD-AADS-01A-11R-A41C-07 | 1 | 15.8 | 1.31969782 | DDX20 | Low |
| TCGA-2Y-A9H3-01A-11R-A38B-07 | 2 | 0.73333333 | 1.33416283 | DDX20 | Low |
| TCGA-CC-5259-01A-31R-A213-07 | 2 | 6 | 1.35141987 | DDX20 | Low |
| TCGA-DD-A3A9-01A-11R-A266-07 | 2 | 9.63333333 | 1.3974612 | DDX20 | Low |
| TCGA-DD-AADO-01A-11R-A41C-07 | 1 | 15.1 | 1.40066352 | DDX20 | Low |
| TCGA-ES-A2HT-01A-12R-A180-07 | 1 | 14.6 | 1.43357022 | DDX20 | Low |
| TCGA-WQ-AB4B-01A-11R-A41C-07 | 2 | 9.1 | 1.45679822 | DDX20 | Low |
| TCGA-RC-A7SH-01A-11R-A38B-07 | 2 | 3.03333333 | 1.46730412 | DDX20 | Low |
| TCGA-2Y-A9H6-01A-11R-A39D-07 | 1 | 11.9 | 1.49686387 | DDX20 | Low |
| TCGA-DD-A3A3-01A-11R-A22L-07 | 1 | 17.8333333 | 1.5023969 | DDX20 | Low |
| TCGA-2Y-A9GU-01A-11R-A38B-07 | 1 | 64.6333333 | 1.51033494 | DDX20 | Low |
| TCGA-DD-A73G-01A-22R-A32O-07 | 1 | 115.933333 | 1.52872998 | DDX20 | Low |
| TCGA-DD-A39X-01A-11R-A213-07 | 2 | 34.4 | 1.5473309 | DDX20 | Low |
| TCGA-NI-A4U2-01A-11R-A28V-07 | 2 | 3.53333333 | 1.5491896 | DDX20 | Low |
| TCGA-DD-A11C-01A-11R-A131-07 | 1 | 22.0666667 | 1.55170997 | DDX20 | Low |
| TCGA-BC-A10R-01A-11R-A131-07 | 2 | 6.63333333 | 1.55641699 | DDX20 | Low |
| TCGA-DD-AAE1-01A-11R-A41C-07 | 1 | 18.4 | 1.56164519 | DDX20 | Low |
| TCGA-DD-AADU-01A-11R-A41C-07 | 1 | 18.4666667 | 1.58463853 | DDX20 | Low |
| TCGA-NI-A8LF-01A-11R-A36F-07 | 1 | 26.6333333 | 1.60386912 | DDX20 | Low |
| TCGA-DD-AAC9-01A-11R-A41C-07 | 1 | 11.5666667 | 1.61501197 | DDX20 | Low |
| TCGA-DD-A4NG-01A-11R-A27V-07 | 2 | 14.3333333 | 1.6262543 | DDX20 | Low |
| TCGA-DD-A119-01A-11R-A131-07 | 1 | 7.43333333 | 1.63397767 | DDX20 | Low |
| TCGA-DD-A11B-01A-11R-A131-07 | 1 | 0.46666667 | 1.6353811 | DDX20 | Low |
| TCGA-DD-AADN-01A-11R-A41C-07 | 1 | 29.9333333 | 1.63830221 | DDX20 | Low |
| TCGA-DD-AADM-01A-11R-A41C-07 | 1 | 0.4 | 1.64586394 | DDX20 | Low |
| TCGA-DD-A1EK-01A-11R-A213-07 | 2 | 5.56666667 | 1.65049789 | DDX20 | Low |
| TCGA-EP-A3JL-01A-11R-A213-07 | 1 | 10.1 | 1.65474362 | DDX20 | Low |
| TCGA-G3-A5SM-01A-12R-A28V-07 | 2 | 8.4 | 1.6669265 | DDX20 | Low |
| TCGA-2Y-A9GT-01A-11R-A38B-07 | 2 | 36.1 | 1.66704031 | DDX20 | Low |
| TCGA-RC-A7SF-01A-11R-A352-07 | 2 | 16.7333333 | 1.67023264 | DDX20 | Low |
| TCGA-WX-AA47-01A-11R-A39D-07 | 2 | 3.43333333 | 1.67117576 | DDX20 | Low |
| TCGA-DD-A11D-01A-11R-A131-07 | 2 | 16.6 | 1.67386501 | DDX20 | Low |
| TCGA-G3-A7M5-01A-11R-A33R-07 | 1 | 14.9 | 1.67885141 | DDX20 | Low |
| TCGA-KR-A7K7-01A-11R-A33J-07 | 2 | 7.96666667 | 1.68311326 | DDX20 | Low |
| TCGA-DD-A4NP-01A-11R-A28V-07 | 2 | 42.8666667 | 1.69338093 | DDX20 | Low |
| TCGA-EP-A2KB-01A-11R-A180-07 | 2 | 5.36666667 | 1.69444014 | DDX20 | Low |
| TCGA-5C-A9VH-01A-11R-A37K-07 | 1 | 10.7333333 | 1.71231545 | DDX20 | Low |
| TCGA-DD-AAE3-01A-11R-A41C-07 | 1 | 18.8666667 | 1.72677035 | DDX20 | Low |
| TCGA-DD-A3A6-01A-11R-A22L-07 | 1 | 108.6 | 1.72700077 | DDX20 | Low |
| TCGA-LG-A9QD-01A-11R-A38B-07 | 1 | 12.2 | 1.72751901 | DDX20 | Low |
| TCGA-G3-A5SK-01A-11R-A27V-07 | 2 | 16.1 | 1.73190712 | DDX20 | Low |
| TCGA-DD-A4NL-01A-11R-A28V-07 | 1 | 57.0333333 | 1.73620405 | DDX20 | Low |
| TCGA-BC-A10S-01A-22R-A131-07 | 2 | 37.6666667 | 1.75346611 | DDX20 | Low |
| TCGA-G3-AAUZ-01A-11R-A38B-07 | 1 | 16 | 1.76058938 | DDX20 | Low |
| TCGA-DD-AACD-01A-11R-A41C-07 | 2 | 5.03333333 | 1.76241874 | DDX20 | Low |
| TCGA-BC-A110-01A-11R-A131-07 | 2 | 68.5666667 | 1.775718 | DDX20 | Low |
| TCGA-RC-A7SB-01A-21R-A352-07 | 1 | 19.6 | 1.78342143 | DDX20 | Low |
| TCGA-DD-A39Z-01A-11R-A213-07 | 1 | 20.0333333 | 1.79499311 | DDX20 | Low |
| TCGA-FV-A3R3-01A-11R-A22L-07 | 1 | 12.2 | 1.80245974 | DDX20 | Low |
| TCGA-G3-AAV6-01A-21R-A37K-07 | 2 | 1.46666667 | 1.80575721 | DDX20 | Low |
| TCGA-DD-AADF-01A-11R-A41C-07 | 1 | 3.83333333 | 1.81358773 | DDX20 | Low |
| TCGA-DD-AADK-01A-11R-A41C-07 | 1 | 34.9666667 | 1.81421643 | DDX20 | Low |
| TCGA-G3-A3CJ-01A-11R-A213-07 | 2 | 9.86666667 | 1.81619652 | DDX20 | Low |
| TCGA-G3-A25Z-01A-11R-A16W-07 | 1 | 21.8333333 | 1.81932796 | DDX20 | Low |
| TCGA-DD-A4NE-01A-11R-A27V-07 | 2 | 6.33333333 | 1.82307083 | DDX20 | Low |
| TCGA-K7-A6G5-01A-11R-A311-07 | 1 | 17.0666667 | 1.82630909 | DDX20 | Low |
| TCGA-G3-A3CI-01A-11R-A213-07 | 1 | 6 | 1.83228142 | DDX20 | Low |
| TCGA-CC-A123-01A-11R-A131-07 | 2 | 4.83333333 | 1.8325137 | DDX20 | Low |
| TCGA-DD-AADA-01A-11R-A41C-07 | 1 | 41.1 | 1.8352515 | DDX20 | Low |
| TCGA-DD-AAE4-01A-11R-A41C-07 | 2 | 3.7 | 1.84067771 | DDX20 | Low |
| TCGA-DD-A73A-01A-12R-A32O-07 | 1 | 24.2666667 | 1.84249609 | DDX20 | Low |
| TCGA-HP-A5MZ-01A-21R-A27V-07 | 1 | 3.03333333 | 1.8425587 | DDX20 | Low |
| TCGA-2Y-A9GW-01A-11R-A38B-07 | 2 | 40.9666667 | 1.84391737 | DDX20 | Low |
| TCGA-DD-A4NI-01A-11R-A27V-07 | 1 | 27.2 | 1.86146197 | DDX20 | Low |
| TCGA-WJ-A86L-01A-12R-A39D-07 | 1 | 11.5 | 1.87332093 | DDX20 | Low |
| TCGA-DD-AAE7-01A-11R-A41C-07 | 1 | 21.4666667 | 1.88061718 | DDX20 | Low |
| TCGA-DD-AAVW-01A-11R-A41C-07 | 1 | 77.2333333 | 1.88507653 | DDX20 | Low |
| TCGA-DD-AACI-01A-11R-A41C-07 | 2 | 47.7333333 | 1.8859475 | DDX20 | Low |
| TCGA-EP-A2KC-01A-11R-A213-07 | 1 | 0.63333333 | 1.88937676 | DDX20 | Low |
| TCGA-DD-A3A8-01A-11R-A22L-07 | 1 | 0.36666667 | 1.89197789 | DDX20 | Low |
| TCGA-DD-AACE-01A-11R-A41C-07 | 2 | 42.6333333 | 1.89855749 | DDX20 | Low |
| TCGA-KR-A7K2-01A-12R-A33R-07 | 1 | 27.6333333 | 1.89881097 | DDX20 | Low |
| TCGA-DD-A39V-01A-11R-A213-07 | 2 | 15.1666667 | 1.90960075 | DDX20 | Low |
| TCGA-G3-A3CH-01A-11R-A22L-07 | 2 | 3.86666667 | 1.91064494 | DDX20 | Low |
| TCGA-CC-A7IF-01A-11R-A33J-07 | 2 | 8.46666667 | 1.91110577 | DDX20 | Low |
| TCGA-MI-A75C-01A-11R-A32O-07 | 1 | 9.7 | 1.91900343 | DDX20 | Low |
| TCGA-BC-A69H-01A-11R-A311-07 | 1 | 14.8 | 1.92523076 | DDX20 | Low |
| TCGA-K7-A5RF-01A-11R-A28V-07 | 2 | 6.83333333 | 1.92642247 | DDX20 | Low |
| TCGA-BD-A3ER-01A-11R-A213-07 | 2 | 7.53333333 | 1.92819697 | DDX20 | Low |
| TCGA-DD-AAEI-01A-11R-A41C-07 | 2 | 16.3666667 | 1.92919097 | DDX20 | Low |
| TCGA-FV-A495-01A-11R-A266-07 | 1 | 0.03333333 | 1.93166149 | DDX20 | Low |
| TCGA-DD-AACW-01A-11R-A41C-07 | 1 | 47.4666667 | 1.93227997 | DDX20 | Low |
| TCGA-DD-AAVR-01A-11R-A41C-07 | 1 | 83.7666667 | 1.93472901 | DDX20 | Low |
| TCGA-DD-AACT-01A-11R-A41C-07 | 1 | 52.0666667 | 1.94267086 | DDX20 | Low |
| TCGA-ZS-A9CD-01A-11R-A37K-07 | 2 | 12.3666667 | 1.94312951 | DDX20 | Low |
| TCGA-DD-AAEG-01A-11R-A39D-07 | 1 | 23.9666667 | 1.95077057 | DDX20 | Low |
| TCGA-WX-AA46-01A-11R-A39D-07 | 1 | 25.2 | 1.95098088 | DDX20 | Low |
| TCGA-DD-AAE9-01A-11R-A41C-07 | 1 | 24.0666667 | 1.95367449 | DDX20 | Low |
| TCGA-G3-A7M8-01A-11R-A33R-07 | 1 | 14.3333333 | 1.95872743 | DDX20 | Low |
| TCGA-G3-AAV5-01A-11R-A37K-07 | 2 | 3.9 | 1.96055629 | DDX20 | Low |
| TCGA-FV-A3I0-01A-11R-A22L-07 | 2 | 5.3 | 1.97020285 | DDX20 | Low |
| TCGA-2Y-A9H4-01A-11R-A38B-07 | 1 | 48.4 | 1.99854291 | DDX20 | Low |
| TCGA-DD-AAVQ-01A-11R-A41C-07 | 2 | 50.3 | 2.00592941 | DDX20 | Low |
| TCGA-DD-A1ED-01A-11R-A155-07 | 1 | 76.7 | 2.00717668 | DDX20 | Low |
| TCGA-5R-AAAM-01A-12R-A41C-07 | 1 | 1.53333333 | 2.00737971 | DDX20 | Low |
| TCGA-DD-AAD2-01A-11R-A41C-07 | 1 | 21.9333333 | 2.01474322 | DDX20 | Low |
| TCGA-DD-A115-01A-11R-A131-07 | 2 | 15.6333333 | 2.01624575 | DDX20 | Low |
| TCGA-MI-A75G-01A-11R-A32O-07 | 1 | 23.2666667 | 2.01787599 | DDX20 | Low |
| TCGA-DD-A3A4-01A-11R-A22L-07 | 2 | 2.9 | 2.03754666 | DDX20 | Low |
| TCGA-DD-AACY-01A-11R-A41C-07 | 2 | 25.8666667 | 2.03755383 | DDX20 | Low |
| TCGA-RC-A6M6-01A-11R-A32O-07 | 1 | 0.3 | 2.04509128 | DDX20 | Low |
| TCGA-MR-A8JO-01A-12R-A36F-07 | 1 | 11 | 2.04534158 | DDX20 | Low |
| TCGA-DD-A118-01A-11R-A131-07 | 2 | 21.9333333 | 2.04763012 | DDX20 | Low |
| TCGA-BD-A2L6-01A-11R-A213-07 | 2 | 13.8333333 | 2.04980543 | DDX20 | Low |
| TCGA-MR-A520-01A-11R-A266-07 | 1 | 7.63333333 | 2.05514707 | DDX20 | Low |
| TCGA-CC-A3MB-01A-11R-A213-07 | 2 | 8.66666667 | 2.05762217 | DDX20 | Low |
| TCGA-5C-AAPD-01A-21R-A39D-07 | 1 | 0.66666667 | 2.05811555 | DDX20 | Low |
| TCGA-O8-A75V-01A-11R-A32O-07 | 1 | 17.9333333 | 2.06548958 | DDX20 | Low |
| TCGA-DD-A113-01A-11R-A131-07 | 2 | 55.8666667 | 2.06874972 | DDX20 | Low |
| TCGA-DD-AAEA-01A-11R-A41C-07 | 1 | 19.1666667 | 2.07086742 | DDX20 | Low |
| TCGA-DD-A116-01A-11R-A131-07 | 2 | 27.6 | 2.07171149 | DDX20 | Low |
| TCGA-BC-A10X-01A-11R-A131-07 | 1 | 25.6666667 | 2.07414015 | DDX20 | Low |
| TCGA-ED-A7PZ-01A-11R-A33R-07 | 1 | 0.2 | 2.07757736 | DDX20 | Low |
| TCGA-BC-A69I-01A-11R-A311-07 | 1 | 12.9 | 2.07805984 | DDX20 | Low |
| TCGA-ZS-A9CG-01A-11R-A37K-07 | 1 | 11.3666667 | 2.07959572 | DDX20 | Low |
| TCGA-DD-AAW1-01A-11R-A41C-07 | 1 | 66.3 | 2.0867939 | DDX20 | Low |
| TCGA-MI-A75E-01A-11R-A32O-07 | 1 | 16.9 | 2.09083744 | DDX20 | Low |
| TCGA-G3-A7M7-01A-12R-A352-07 | 1 | 12.0333333 | 2.0971436 | DDX20 | Low |
| TCGA-DD-A4NV-01A-11R-A311-07 | 1 | 79.9333333 | 2.09864647 | DDX20 | Low |
| TCGA-DD-AAEK-01A-11R-A41C-07 | 1 | 35.5666667 | 2.10325697 | DDX20 | High |
| TCGA-CC-A3MC-01A-11R-A22L-07 | 2 | 9.9 | 2.10581808 | DDX20 | High |
| TCGA-2Y-A9H5-01A-11R-A38B-07 | 2 | 8.6 | 2.12304557 | DDX20 | High |
| TCGA-DD-AACA-02B-11R-A41C-07 | 2 | 25.8666667 | 2.12679422 | DDX20 | High |
| TCGA-XR-A8TC-01A-11R-A36F-07 | 1 | 44.6333333 | 2.13003238 | DDX20 | High |
| TCGA-DD-AACG-01A-11R-A41C-07 | 2 | 4.16666667 | 2.1312006 | DDX20 | High |
| TCGA-BC-A10T-01A-11R-A131-07 | 2 | 19.9333333 | 2.13162481 | DDX20 | High |
| TCGA-DD-A1EI-01A-11R-A131-07 | 1 | 6.1 | 2.13198571 | DDX20 | High |
| TCGA-DD-AAD6-01A-11R-A41C-07 | 2 | 1.66666667 | 2.13510422 | DDX20 | High |
| TCGA-DD-A3A5-01A-11R-A22L-07 | 2 | 2.8 | 2.1355512 | DDX20 | High |
| TCGA-DD-AACO-01A-11R-A41C-07 | 2 | 15.9666667 | 2.13725626 | DDX20 | High |
| TCGA-DD-AACU-01A-11R-A41C-07 | 1 | 52.2333333 | 2.13767705 | DDX20 | High |
| TCGA-DD-AAE0-01A-11R-A41C-07 | 1 | 18.5 | 2.13935378 | DDX20 | High |
| TCGA-G3-A25V-01A-11R-A16W-07 | 2 | 16.3 | 2.14745332 | DDX20 | High |
| TCGA-DD-AAE6-01A-11R-A41C-07 | 2 | 3.5 | 2.15565003 | DDX20 | High |
| TCGA-XR-A8TD-01A-12R-A39D-07 | 1 | 34.3333333 | 2.15906288 | DDX20 | High |
| TCGA-DD-AAD3-01A-11R-A41C-07 | 1 | 43.1666667 | 2.16604645 | DDX20 | High |
| TCGA-EP-A2KA-01A-11R-A180-07 | 2 | 9.76666667 | 2.16935578 | DDX20 | High |
| TCGA-DD-A1EH-01A-11R-A131-07 | 2 | 4 | 2.17955971 | DDX20 | High |
| TCGA-RC-A7SK-01A-11R-A352-07 | 2 | 10.4 | 2.19200955 | DDX20 | High |
| TCGA-RC-A7S9-01A-11R-A33R-07 | 1 | 21.3333333 | 2.19223796 | DDX20 | High |
| TCGA-DD-AACQ-01A-11R-A41C-07 | 2 | 3.5 | 2.19242032 | DDX20 | High |
| TCGA-ZP-A9CY-01A-11R-A38B-07 | 1 | 26.0666667 | 2.20261411 | DDX20 | High |
| TCGA-BC-A5W4-01A-11R-A28V-07 | 2 | 12.8 | 2.20780244 | DDX20 | High |
| TCGA-DD-A4NB-01A-12R-A266-07 | 2 | 30.4 | 2.20842151 | DDX20 | High |
| TCGA-DD-A3A1-01A-11R-A213-07 | 1 | 7.76666667 | 2.21299134 | DDX20 | High |
| TCGA-G3-A25X-01A-11R-A16W-07 | 1 | 59.3 | 2.21446914 | DDX20 | High |
| TCGA-GJ-A6C0-01A-12R-A311-07 | 1 | 1.03333333 | 2.21803674 | DDX20 | High |
| TCGA-MI-A75H-01A-11R-A32O-07 | 2 | 10.0333333 | 2.21857284 | DDX20 | High |
| TCGA-G3-A3CG-01A-11R-A213-07 | 2 | 10.9666667 | 2.22601874 | DDX20 | High |
| TCGA-ED-A7PY-01A-11R-A33R-07 | 1 | 13 | 2.22685311 | DDX20 | High |
| TCGA-2Y-A9H9-01A-21R-A39D-07 | 2 | 1.96666667 | 2.23072292 | DDX20 | High |
| TCGA-WQ-A9G7-01A-11R-A37K-07 | 1 | 1 | 2.23117955 | DDX20 | High |
| TCGA-DD-AAW2-01A-11R-A41C-07 | 2 | 13.0333333 | 2.23619973 | DDX20 | High |
| TCGA-DD-AAE2-01A-11R-A41C-07 | 1 | 21.2666667 | 2.2368352 | DDX20 | High |
| TCGA-ZP-A9D1-01A-11R-A38B-07 | 1 | 0.7 | 2.25444745 | DDX20 | High |
| TCGA-DD-AACC-01A-11R-A41C-07 | 2 | 17.9 | 2.25558876 | DDX20 | High |
| TCGA-GJ-A3OU-01A-31R-A38B-07 | 2 | 29.3 | 2.25846097 | DDX20 | High |
| TCGA-UB-A7MF-01A-11R-A33J-07 | 2 | 4.43333333 | 2.25945197 | DDX20 | High |
| TCGA-KR-A7K8-01A-11R-A33J-07 | 1 | 30.2 | 2.25982063 | DDX20 | High |
| TCGA-DD-A73F-01A-11R-A32O-07 | 1 | 36.1666667 | 2.26182069 | DDX20 | High |
| TCGA-DD-A4NK-01A-11R-A28V-07 | 2 | 2.96666667 | 2.26976828 | DDX20 | High |
| TCGA-G3-A25S-01A-11R-A16W-07 | 2 | 1.23333333 | 2.27031298 | DDX20 | High |
| TCGA-DD-AADQ-01A-11R-A41C-07 | 1 | 14.5333333 | 2.27348195 | DDX20 | High |
| TCGA-DD-AAEH-01A-11R-A41C-07 | 2 | 21.4666667 | 2.27445258 | DDX20 | High |
| TCGA-EP-A26S-01A-11R-A16W-07 | 1 | 20.2666667 | 2.27479464 | DDX20 | High |
| TCGA-ZP-A9D4-01A-11R-A37K-07 | 1 | 13.1666667 | 2.28266078 | DDX20 | High |
| TCGA-UB-A7MD-01A-12R-A352-07 | 1 | 1.73333333 | 2.29409094 | DDX20 | High |
| TCGA-G3-A25T-01A-11R-A16W-07 | 2 | 15.0666667 | 2.29638197 | DDX20 | High |
| TCGA-T1-A6J8-01A-11R-A32O-07 | 1 | 0.76666667 | 2.29783041 | DDX20 | High |
| TCGA-ZS-A9CF-01A-11R-A38B-07 | 2 | 21.2 | 2.29894047 | DDX20 | High |
| TCGA-G3-AAV2-01A-11R-A37K-07 | 1 | 12.4 | 2.30842601 | DDX20 | High |
| TCGA-DD-A4NH-01A-11R-A27V-07 | 2 | 5.36666667 | 2.30906933 | DDX20 | High |
| TCGA-FV-A496-01A-11R-A266-07 | 1 | 0.33333333 | 2.31474076 | DDX20 | High |
| TCGA-UB-AA0V-01A-11R-A38B-07 | 1 | 10.4666667 | 2.31963818 | DDX20 | High |
| TCGA-DD-AADG-01A-11R-A41C-07 | 2 | 9 | 2.32085513 | DDX20 | High |
| TCGA-5R-AA1D-01A-11R-A38B-07 | 1 | 14.9666667 | 2.32095323 | DDX20 | High |
| TCGA-BC-A10Q-01A-11R-A131-07 | 2 | 11.3333333 | 2.32704767 | DDX20 | High |
| TCGA-ZS-A9CE-01A-11R-A37K-07 | 2 | 29.7333333 | 2.33780245 | DDX20 | High |
| TCGA-DD-AAVU-01A-11R-A41C-07 | 2 | 54.3333333 | 2.34320727 | DDX20 | High |
| TCGA-G3-AAV3-01A-11R-A37K-07 | 1 | 13.7333333 | 2.34369174 | DDX20 | High |
| TCGA-RC-A6M5-01A-11R-A32O-07 | 1 | 0.5 | 2.34605602 | DDX20 | High |
| TCGA-2Y-A9GZ-01A-11R-A39D-07 | 2 | 23.9666667 | 2.35638238 | DDX20 | High |
| TCGA-CC-A7IE-01A-21R-A38B-07 | 2 | 5.63333333 | 2.35910521 | DDX20 | High |
| TCGA-KR-A7K0-01A-12R-A33R-07 | 1 | 2.16666667 | 2.36314355 | DDX20 | High |
| TCGA-DD-AAVX-01A-11R-A41C-07 | 2 | 13.1 | 2.36368313 | DDX20 | High |
| TCGA-ZP-A9D0-01A-11R-A37K-07 | 1 | 36.3666667 | 2.3669836 | DDX20 | High |
| TCGA-DD-AACN-01A-11R-A41C-07 | 2 | 11.9 | 2.36741985 | DDX20 | High |
| TCGA-2Y-A9H8-01A-11R-A39D-07 | 2 | 13.2666667 | 2.37191745 | DDX20 | High |
| TCGA-CC-A9FW-01A-11R-A37K-07 | 2 | 5.1 | 2.38038362 | DDX20 | High |
| TCGA-DD-A1EG-01A-11R-A213-07 | 2 | 29.1666667 | 2.38077114 | DDX20 | High |
| TCGA-CC-A9FS-01A-11R-A37K-07 | 2 | 3.63333333 | 2.38198901 | DDX20 | High |
| TCGA-DD-AACF-01A-11R-A41C-07 | 2 | 3.96666667 | 2.38768645 | DDX20 | High |
| TCGA-DD-AAED-01A-12R-A41C-07 | 2 | 6.6 | 2.39434918 | DDX20 | High |
| TCGA-HP-A5N0-01A-11R-A28V-07 | 2 | 5.96666667 | 2.39688455 | DDX20 | High |
| TCGA-RC-A6M4-01A-11R-A32O-07 | 1 | 0.73333333 | 2.39808537 | DDX20 | High |
| TCGA-ED-A627-01A-12R-A311-07 | 1 | 14.1 | 2.40010195 | DDX20 | High |
| TCGA-ED-A4XI-01A-11R-A266-07 | 1 | 27.3 | 2.40253189 | DDX20 | High |
| TCGA-BC-A10Z-01A-11R-A131-07 | 1 | 1.13333333 | 2.41348937 | DDX20 | High |
| TCGA-DD-A73B-01A-12R-A32O-07 | 2 | 8 | 2.41876534 | DDX20 | High |
| TCGA-RG-A7D4-01A-12R-A33R-07 | 1 | 36.6 | 2.42090174 | DDX20 | High |
| TCGA-CC-A5UC-01A-11R-A28V-07 | 2 | 10.4 | 2.42408522 | DDX20 | High |
| TCGA-3K-AAZ8-01A-12R-A39D-07 | 2 | 5.03333333 | 2.42503516 | DDX20 | High |
| TCGA-G3-A5SI-01A-31R-A27V-07 | 2 | 11.6 | 2.42776141 | DDX20 | High |
| TCGA-DD-A4NQ-01A-21R-A28V-07 | 2 | 4.2 | 2.42900478 | DDX20 | High |
| TCGA-DD-AACJ-01A-11R-A41C-07 | 2 | 3.33333333 | 2.43955117 | DDX20 | High |
| TCGA-ED-A7XO-01A-11R-A352-07 | 2 | 8.73333333 | 2.44054676 | DDX20 | High |
| TCGA-2Y-A9GV-01A-11R-A38B-07 | 2 | 58.1666667 | 2.4446353 | DDX20 | High |
| TCGA-PD-A5DF-01A-11R-A27V-07 | 2 | 21.3 | 2.44699301 | DDX20 | High |
| TCGA-DD-AAVP-01A-11R-A41C-07 | 2 | 30.1 | 2.45585713 | DDX20 | High |
| TCGA-DD-A1EB-01A-11R-A131-07 | 2 | 16.8333333 | 2.45707609 | DDX20 | High |
| TCGA-FV-A2QR-01A-11R-A213-07 | 2 | 18.7 | 2.4657471 | DDX20 | High |
| TCGA-ED-A66Y-01A-11R-A311-07 | 1 | 9.86666667 | 2.46797307 | DDX20 | High |
| TCGA-FV-A2QQ-01A-11R-A22L-07 | 1 | 24.3 | 2.46960615 | DDX20 | High |
| TCGA-2Y-A9H0-01A-11R-A38B-07 | 1 | 122.5 | 2.47385179 | DDX20 | High |
| TCGA-ED-A8O6-01A-11R-A36F-07 | 1 | 1.86666667 | 2.47501249 | DDX20 | High |
| TCGA-DD-AADR-01A-11R-A41C-07 | 2 | 56.6666667 | 2.48074529 | DDX20 | High |
| TCGA-K7-A5RG-01A-11R-A28V-07 | 1 | 17.3 | 2.48327409 | DDX20 | High |
| TCGA-G3-A25Y-01A-11R-A16W-07 | 2 | 11.8333333 | 2.48470336 | DDX20 | High |
| TCGA-LG-A9QC-01A-11R-A37K-07 | 1 | 14.1666667 | 2.48807888 | DDX20 | High |
| TCGA-DD-A4NO-01A-11R-A28V-07 | 2 | 33 | 2.48969148 | DDX20 | High |
| TCGA-CC-A7IH-01A-11R-A33J-07 | 1 | 12.1666667 | 2.49013383 | DDX20 | High |
| TCGA-DD-A4NN-01A-11R-A28V-07 | 2 | 5.53333333 | 2.49027225 | DDX20 | High |
| TCGA-DD-AADW-01A-11R-A39D-07 | 2 | 12.8666667 | 2.49097416 | DDX20 | High |
| TCGA-DD-A73C-01A-12R-A33J-07 | 2 | 18.8666667 | 2.4929807 | DDX20 | High |
| TCGA-BW-A5NO-01A-11R-A27V-07 | 1 | 0.66666667 | 2.49943225 | DDX20 | High |
| TCGA-WX-AA44-01A-11R-A39D-07 | 2 | 8.2 | 2.50203007 | DDX20 | High |
| TCGA-2Y-A9H7-01A-11R-A39D-07 | 2 | 37.2333333 | 2.5041661 | DDX20 | High |
| TCGA-DD-AACS-01A-11R-A41C-07 | 1 | 60.1333333 | 2.50757931 | DDX20 | High |
| TCGA-CC-A7IL-01A-11R-A33R-07 | 2 | 5.96666667 | 2.51076097 | DDX20 | High |
| TCGA-ED-A459-01A-11R-A266-07 | 1 | 30.3333333 | 2.51488025 | DDX20 | High |
| TCGA-DD-AADL-01A-11R-A41C-07 | 1 | 21.2 | 2.51731886 | DDX20 | High |
| TCGA-2Y-A9HB-01A-11R-A39D-07 | 1 | 8.66666667 | 2.5184042 | DDX20 | High |
| TCGA-2V-A95S-01A-11R-A37K-07 | 1 |  | 2.52302893 | DDX20 | High |
| TCGA-DD-A73D-01A-12R-A32O-07 | 2 | 19.7333333 | 2.52527239 | DDX20 | High |
| TCGA-DD-AAD5-01A-11R-A41C-07 | 2 | 4.33333333 | 2.52700514 | DDX20 | High |
| TCGA-DD-AAEE-01A-11R-A41C-07 | 2 | 7.46666667 | 2.53872832 | DDX20 | High |
| TCGA-DD-AAVY-01A-11R-A41C-07 | 1 | 65.6666667 | 2.54041429 | DDX20 | High |
| TCGA-DD-A4ND-01A-11R-A266-07 | 2 | 0.5 | 2.55027696 | DDX20 | High |
| TCGA-2Y-A9GX-01A-11R-A38B-07 | 2 | 71.1 | 2.55572003 | DDX20 | High |
| TCGA-DD-A4NF-01A-11R-A27V-07 | 2 | 21.8666667 | 2.55639834 | DDX20 | High |
| TCGA-ED-A7XP-01A-11R-A352-07 | 2 | 6.23333333 | 2.57077309 | DDX20 | High |
| TCGA-UB-A7ME-01A-11R-A33J-07 | 1 | 16.2 | 2.57194833 | DDX20 | High |
| TCGA-DD-AACX-01A-11R-A41C-07 | 2 | 1.4 | 2.57247545 | DDX20 | High |
| TCGA-DD-AAVV-01A-11R-A41C-07 | 1 | 81.8333333 | 2.58469365 | DDX20 | High |
| TCGA-MI-A75I-01A-11R-A32O-07 | 2 | 8.83333333 | 2.58624378 | DDX20 | High |
| TCGA-FV-A3R2-01A-11R-A22L-07 | 1 | 6.46666667 | 2.5880992 | DDX20 | High |
| TCGA-5C-A9VG-01A-11R-A37K-07 | 1 | 10.9333333 | 2.59029961 | DDX20 | High |
| TCGA-G3-A5SL-01A-11R-A27V-07 | 1 | 20.7 | 2.60504863 | DDX20 | High |
| TCGA-BC-4072-01B-11R-A155-07 | 2 | 15.8333333 | 2.61012056 | DDX20 | High |
| TCGA-G3-AAV1-01A-11R-A38B-07 | 2 | 1.63333333 | 2.61030846 | DDX20 | High |
| TCGA-CC-A7IG-01A-11R-A33J-07 | 2 | 6.5 | 2.6106817 | DDX20 | High |
| TCGA-ED-A66X-01A-11R-A311-07 | 2 | 4.13333333 | 2.61279732 | DDX20 | High |
| TCGA-DD-AAVS-01A-11R-A41C-07 | 1 | 60.7666667 | 2.61489089 | DDX20 | High |
| TCGA-DD-AACA-01A-11R-A41C-07 | 2 | 25.8666667 | 2.63097113 | DDX20 | High |
| TCGA-ED-A7PX-01A-51R-A352-07 | 1 | 0.2 | 2.63267212 | DDX20 | High |
| TCGA-DD-A1EA-01A-11R-A131-07 | 2 | 25.1333333 | 2.64467767 | DDX20 | High |
| TCGA-FV-A3I1-01A-11R-A22L-07 | 1 | 8.23333333 | 2.64472458 | DDX20 | High |
| TCGA-BD-A3EP-01A-11R-A22L-07 | 1 | 13.6333333 | 2.66133284 | DDX20 | High |
| TCGA-UB-A7MC-01A-11R-A33R-07 | 2 | 11.9666667 | 2.67891983 | DDX20 | High |
| TCGA-DD-A1EL-01A-11R-A155-07 | 2 | 13.8333333 | 2.68363746 | DDX20 | High |
| TCGA-EP-A3RK-01A-11R-A22L-07 | 2 | 10.6666667 | 2.68564838 | DDX20 | High |
| TCGA-DD-A1EC-01A-21R-A131-07 | 2 | 9.96666667 | 2.68800547 | DDX20 | High |
| TCGA-CC-A9FV-01A-11R-A37K-07 | 1 | 0 | 2.69757708 | DDX20 | High |
| TCGA-BW-A5NP-01A-11R-A27V-07 | 2 | 3.4 | 2.70028212 | DDX20 | High |
| TCGA-CC-5262-01A-01R-A131-07 | 1 | 3.43333333 | 2.70812766 | DDX20 | High |
| TCGA-DD-AAD8-01A-11R-A41C-07 | 1 | 40.6333333 | 2.71141609 | DDX20 | High |
| TCGA-DD-A4NR-01A-11R-A311-07 | 1 | 0.3 | 2.71176181 | DDX20 | High |
| TCGA-DD-AACA-02A-11R-A41C-07 | 2 | 25.8666667 | 2.71713751 | DDX20 | High |
| TCGA-DD-AAD1-01A-11R-A41C-07 | 2 | 4.23333333 | 2.72085691 | DDX20 | High |
| TCGA-DD-A39Y-01A-11R-A213-07 | 1 | 5.7 | 2.72341779 | DDX20 | High |
| TCGA-DD-AACZ-01A-11R-A41C-07 | 2 | 2.93333333 | 2.72708378 | DDX20 | High |
| TCGA-DD-A11A-01A-11R-A131-07 | 1 | 2.63333333 | 2.73114929 | DDX20 | High |
| TCGA-BC-A3KG-01A-11R-A213-07 | 2 | 6.96666667 | 2.73254636 | DDX20 | High |
| TCGA-ED-A97K-01A-21R-A38B-07 | 1 | 0.2 | 2.73686887 | DDX20 | High |
| TCGA-DD-AADV-01A-11R-A39D-07 | 1 | 19.1333333 | 2.73965352 | DDX20 | High |
| TCGA-XR-A8TG-01A-11R-A36F-07 | 2 | 11.6666667 | 2.74960049 | DDX20 | High |
| TCGA-G3-A5SJ-01A-11R-A27V-07 | 2 | 10.2666667 | 2.7550386 | DDX20 | High |
| TCGA-CC-5258-01A-01R-A131-07 | 1 | 4.3 | 2.75579361 | DDX20 | High |
| TCGA-DD-A39W-01A-11R-A213-07 | 1 | 27.5666667 | 2.75617396 | DDX20 | High |
| TCGA-2Y-A9GY-01A-11R-A38B-07 | 2 | 21.2333333 | 2.75964706 | DDX20 | High |
| TCGA-BC-A10U-01A-11R-A131-07 | 2 | 19.5333333 | 2.76122561 | DDX20 | High |
| TCGA-CC-A8HV-01A-11R-A36F-07 | 2 | 4.96666667 | 2.76586315 | DDX20 | High |
| TCGA-DD-AAW3-01A-11R-A41C-07 | 1 | 54.4333333 | 2.76687975 | DDX20 | High |
| TCGA-GJ-A9DB-01A-11R-A37K-07 | 1 | 2.23333333 | 2.76869336 | DDX20 | High |
| TCGA-FV-A23B-01A-11R-A16W-07 | 2 | 48.4333333 | 2.76917184 | DDX20 | High |
| TCGA-G3-A25U-01A-11R-A16W-07 | 1 | 54.5333333 | 2.7720877 | DDX20 | High |
| TCGA-DD-AAW0-01A-11R-A41C-07 | 1 | 67.1666667 | 2.77846896 | DDX20 | High |
| TCGA-DD-AADP-01A-11R-A39D-07 | 1 | 15.2666667 | 2.78459168 | DDX20 | High |
| TCGA-DD-AADI-01A-11R-A41C-07 | 1 | 36.1666667 | 2.793264 | DDX20 | High |
| TCGA-G3-A3CK-01A-11R-A213-07 | 1 | 19.5 | 2.80130327 | DDX20 | High |
| TCGA-DD-AAC8-01A-11R-A41C-07 | 1 | 0.53333333 | 2.8177149 | DDX20 | High |
| TCGA-DD-AAD0-01A-11R-A41C-07 | 2 | 1.56666667 | 2.83649301 | DDX20 | High |
| TCGA-K7-AAU7-01A-11R-A38B-07 | 2 | 1.93333333 | 2.8373272 | DDX20 | High |
| TCGA-BC-A10Y-01A-11R-A131-07 | 2 | 13.3333333 | 2.84101124 | DDX20 | High |
| TCGA-DD-A4NS-01A-11R-A311-07 | 2 | 29.7666667 | 2.84680985 | DDX20 | High |
| TCGA-DD-A4NJ-01A-11R-A27V-07 | 2 | 18.3 | 2.85441627 | DDX20 | High |
| TCGA-G3-A6UC-01A-21R-A33J-07 | 2 | 21.3 | 2.8562461 | DDX20 | High |
| TCGA-4R-AA8I-01A-11R-A38B-07 | 2 | 5.26666667 | 2.86410933 | DDX20 | High |
| TCGA-DD-AACL-01A-11R-A41C-07 | 1 | 3.56666667 | 2.87034405 | DDX20 | High |
| TCGA-DD-AADY-01A-11R-A41C-07 | 1 | 18.5 | 2.87111977 | DDX20 | High |
| TCGA-G3-AAV4-01A-11R-A38B-07 | 1 | 0.9 | 2.87113002 | DDX20 | High |
| TCGA-BC-A217-01A-11R-A155-07 | 1 | 46.5666667 | 2.87252233 | DDX20 | High |
| TCGA-ZS-A9CF-02A-11R-A38B-07 | 2 | 21.2 | 2.8731392 | DDX20 | High |
| TCGA-CC-A8HU-01A-11R-A36F-07 | 2 | 10.0333333 | 2.87405805 | DDX20 | High |
| TCGA-XR-A8TE-01A-11R-A36F-07 | 2 | 7 | 2.8786682 | DDX20 | High |
| TCGA-XR-A8TF-01A-11R-A36F-07 | 2 | 7.7 | 2.89787789 | DDX20 | High |
| TCGA-DD-A73E-01A-12R-A32O-07 | 1 | 1.46666667 | 2.92392928 | DDX20 | High |
| TCGA-BC-A3KF-01A-11R-A213-07 | 1 | 0.26666667 | 2.92423457 | DDX20 | High |
| TCGA-EP-A12J-01A-11R-A131-07 | 1 | 19 | 2.94675683 | DDX20 | High |
| TCGA-DD-AACB-01A-11R-A41C-07 | 1 | 77.4666667 | 2.94854005 | DDX20 | High |
| TCGA-ZP-A9CV-01A-11R-A38B-07 | 2 | 36.2666667 | 2.95214018 | DDX20 | High |
| TCGA-2Y-A9HA-01A-11R-A39D-07 | 2 | 1.2 | 2.96494192 | DDX20 | High |
| TCGA-UB-A7MA-01A-11R-A33R-07 | 2 | 7.36666667 | 2.96887744 | DDX20 | High |
| TCGA-DD-A1EF-01A-11R-A131-07 | 2 | 13.1333333 | 2.96928521 | DDX20 | High |
| TCGA-DD-A4NA-01A-11R-A266-07 | 1 | 33.6 | 2.99268564 | DDX20 | High |
| TCGA-ZP-A9CZ-01A-11R-A38B-07 | 1 | 23.5333333 | 2.99949464 | DDX20 | High |
| TCGA-CC-A8HT-01A-11R-A36F-07 | 2 | 2.76666667 | 3.00256147 | DDX20 | High |
| TCGA-BC-A216-01A-11R-A155-07 | 1 | 45.0333333 | 3.01102086 | DDX20 | High |
| TCGA-UB-AA0U-01A-11R-A38B-07 | 2 | 3.53333333 | 3.0261909 | DDX20 | High |
| TCGA-RC-A6M3-01A-11R-A32O-07 | 1 | 0 | 3.03016212 | DDX20 | High |
| TCGA-DD-AACK-01A-11R-A41C-07 | 1 | 0.3 | 3.03743157 | DDX20 | High |
| TCGA-LG-A6GG-01A-11R-A311-07 | 1 | 12.9 | 3.04288179 | DDX20 | High |
| TCGA-DD-AACV-01A-11R-A41C-07 | 2 | 11.4666667 | 3.05366798 | DDX20 | High |
| TCGA-QA-A7B7-01A-11R-A32O-07 | 2 | 2.93333333 | 3.06770897 | DDX20 | High |
| TCGA-YA-A8S7-01A-11R-A37K-07 | 2 | 13.2666667 | 3.07279355 | DDX20 | High |
| TCGA-DD-AADB-01A-11R-A41C-07 | 1 | 41.4 | 3.10607076 | DDX20 | High |
| TCGA-CC-A5UD-01A-11R-A28V-07 | 2 | 6.06666667 | 3.10762008 | DDX20 | High |
| TCGA-CC-5264-01A-01R-A131-07 | 1 | 3.4 | 3.11924293 | DDX20 | High |
| TCGA-CC-A3MA-01A-11R-A213-07 | 2 | 8.7 | 3.14742143 | DDX20 | High |
| TCGA-CC-A3M9-01A-11R-A213-07 | 2 | 7.3 | 3.16947509 | DDX20 | High |
| TCGA-2Y-A9GS-01A-12R-A38B-07 | 2 | 3.4 | 3.18526882 | DDX20 | High |
| TCGA-DD-AA3A-01A-11R-A37K-07 | 1 | 13.6666667 | 3.19443027 | DDX20 | High |
| TCGA-DD-A1EE-01A-11R-A131-07 | 2 | 1.46666667 | 3.19885585 | DDX20 | High |
| TCGA-DD-AADC-01A-11R-A41C-07 | 2 | 3.36666667 | 3.20100255 | DDX20 | High |
| TCGA-DD-AACP-01A-11R-A41C-07 | 1 | 13.8333333 | 3.20593796 | DDX20 | High |
| TCGA-BC-4073-01B-02R-A131-07 | 1 | 28.3 | 3.20730323 | DDX20 | High |
| TCGA-DD-A1EJ-01A-11R-A155-07 | 2 | 8.76666667 | 3.2304366 | DDX20 | High |
| TCGA-FV-A4ZQ-01A-11R-A266-07 | 1 | 0.4 | 3.23235495 | DDX20 | High |
| TCGA-CC-A9FU-01A-11R-A37K-07 | 1 | 0 | 3.23278842 | DDX20 | High |
| TCGA-ED-A82E-01A-11R-A352-07 | 1 | 13.6 | 3.26699248 | DDX20 | High |
| TCGA-DD-AACH-01A-11R-A41C-07 | 2 | 2.76666667 | 3.26819925 | DDX20 | High |
| TCGA-CC-5263-01A-01R-A131-07 | 1 | 4.3 | 3.30979823 | DDX20 | High |
| TCGA-5R-AA1C-01A-11R-A41C-07 | 1 | 17.3333333 | 3.31835287 | DDX20 | High |
| TCGA-CC-5261-01A-01R-A131-07 | 1 | 3.23333333 | 3.31928826 | DDX20 | High |
| TCGA-CC-5260-01A-01R-A131-07 | 1 | 2.9 | 3.33327431 | DDX20 | High |
| TCGA-DD-AAVZ-01A-11R-A41C-07 | 1 | 63.3333333 | 3.33351095 | DDX20 | High |
| TCGA-CC-A5UE-01A-11R-A28V-07 | 2 | 5.83333333 | 3.39407369 | DDX20 | High |
| TCGA-G3-AAV7-01A-11R-A38B-07 | 1 | 12.0333333 | 3.3942764 | DDX20 | High |
| TCGA-UB-A7MB-01A-11R-A33R-07 | 2 | 1.6 | 3.39767131 | DDX20 | High |
| TCGA-ED-A5KG-01A-11R-A27V-07 | 2 | 3.66666667 | 3.41275128 | DDX20 | High |
| TCGA-CC-A7IK-01A-12R-A33R-07 | 2 | 5 | 3.45567856 | DDX20 | High |
| TCGA-ED-A8O5-01A-11R-A36F-07 | 1 | 13.5333333 | 3.46618197 | DDX20 | High |
| TCGA-DD-AADD-01A-11R-A41C-07 | 1 | 41.0333333 | 3.47561612 | DDX20 | High |
| TCGA-BC-A8YO-01A-11R-A37K-07 | 2 | 7.33333333 | 3.50942013 | DDX20 | High |
| TCGA-2Y-A9H2-01A-12R-A38B-07 | 1 | 57.7 | 3.52860895 | DDX20 | High |
| TCGA-DD-A114-01A-11R-A131-07 | 1 | 38.3 | 3.5485037 | DDX20 | High |
| TCGA-CC-A7IJ-01A-11R-A33R-07 | 1 | 12.7333333 | 3.56201079 | DDX20 | High |
| TCGA-CC-A8HS-01A-11R-A36F-07 | 2 | 6.7 | 3.56658175 | DDX20 | High |
| TCGA-CC-A1HT-01A-11R-A131-07 | 1 | 3.36666667 | 3.60334041 | DDX20 | High |
| TCGA-CC-A7II-01A-11R-A33J-07 | 2 | 9.26666667 | 3.62753035 | DDX20 | High |
| TCGA-BC-A112-01A-11R-A131-07 | 1 | 5.1 | 3.64017422 | DDX20 | High |
| TCGA-ZP-A9D2-01A-11R-A38B-07 | 2 | 10.5 | 3.65838554 | DDX20 | High |
| TCGA-BW-A5NQ-01A-11R-A27V-07 | 1 | 0 | 3.66898541 | DDX20 | High |
| TCGA-G3-A7M9-01A-23R-A352-07 | 1 | 1.86666667 | 4.00236897 | DDX20 | High |
| TCGA-G3-A7M6-01A-11R-A33R-07 | 2 | 9.76666667 | 4.02958059 | DDX20 | High |
| TCGA-BC-A10W-01A-11R-A131-07 | 2 | 3.03333333 | 4.9422319 | DDX20 | High |

**Supplement. 3 DSS_optimal cutoff value**

| sample_id | event | time | expr | gene | group |
| --- | --- | --- | --- | --- | --- |
| TCGA-DD-AADJ-01A-11R-A41C-07 | 1 | 35.5333333 | 1.06421248 | DDX20 | Low |
| TCGA-DD-A3A2-01A-11R-A213-07 | 1 | 71.0333333 | 1.16526421 | DDX20 | Low |
| TCGA-ES-A2HS-01A-11R-A180-07 | 1 | 22.9333333 | 1.17071244 | DDX20 | Low |
| TCGA-FV-A4ZP-01A-12R-A266-07 | 1 | 82.8666667 | 1.25593027 | DDX20 | Low |
| TCGA-DD-A3A7-01A-11R-A22L-07 | 2 | 13.9666667 | 1.26859986 | DDX20 | Low |
| TCGA-2Y-A9H1-01A-11R-A38B-07 | 2 | 40.9666667 | 1.27912311 | DDX20 | Low |
| TCGA-DD-AAEB-01A-11R-A41C-07 | 1 | 15.9333333 | 1.28265443 | DDX20 | Low |
| TCGA-G3-AAV0-01A-11R-A37K-07 | 1 | 15.8666667 | 1.29509613 | DDX20 | Low |
| TCGA-DD-AADS-01A-11R-A41C-07 | 1 | 15.8 | 1.31969782 | DDX20 | Low |
| TCGA-2Y-A9H3-01A-11R-A38B-07 | 1 | 50.5333333 | 1.33416283 | DDX20 | Low |
| TCGA-CC-5259-01A-31R-A213-07 | 1 | 8.33333333 | 1.35141987 | DDX20 | Low |
| TCGA-DD-A3A9-01A-11R-A266-07 | 2 | 31.0333333 | 1.3974612 | DDX20 | Low |
| TCGA-DD-AADO-01A-11R-A41C-07 | 1 | 15.1 | 1.40066352 | DDX20 | Low |
| TCGA-ES-A2HT-01A-12R-A180-07 | 1 | 14.6 | 1.43357022 | DDX20 | Low |
| TCGA-WQ-AB4B-01A-11R-A41C-07 | 1 | 13.1666667 | 1.45679822 | DDX20 | Low |
| TCGA-RC-A7SH-01A-11R-A38B-07 | 1 | 15.6 | 1.46730412 | DDX20 | Low |
| TCGA-2Y-A9H6-01A-11R-A39D-07 | 1 | 11.9 | 1.49686387 | DDX20 | Low |
| TCGA-DD-A3A3-01A-11R-A22L-07 | | 17.8333333 | 1.5023969 | DDX20 | Low |
| TCGA-2Y-A9GU-01A-11R-A38B-07 | 1 | 64.6333333 | 1.51033494 | DDX20 | Low |
| TCGA-DD-A73G-01A-22R-A32O-07 | 1 | 115.933333 | 1.52872998 | DDX20 | Low |
| TCGA-DD-A39X-01A-11R-A213-07 | 2 | 56.4666667 | 1.5473309 | DDX20 | Low |
| TCGA-NI-A4U2-01A-11R-A28V-07 | 2 | 59.7 | 1.5491896 | DDX20 | Low |
| TCGA-DD-A11C-01A-11R-A131-07 | 1 | 22.0666667 | 1.55170997 | DDX20 | Low |
| TCGA-BC-A10R-01A-11R-A131-07 | 2 | 10.2666667 | 1.55641699 | DDX20 | Low |
| TCGA-DD-AAE1-01A-11R-A41C-07 | 1 | 18.4 | 1.56164519 | DDX20 | Low |
| TCGA-DD-AADU-01A-11R-A41C-07 | 1 | 18.4666667 | 1.58463853 | DDX20 | Low |
| TCGA-NI-A8LF-01A-11R-A36F-07 | 1 | 26.6333333 | 1.60386912 | DDX20 | Low |
| TCGA-DD-AAC9-01A-11R-A41C-07 | 1 | 11.5666667 | 1.61501197 | DDX20 | Low |
| TCGA-DD-A4NG-01A-11R-A27V-07 | 2 | 26.7333333 | 1.6262543 | DDX20 | Low |
| TCGA-DD-A119-01A-11R-A131-07 | | 7.43333333 | 1.63397767 | DDX20 | Low |
| TCGA-DD-A11B-01A-11R-A131-07 | 1 | 0.46666667 | 1.6353811 | DDX20 | Low |
| TCGA-DD-AADN-01A-11R-A41C-07 | 1 | 29.9333333 | 1.63830221 | DDX20 | Low |
| TCGA-DD-AADM-01A-11R-A41C-07 | 1 | 0.4 | 1.64586394 | DDX20 | Low |
| TCGA-DD-A1EK-01A-11R-A213-07 | 2 | 18.6 | 1.65049789 | DDX20 | Low |
| TCGA-EP-A3JL-01A-11R-A213-07 | 1 | 10.1 | 1.65474362 | DDX20 | Low |
| TCGA-G3-A5SM-01A-12R-A28V-07 | 1 | 17.3333333 | 1.6669265 | DDX20 | Low |
| TCGA-2Y-A9GT-01A-11R-A38B-07 | 2 | 54.1333333 | 1.66704031 | DDX20 | Low |
| TCGA-RC-A7SF-01A-11R-A352-07 | 1 | 19.3 | 1.67023264 | DDX20 | Low |
| TCGA-WX-AA47-01A-11R-A39D-07 | 2 | 18.5333333 | 1.67117576 | DDX20 | Low |
| TCGA-DD-A11D-01A-11R-A131-07 | 2 | 52 | 1.67386501 | DDX20 | Low |
| TCGA-G3-A7M5-01A-11R-A33R-07 | 1 | 14.9 | 1.67885141 | DDX20 | Low |
| TCGA-KR-A7K7-01A-11R-A33J-07 | 1 | 31.7 | 1.68311326 | DDX20 | Low |
| TCGA-DD-A4NP-01A-11R-A28V-07 | 1 | 110.266667 | 1.69338093 | DDX20 | Low |
| TCGA-EP-A2KB-01A-11R-A180-07 | 2 | 19.8666667 | 1.69444014 | DDX20 | Low |
| TCGA-5C-A9VH-01A-11R-A37K-07 | 1 | 10.7333333 | 1.71231545 | DDX20 | Low |
| TCGA-DD-AAE3-01A-11R-A41C-07 | 1 | 18.8666667 | 1.72677035 | DDX20 | Low |
| TCGA-DD-A3A6-01A-11R-A22L-07 | 1 | 108.6 | 1.72700077 | DDX20 | Low |
| TCGA-LG-A9QD-01A-11R-A38B-07 | 1 | 12.2 | 1.72751901 | DDX20 | Low |
| TCGA-G3-A5SK-01A-11R-A27V-07 | 1 | 24.8 | 1.73190712 | DDX20 | Low |
| TCGA-DD-A4NL-01A-11R-A28V-07 | 1 | 57.0333333 | 1.73620405 | DDX20 | Low |
| TCGA-BC-A10S-01A-22R-A131-07 | 2 | 47.4333333 | 1.75346611 | DDX20 | Low |
| TCGA-G3-AAUZ-01A-11R-A38B-07 | 1 | 16 | 1.76058938 | DDX20 | Low |
| TCGA-DD-AACD-01A-11R-A41C-07 | 2 | 12.7 | 1.76241874 | DDX20 | Low |
| TCGA-BC-A110-01A-11R-A131-07 | 2 | 70.5333333 | 1.775718 | DDX20 | Low |
| TCGA-RC-A7SB-01A-21R-A352-07 | 1 | 19.6 | 1.78342143 | DDX20 | Low |
| TCGA-DD-A39Z-01A-11R-A213-07 | 1 | 20.0333333 | 1.79499311 | DDX20 | Low |
| TCGA-FV-A3R3-01A-11R-A22L-07 | 1 | 12.2 | 1.80245974 | DDX20 | Low |
| TCGA-G3-AAV6-01A-21R-A37K-07 | 2 | 2.16666667 | 1.80575721 | DDX20 | Low |
| TCGA-DD-AADF-01A-11R-A41C-07 | 1 | 3.83333333 | 1.81358773 | DDX20 | Low |
| TCGA-DD-AADK-01A-11R-A41C-07 | 1 | 34.9666667 | 1.81421643 | DDX20 | Low |
| TCGA-G3-A3CJ-01A-11R-A213-07 | 1 | 19.8 | 1.81619652 | DDX20 | Low |
| TCGA-G3-A25Z-01A-11R-A16W-07 | 1 | 21.8333333 | 1.81932796 | DDX20 | Low |
| TCGA-DD-A4NE-01A-11R-A27V-07 | 2 | 22 | 1.82307083 | DDX20 | Low |
| TCGA-K7-A6G5-01A-11R-A311-07 | 1 | 17.0666667 | 1.82630909 | DDX20 | Low |
| TCGA-G3-A3CI-01A-11R-A213-07 | 1 | 6 | 1.83228142 | DDX20 | Low |
| TCGA-CC-A123-01A-11R-A131-07 | 1 | 7.3 | 1.8325137 | DDX20 | Low |
| TCGA-DD-AADA-01A-11R-A41C-07 | 1 | 41.1 | 1.8352515 | DDX20 | Low |
| TCGA-DD-AAE4-01A-11R-A41C-07 | 1 | 20.2666667 | 1.84067771 | DDX20 | Low |
| TCGA-DD-A73A-01A-12R-A32O-07 | 1 | 24.2666667 | 1.84249609 | DDX20 | Low |
| TCGA-HP-A5MZ-01A-21R-A27V-07 | 1 | 3.03333333 | 1.8425587 | DDX20 | Low |
| TCGA-2Y-A9GW-01A-11R-A38B-07 | 2 | 42.3666667 | 1.84391737 | DDX20 | Low |
| TCGA-DD-A4NI-01A-11R-A27V-07 | 1 | 27.2 | 1.86146197 | DDX20 | Low |
| TCGA-WJ-A86L-01A-12R-A39D-07 | 1 | 11.5 | 1.87332093 | DDX20 | Low |
| TCGA-DD-AAE7-01A-11R-A41C-07 | 1 | 21.4666667 | 1.88061718 | DDX20 | Low |
| TCGA-DD-AAVW-01A-11R-A41C-07 | 1 | 77.2333333 | 1.88507653 | DDX20 | Low |
| TCGA-DD-AACI-01A-11R-A41C-07 | 1 | 53.9333333 | 1.8859475 | DDX20 | Low |
| TCGA-EP-A2KC-01A-11R-A213-07 | 1 | 0.63333333 | 1.88937676 | DDX20 | Low |
| TCGA-DD-A3A8-01A-11R-A22L-07 | 1 | 0.36666667 | 1.89197789 | DDX20 | Low |
| TCGA-DD-AACE-01A-11R-A41C-07 | 1 | 72.8 | 1.89855749 | DDX20 | Low |
| TCGA-KR-A7K2-01A-12R-A33R-07 | 1 | 27.6333333 | 1.89881097 | DDX20 | Low |
| TCGA-DD-A39V-01A-11R-A213-07 | 2 | 21.4333333 | 1.90960075 | DDX20 | Low |
| TCGA-G3-A3CH-01A-11R-A22L-07 | 1 | 26 | 1.91064494 | DDX20 | Low |
| TCGA-CC-A7IF-01A-11R-A33J-07 | 2 | 21.6333333 | 1.91110577 | DDX20 | Low |
| TCGA-MI-A75C-01A-11R-A32O-07 | 1 | 9.7 | 1.91900343 | DDX20 | Low |
| TCGA-BC-A69H-01A-11R-A311-07 | 1 | 14.8 | 1.92523076 | DDX20 | Low |
| TCGA-K7-A5RF-01A-11R-A28V-07 | 1 | 21.0333333 | 1.92642247 | DDX20 | Low |
| TCGA-BD-A3ER-01A-11R-A213-07 | 1 | 37.1666667 | 1.92819697 | DDX20 | Low |
| TCGA-DD-AAEI-01A-11R-A41C-07 | 1 | 51.0333333 | 1.92919097 | DDX20 | Low |
| TCGA-FV-A495-01A-11R-A266-07 | 1 | 0.03333333 | 1.93166149 | DDX20 | Low |
| TCGA-DD-AACW-01A-11R-A41C-07 | 1 | 47.4666667 | 1.93227997 | DDX20 | Low |
| TCGA-DD-AAVR-01A-11R-A41C-07 | 1 | 83.7666667 | 1.93472901 | DDX20 | Low |
| TCGA-DD-AACT-01A-11R-A41C-07 | 1 | 52.0666667 | 1.94267086 | DDX20 | Low |
| TCGA-ZS-A9CD-01A-11R-A37K-07 | | 46.2 | 1.94312951 | DDX20 | Low |
| TCGA-DD-AAEG-01A-11R-A39D-07 | 1 | 23.9666667 | 1.95077057 | DDX20 | Low |
| TCGA-WX-AA46-01A-11R-A39D-07 | 1 | 25.2 | 1.95098088 | DDX20 | Low |
| TCGA-DD-AAE9-01A-11R-A41C-07 | 1 | 24.0666667 | 1.95367449 | DDX20 | Low |
| TCGA-G3-A7M8-01A-11R-A33R-07 | 1 | 14.3333333 | 1.95872743 | DDX20 | Low |
| TCGA-G3-AAV5-01A-11R-A37K-07 | 1 | 11.8 | 1.96055629 | DDX20 | Low |
| TCGA-FV-A3I0-01A-11R-A22L-07 | 1 | 28.2666667 | 1.97020285 | DDX20 | Low |
| TCGA-2Y-A9H4-01A-11R-A38B-07 | 1 | 48.4 | 1.99854291 | DDX20 | Low |
| TCGA-DD-AAVQ-01A-11R-A41C-07 | 1 | 90.9333333 | 2.00592941 | DDX20 | Low |
| TCGA-DD-A1ED-01A-11R-A155-07 | 1 | 76.7 | 2.00717668 | DDX20 | Low |
| TCGA-5R-AAAM-01A-12R-A41C-07 | 1 | 1.53333333 | 2.00737971 | DDX20 | Low |
| TCGA-DD-AAD2-01A-11R-A41C-07 | 1 | 21.9333333 | 2.01474322 | DDX20 | Low |
| TCGA-DD-A115-01A-11R-A131-07 | 2 | 84.7333333 | 2.01624575 | DDX20 | Low |
| TCGA-MI-A75G-01A-11R-A32O-07 | 1 | 23.2666667 | 2.01787599 | DDX20 | Low |
| TCGA-DD-A3A4-01A-11R-A22L-07 | 2 | 20.4 | 2.03754666 | DDX20 | Low |
| TCGA-DD-AACY-01A-11R-A41C-07 | 1 | 48.3333333 | 2.03755383 | DDX20 | Low |
| TCGA-RC-A6M6-01A-11R-A32O-07 | 1 | 0.3 | 2.04509128 | DDX20 | Low |
| TCGA-MR-A8JO-01A-12R-A36F-07 | 1 | 11 | 2.04534158 | DDX20 | Low |
| TCGA-DD-A118-01A-11R-A131-07 | 1 | 114.566667 | 2.04763012 | DDX20 | Low |
| TCGA-BD-A2L6-01A-11R-A213-07 | 1 | 45.4333333 | 2.04980543 | DDX20 | Low |
| TCGA-MR-A520-01A-11R-A266-07 | 1 | 7.63333333 | 2.05514707 | DDX20 | Low |
| TCGA-CC-A3MB-01A-11R-A213-07 | 2 | 10.5 | 2.05762217 | DDX20 | Low |
| TCGA-5C-AAPD-01A-21R-A39D-07 | 1 | 0.66666667 | 2.05811555 | DDX20 | Low |
| TCGA-O8-A75V-01A-11R-A32O-07 | 1 | 17.9333333 | 2.06548958 | DDX20 | Low |
| TCGA-DD-A113-01A-11R-A131-07 | 1 | 80.8333333 | 2.06874972 | DDX20 | Low |
| TCGA-DD-AAEA-01A-11R-A41C-07 | 1 | 19.1666667 | 2.07086742 | DDX20 | Low |
| TCGA-DD-A116-01A-11R-A131-07 | 1 | 54.0666667 | 2.07171149 | DDX20 | Low |
| TCGA-BC-A10X-01A-11R-A131-07 | 1 | 25.6666667 | 2.07414015 | DDX20 | Low |
| TCGA-ED-A7PZ-01A-11R-A33R-07 | 1 | 0.2 | 2.07757736 | DDX20 | Low |
| TCGA-BC-A69I-01A-11R-A311-07 | 1 | 12.9 | 2.07805984 | DDX20 | Low |
| TCGA-ZS-A9CG-01A-11R-A37K-07 | 1 | 11.3666667 | 2.07959572 | DDX20 | Low |
| TCGA-DD-AAW1-01A-11R-A41C-07 | 1 | 66.3 | 2.0867939 | DDX20 | Low |
| TCGA-MI-A75E-01A-11R-A32O-07 | 1 | 16.9 | 2.09083744 | DDX20 | Low |
| TCGA-G3-A7M7-01A-12R-A352-07 | 1 | 12.0333333 | 2.0971436 | DDX20 | Low |
| TCGA-DD-A4NV-01A-11R-A311-07 | 1 | 79.9333333 | 2.09864647 | DDX20 | Low |
| TCGA-DD-AAEK-01A-11R-A41C-07 | 1 | 35.5666667 | 2.10325697 | DDX20 | Low |
| TCGA-CC-A3MC-01A-11R-A22L-07 | 1 | 12.1 | 2.10581808 | DDX20 | Low |
| TCGA-2Y-A9H5-01A-11R-A38B-07 | 2 | 18.5 | 2.12304557 | DDX20 | Low |
| TCGA-DD-AACA-02B-11R-A41C-07 | 1 | 76.7 | 2.12679422 | DDX20 | Low |
| TCGA-XR-A8TC-01A-11R-A36F-07 | 1 | 44.6333333 | 2.13003238 | DDX20 | Low |
| TCGA-DD-AACG-01A-11R-A41C-07 | 2 | 15.6333333 | 2.1312006 | DDX20 | Low |
| TCGA-BC-A10T-01A-11R-A131-07 | 2 | 27.9 | 2.13162481 | DDX20 | Low |
| TCGA-DD-A1EI-01A-11R-A131-07 | 1 | 6.1 | 2.13198571 | DDX20 | Low |
| TCGA-DD-AAD6-01A-11R-A41C-07 | 1 | 22.4 | 2.13510422 | DDX20 | Low |
| TCGA-DD-A3A5-01A-11R-A22L-07 | 2 | 104.166667 | 2.1355512 | DDX20 | Low |
| TCGA-DD-AACO-01A-11R-A41C-07 | 1 | 62.5333333 | 2.13725626 | DDX20 | Low |
| TCGA-DD-AACU-01A-11R-A41C-07 | 1 | 52.2333333 | 2.13767705 | DDX20 | Low |
| TCGA-DD-AAE0-01A-11R-A41C-07 | 1 | 18.5 | 2.13935378 | DDX20 | Low |
| TCGA-G3-A25V-01A-11R-A16W-07 | 1 | 28.6666667 | 2.14745332 | DDX20 | Low |
| TCGA-DD-AAE6-01A-11R-A41C-07 | 1 | 4.7 | 2.15565003 | DDX20 | Low |
| TCGA-XR-A8TD-01A-12R-A39D-07 | 1 | 34.3333333 | 2.15906288 | DDX20 | Low |
| TCGA-DD-AAD3-01A-11R-A41C-07 | 1 | 43.1666667 | 2.16604645 | DDX20 | Low |
| TCGA-EP-A2KA-01A-11R-A180-07 | 2 | 20.9 | 2.16935578 | DDX20 | Low |
| TCGA-DD-A1EH-01A-11R-A131-07 | 1 | 49.8333333 | 2.17955971 | DDX20 | Low |
| TCGA-RC-A7SK-01A-11R-A352-07 | 1 | 15.7333333 | 2.19200955 | DDX20 | Low |
| TCGA-RC-A7S9-01A-11R-A33R-07 | 1 | 21.3333333 | 2.19223796 | DDX20 | Low |
| TCGA-DD-AACQ-01A-11R-A41C-07 | 2 | 14.4 | 2.19242032 | DDX20 | Low |
| TCGA-ZP-A9CY-01A-11R-A38B-07 | 1 | 26.0666667 | 2.20261411 | DDX20 | Low |
| TCGA-BC-A5W4-01A-11R-A28V-07 | 1 | 18.2333333 | 2.20780244 | DDX20 | Low |
| TCGA-DD-A4NB-01A-12R-A266-07 | 1 | 32.9666667 | 2.20842151 | DDX20 | Low |
| TCGA-DD-A3A1-01A-11R-A213-07 | 1 | 7.76666667 | 2.21299134 | DDX20 | Low |
| TCGA-G3-A25X-01A-11R-A16W-07 | 1 | 59.3 | 2.21446914 | DDX20 | Low |
| TCGA-GJ-A6C0-01A-12R-A311-07 | | 1.03333333 | 2.21803674 | DDX20 | Low |
| TCGA-MI-A75H-01A-11R-A32O-07 | 1 | 24.9 | 2.21857284 | DDX20 | Low |
| TCGA-G3-A3CG-01A-11R-A213-07 | 1 | 22.4333333 | 2.22601874 | DDX20 | Low |
| TCGA-ED-A7PY-01A-11R-A33R-07 | 1 | 13 | 2.22685311 | DDX20 | Low |
| TCGA-2Y-A9H9-01A-21R-A39D-07 | 1 | 23.2333333 | 2.23072292 | DDX20 | Low |
| TCGA-WQ-A9G7-01A-11R-A37K-07 | 1 | 1 | 2.23117955 | DDX20 | Low |
| TCGA-DD-AAW2-01A-11R-A41C-07 | 1 | 61.8333333 | 2.23619973 | DDX20 | Low |
| TCGA-DD-AAE2-01A-11R-A41C-07 | 1 | 21.2666667 | 2.2368352 | DDX20 | Low |
| TCGA-ZP-A9D1-01A-11R-A38B-07 | 1 | 0.7 | 2.25444745 | DDX20 | Low |
| TCGA-DD-AACC-01A-11R-A41C-07 | 2 | 56.1666667 | 2.25558876 | DDX20 | Low |
| TCGA-GJ-A3OU-01A-31R-A38B-07 | 1 | 29.3 | 2.25846097 | DDX20 | Low |
| TCGA-UB-A7MF-01A-11R-A33J-07 | 2 | 7.13333333 | 2.25945197 | DDX20 | Low |
| TCGA-KR-A7K8-01A-11R-A33J-07 | 1 | 30.2 | 2.25982063 | DDX20 | Low |
| TCGA-DD-A73F-01A-11R-A32O-07 | 1 | 36.1666667 | 2.26182069 | DDX20 | Low |
| TCGA-DD-A4NK-01A-11R-A28V-07 | 2 | 40.3333333 | 2.26976828 | DDX20 | Low |
| TCGA-G3-A25S-01A-11R-A16W-07 | 2 | 13.8666667 | 2.27031298 | DDX20 | Low |
| TCGA-DD-AADQ-01A-11R-A41C-07 | 1 | 14.5333333 | 2.27348195 | DDX20 | Low |
| TCGA-DD-AAEH-01A-11R-A41C-07 | 1 | 26.1333333 | 2.27445258 | DDX20 | Low |
| TCGA-EP-A26S-01A-11R-A16W-07 | 1 | 20.2666667 | 2.27479464 | DDX20 | Low |
| TCGA-ZP-A9D4-01A-11R-A37K-07 | 1 | 13.1666667 | 2.28266078 | DDX20 | Low |
| TCGA-UB-A7MD-01A-12R-A352-07 | 1 | 1.73333333 | 2.29409094 | DDX20 | Low |
| TCGA-G3-A25T-01A-11R-A16W-07 | 1 | 51.7666667 | 2.29638197 | DDX20 | Low |
| TCGA-T1-A6J8-01A-11R-A32O-07 | 1 | 0.76666667 | 2.29783041 | DDX20 | Low |
| TCGA-ZS-A9CF-01A-11R-A38B-07 | 1 | 80.4 | 2.29894047 | DDX20 | Low |
| TCGA-G3-AAV2-01A-11R-A37K-07 | 1 | 12.4 | 2.30842601 | DDX20 | Low |
| TCGA-DD-A4NH-01A-11R-A27V-07 | 1 | 30.5666667 | 2.30906933 | DDX20 | Low |
| TCGA-FV-A496-01A-11R-A266-07 | 1 | 0.33333333 | 2.31474076 | DDX20 | Low |
| TCGA-UB-AA0V-01A-11R-A38B-07 | 1 | 10.4666667 | 2.31963818 | DDX20 | Low |
| TCGA-DD-AADG-01A-11R-A41C-07 | 1 | 38.1666667 | 2.32085513 | DDX20 | Low |
| TCGA-5R-AA1D-01A-11R-A38B-07 | 1 | 14.9666667 | 2.32095323 | DDX20 | Low |
| TCGA-BC-A10Q-01A-11R-A131-07 | 2 | 37.8333333 | 2.32704767 | DDX20 | Low |
| TCGA-ZS-A9CE-01A-11R-A37K-07 | 1 | 41.3666667 | 2.33780245 | DDX20 | Low |
| TCGA-DD-AAVU-01A-11R-A41C-07 | 1 | 73.4 | 2.34320727 | DDX20 | Low |
| TCGA-G3-AAV3-01A-11R-A37K-07 | 1 | 13.7333333 | 2.34369174 | DDX20 | Low |
| TCGA-RC-A6M5-01A-11R-A32O-07 | 1 | 0.5 | 2.34605602 | DDX20 | Low |
| TCGA-2Y-A9GZ-01A-11R-A39D-07 | 2 | 28.2666667 | 2.35638238 | DDX20 | Low |
| TCGA-CC-A7IE-01A-21R-A38B-07 | 2 | 7.23333333 | 2.35910521 | DDX20 | Low |
| TCGA-KR-A7K0-01A-12R-A33R-07 | 1 | 2.16666667 | 2.36314355 | DDX20 | Low |
| TCGA-DD-AAVX-01A-11R-A41C-07 | 1 | 57.2666667 | 2.36368313 | DDX20 | Low |
| TCGA-ZP-A9D0-01A-11R-A37K-07 | 1 | 36.3666667 | 2.3669836 | DDX20 | Low |
| TCGA-DD-AACN-01A-11R-A41C-07 | 1 | 43.4 | 2.36741985 | DDX20 | Low |
| TCGA-2Y-A9H8-01A-11R-A39D-07 | 2 | 21.1 | 2.37191745 | DDX20 | Low |
| TCGA-CC-A9FW-01A-11R-A37K-07 | 1 | 8.26666667 | 2.38038362 | DDX20 | Low |
| TCGA-DD-A1EG-01A-11R-A213-07 | 2 | 45.7333333 | 2.38077114 | DDX20 | Low |
| TCGA-CC-A9FS-01A-11R-A37K-07 | 1 | 7.03333333 | 2.38198901 | DDX20 | Low |
| TCGA-DD-AACF-01A-11R-A41C-07 | 2 | 12.1666667 | 2.38768645 | DDX20 | Low |
| TCGA-DD-AAED-01A-12R-A41C-07 | 1 | 25.4333333 | 2.39434918 | DDX20 | Low |
| TCGA-HP-A5N0-01A-11R-A28V-07 | 2 | 38.2333333 | 2.39688455 | DDX20 | Low |
| TCGA-RC-A6M4-01A-11R-A32O-07 | 1 | 0.73333333 | 2.39808537 | DDX20 | Low |
| TCGA-ED-A627-01A-12R-A311-07 | 1 | 14.1 | 2.40010195 | DDX20 | Low |
| TCGA-ED-A4XI-01A-11R-A266-07 | 1 | 27.3 | 2.40253189 | DDX20 | Low |
| TCGA-BC-A10Z-01A-11R-A131-07 | 1 | 1.13333333 | 2.41348937 | DDX20 | Low |
| TCGA-DD-A73B-01A-12R-A32O-07 | 2 | 9.43333333 | 2.41876534 | DDX20 | Low |
| TCGA-RG-A7D4-01A-12R-A33R-07 | 1 | 36.6 | 2.42090174 | DDX20 | Low |
| TCGA-CC-A5UC-01A-11R-A28V-07 | 2 | 11.5666667 | 2.42408522 | DDX20 | Low |
| TCGA-3K-AAZ8-01A-12R-A39D-07 | 1 | 13.2 | 2.42503516 | DDX20 | Low |
| TCGA-G3-A5SI-01A-31R-A27V-07 | 2 | 25.6 | 2.42776141 | DDX20 | Low |
| TCGA-DD-A4NQ-01A-21R-A28V-07 | 2 | 12.4333333 | 2.42900478 | DDX20 | Low |
| TCGA-DD-AACJ-01A-11R-A41C-07 | 1 | 70.0666667 | 2.43955117 | DDX20 | Low |
| TCGA-ED-A7XO-01A-11R-A352-07 | 1 | 14.2333333 | 2.44054676 | DDX20 | Low |
| TCGA-2Y-A9GV-01A-11R-A38B-07 | 2 | 84.4 | 2.4446353 | DDX20 | Low |
| TCGA-PD-A5DF-01A-11R-A27V-07 | 2 | 21.3 | 2.44699301 | DDX20 | Low |
| TCGA-DD-AAVP-01A-11R-A41C-07 | 1 | 91.7333333 | 2.45585713 | DDX20 | Low |
| TCGA-DD-A1EB-01A-11R-A131-07 | 1 | 67.2333333 | 2.45707609 | DDX20 | Low |
| TCGA-FV-A2QR-01A-11R-A213-07 | 2 | 19.3666667 | 2.4657471 | DDX20 | Low |
| TCGA-ED-A66Y-01A-11R-A311-07 | 1 | 9.86666667 | 2.46797307 | DDX20 | Low |
| TCGA-FV-A2QQ-01A-11R-A22L-07 | 1 | 24.3 | 2.46960615 | DDX20 | Low |
| TCGA-2Y-A9H0-01A-11R-A38B-07 | 1 | 122.5 | 2.47385179 | DDX20 | Low |
| TCGA-ED-A8O6-01A-11R-A36F-07 | 1 | 1.86666667 | 2.47501249 | DDX20 | Low |
| TCGA-DD-AADR-01A-11R-A41C-07 | 1 | 67.6 | 2.48074529 | DDX20 | Low |
| TCGA-K7-A5RG-01A-11R-A28V-07 | 1 | 17.3 | 2.48327409 | DDX20 | Low |
| TCGA-G3-A25Y-01A-11R-A16W-07 | 2 | 15.0666667 | 2.48470336 | DDX20 | Low |
| TCGA-LG-A9QC-01A-11R-A37K-07 | 1 | 14.1666667 | 2.48807888 | DDX20 | Low |
| TCGA-DD-A4NO-01A-11R-A28V-07 | 1 | 74.8333333 | 2.48969148 | DDX20 | Low |
| TCGA-CC-A7IH-01A-11R-A33J-07 | 1 | 12.1666667 | 2.49013383 | DDX20 | Low |
| TCGA-DD-A4NN-01A-11R-A28V-07 | 2 | 29.9666667 | 2.49027225 | DDX20 | Low |
| TCGA-DD-AADW-01A-11R-A39D-07 | 1 | 19.5666667 | 2.49097416 | DDX20 | Low |
| TCGA-DD-A73C-01A-12R-A33J-07 | 1 | 23.3666667 | 2.4929807 | DDX20 | Low |
| TCGA-BW-A5NO-01A-11R-A27V-07 | 1 | 0.66666667 | 2.49943225 | DDX20 | Low |
| TCGA-WX-AA44-01A-11R-A39D-07 | 1 | 20.5 | 2.50203007 | DDX20 | Low |
| TCGA-2Y-A9H7-01A-11R-A39D-07 | 1 | 38.9333333 | 2.5041661 | DDX20 | Low |
| TCGA-DD-AACS-01A-11R-A41C-07 | 1 | 60.1333333 | 2.50757931 | DDX20 | Low |
| TCGA-CC-A7IL-01A-11R-A33R-07 | 2 | 9.26666667 | 2.51076097 | DDX20 | Low |
| TCGA-ED-A459-01A-11R-A266-07 | 1 | 30.3333333 | 2.51488025 | DDX20 | Low |
| TCGA-DD-AADL-01A-11R-A41C-07 | 1 | 21.2 | 2.51731886 | DDX20 | Low |
| TCGA-2Y-A9HB-01A-11R-A39D-07 | 1 | 8.66666667 | 2.5184042 | DDX20 | Low |
| TCGA-2V-A95S-01A-11R-A37K-07 | 1 |  | 2.52302893 | DDX20 | Low |
| TCGA-DD-A73D-01A-12R-A32O-07 | 1 | 23.1 | 2.52527239 | DDX20 | Low |
| TCGA-DD-AAD5-01A-11R-A41C-07 | 1 | 44.8333333 | 2.52700514 | DDX20 | Low |
| TCGA-DD-AAEE-01A-11R-A41C-07 | 1 | 27 | 2.53872832 | DDX20 | Low |
| TCGA-DD-AAVY-01A-11R-A41C-07 | 1 | 65.6666667 | 2.54041429 | DDX20 | Low |
| TCGA-DD-A4ND-01A-11R-A266-07 | 1 | 91.5333333 | 2.55027696 | DDX20 | Low |
| TCGA-2Y-A9GX-01A-11R-A38B-07 | 1 | 81.4 | 2.55572003 | DDX20 | Low |
| TCGA-DD-A4NF-01A-11R-A27V-07 | 1 | 31.4 | 2.55639834 | DDX20 | Low |
| TCGA-ED-A7XP-01A-11R-A352-07 | 1 | 13.3333333 | 2.57077309 | DDX20 | Low |
| TCGA-UB-A7ME-01A-11R-A33J-07 | 1 | 16.2 | 2.57194833 | DDX20 | Low |
| TCGA-DD-AACX-01A-11R-A41C-07 | 1 | 5.66666667 | 2.57247545 | DDX20 | Low |
| TCGA-DD-AAVV-01A-11R-A41C-07 | 1 | 81.8333333 | 2.58469365 | DDX20 | Low |
| TCGA-MI-A75I-01A-11R-A32O-07 | 1 | 21 | 2.58624378 | DDX20 | Low |
| TCGA-FV-A3R2-01A-11R-A22L-07 | 1 | 6.46666667 | 2.5880992 | DDX20 | Low |
| TCGA-5C-A9VG-01A-11R-A37K-07 | 1 | 10.9333333 | 2.59029961 | DDX20 | Low |
| TCGA-G3-A5SL-01A-11R-A27V-07 | 1 | 20.7 | 2.60504863 | DDX20 | Low |
| TCGA-BC-4072-01B-11R-A155-07 | 2 | 49.6666667 | 2.61012056 | DDX20 | Low |
| TCGA-G3-AAV1-01A-11R-A38B-07 | 2 | 11.9666667 | 2.61030846 | DDX20 | Low |
| TCGA-CC-A7IG-01A-11R-A33J-07 | 2 | 9.96666667 | 2.6106817 | DDX20 | Low |
| TCGA-ED-A66X-01A-11R-A311-07 | 1 | 13.5333333 | 2.61279732 | DDX20 | Low |
| TCGA-DD-AAVS-01A-11R-A41C-07 | 1 | 60.7666667 | 2.61489089 | DDX20 | Low |
| TCGA-DD-AACA-01A-11R-A41C-07 | 1 | 76.7 | 2.63097113 | DDX20 | Low |
| TCGA-ED-A7PX-01A-51R-A352-07 | 1 | 0.2 | 2.63267212 | DDX20 | Low |
| TCGA-DD-A1EA-01A-11R-A131-07 | 1 | 80.5 | 2.64467767 | DDX20 | Low |
| TCGA-FV-A3I1-01A-11R-A22L-07 | 1 | 8.23333333 | 2.64472458 | DDX20 | Low |
| TCGA-BD-A3EP-01A-11R-A22L-07 | 1 | 13.6333333 | 2.66133284 | DDX20 | Low |
| TCGA-UB-A7MC-01A-11R-A33R-07 | 1 | 16.6666667 | 2.67891983 | DDX20 | Low |
| TCGA-DD-A1EL-01A-11R-A155-07 | 2 | 13.8333333 | 2.68363746 | DDX20 | Low |
| TCGA-EP-A3RK-01A-11R-A22L-07 | 1 | 12.1 | 2.68564838 | DDX20 | Low |
| TCGA-DD-A1EC-01A-21R-A131-07 | 1 | 20.0666667 | 2.68800547 | DDX20 | Low |
| TCGA-CC-A9FV-01A-11R-A37K-07 | 1 | 0 | 2.69757708 | DDX20 | Low |
| TCGA-BW-A5NP-01A-11R-A27V-07 | 1 | 0 | 2.70028212 | DDX20 | Low |
| TCGA-CC-5262-01A-01R-A131-07 | 1 | 3.43333333 | 2.70812766 | DDX20 | Low |
| TCGA-DD-AAD8-01A-11R-A41C-07 | 1 | 40.6333333 | 2.71141609 | DDX20 | Low |
| TCGA-DD-A4NR-01A-11R-A311-07 | 1 | 0.3 | 2.71176181 | DDX20 | Low |
| TCGA-DD-AACA-02A-11R-A41C-07 | 1 | 76.7 | 2.71713751 | DDX20 | Low |
| TCGA-DD-AAD1-01A-11R-A41C-07 | 1 | 18.8 | 2.72085691 | DDX20 | Low |
| TCGA-DD-A39Y-01A-11R-A213-07 | 1 | 5.7 | 2.72341779 | DDX20 | High |
| TCGA-DD-AACZ-01A-11R-A41C-07 | 1 | 5.7 | 2.72708378 | DDX20 | High |
| TCGA-DD-A11A-01A-11R-A131-07 | 1 | 2.63333333 | 2.73114929 | DDX20 | High |
| TCGA-BC-A3KG-01A-11R-A213-07 | 1 | 22.6666667 | 2.73254636 | DDX20 | High |
| TCGA-ED-A97K-01A-21R-A38B-07 | 1 | 0.2 | 2.73686887 | DDX20 | High |
| TCGA-DD-AADV-01A-11R-A39D-07 | 1 | 19.1333333 | 2.73965352 | DDX20 | High |
| TCGA-XR-A8TG-01A-11R-A36F-07 | 1 | 29.9333333 | 2.74960049 | DDX20 | High |
| TCGA-G3-A5SJ-01A-11R-A27V-07 | 1 | 23.2666667 | 2.7550386 | DDX20 | High |
| TCGA-CC-5258-01A-01R-A131-07 | 1 | 4.3 | 2.75579361 | DDX20 | High |
| TCGA-DD-A39W-01A-11R-A213-07 | | 27.5666667 | 2.75617396 | DDX20 | High |
| TCGA-2Y-A9GY-01A-11R-A38B-07 | 2 | 25.2333333 | 2.75964706 | DDX20 | High |
| TCGA-BC-A10U-01A-11R-A131-07 | 2 | 27.9 | 2.76122561 | DDX20 | High |
| TCGA-CC-A8HV-01A-11R-A36F-07 | 2 | 9.3 | 2.76586315 | DDX20 | High |
| TCGA-DD-AAW3-01A-11R-A41C-07 | 1 | 54.4333333 | 2.76687975 | DDX20 | High |
| TCGA-GJ-A9DB-01A-11R-A37K-07 | | 2.23333333 | 2.76869336 | DDX20 | High |
| TCGA-FV-A23B-01A-11R-A16W-07 | 2 | 61.7333333 | 2.76917184 | DDX20 | High |
| TCGA-G3-A25U-01A-11R-A16W-07 | 1 | 54.5333333 | 2.7720877 | DDX20 | High |
| TCGA-DD-AAW0-01A-11R-A41C-07 | 1 | 67.1666667 | 2.77846896 | DDX20 | High |
| TCGA-DD-AADP-01A-11R-A39D-07 | 1 | 15.2666667 | 2.78459168 | DDX20 | High |
| TCGA-DD-AADI-01A-11R-A41C-07 | 1 | 36.1666667 | 2.793264 | DDX20 | High |
| TCGA-G3-A3CK-01A-11R-A213-07 | 1 | 19.5 | 2.80130327 | DDX20 | High |
| TCGA-DD-AAC8-01A-11R-A41C-07 | 1 | 0.53333333 | 2.8177149 | DDX20 | High |
| TCGA-DD-AAD0-01A-11R-A41C-07 | 1 | 4.56666667 | 2.83649301 | DDX20 | High |
| TCGA-K7-AAU7-01A-11R-A38B-07 | 1 | 11.9666667 | 2.8373272 | DDX20 | High |
| TCGA-BC-A10Y-01A-11R-A131-07 | 2 | 23.7 | 2.84101124 | DDX20 | High |
| TCGA-DD-A4NS-01A-11R-A311-07 | 2 | 81.8666667 | 2.84680985 | DDX20 | High |
| TCGA-DD-A4NJ-01A-11R-A27V-07 | 1 | 30.9333333 | 2.85441627 | DDX20 | High |
| TCGA-G3-A6UC-01A-21R-A33J-07 | 1 | 22.3666667 | 2.8562461 | DDX20 | High |
| TCGA-4R-AA8I-01A-11R-A38B-07 | 2 | 8.73333333 | 2.86410933 | DDX20 | High |
| TCGA-DD-AACL-01A-11R-A41C-07 | | 3.56666667 | 2.87034405 | DDX20 | High |
| TCGA-DD-AADY-01A-11R-A41C-07 | 1 | 18.5 | 2.87111977 | DDX20 | High |
| TCGA-G3-AAV4-01A-11R-A38B-07 | 1 | 0.9 | 2.87113002 | DDX20 | High |
| TCGA-BC-A217-01A-11R-A155-07 | 1 | 46.5666667 | 2.87252233 | DDX20 | High |
| TCGA-ZS-A9CF-02A-11R-A38B-07 | 1 | 80.4 | 2.8731392 | DDX20 | High |
| TCGA-CC-A8HU-01A-11R-A36F-07 | 2 | 11.4666667 | 2.87405805 | DDX20 | High |
| TCGA-XR-A8TE-01A-11R-A36F-07 | 1 | 30.8333333 | 2.8786682 | DDX20 | High |
| TCGA-XR-A8TF-01A-11R-A36F-07 | 1 | 23.1 | 2.89787789 | DDX20 | High |
| TCGA-DD-A73E-01A-12R-A32O-07 | 1 | 1.46666667 | 2.92392928 | DDX20 | High |
| TCGA-BC-A3KF-01A-11R-A213-07 | 1 | 0.26666667 | 2.92423457 | DDX20 | High |
| TCGA-EP-A12J-01A-11R-A131-07 | 1 | 19 | 2.94675683 | DDX20 | High |
| TCGA-DD-AACB-01A-11R-A41C-07 | 1 | 77.4666667 | 2.94854005 | DDX20 | High |
| TCGA-ZP-A9CV-01A-11R-A38B-07 | 2 | 36.2666667 | 2.95214018 | DDX20 | High |
| TCGA-2Y-A9HA-01A-11R-A39D-07 | 2 | 1.2 | 2.96494192 | DDX20 | High |
| TCGA-UB-A7MA-01A-11R-A33R-07 | 1 | 28.2666667 | 2.96887744 | DDX20 | High |
| TCGA-DD-A1EF-01A-11R-A131-07 | 2 | 13.1333333 | 2.96928521 | DDX20 | High |
| TCGA-DD-A4NA-01A-11R-A266-07 | 1 | 33.6 | 2.99268564 | DDX20 | High |
| TCGA-ZP-A9CZ-01A-11R-A38B-07 | 1 | 23.5333333 | 2.99949464 | DDX20 | High |
| TCGA-CC-A8HT-01A-11R-A36F-07 | 2 | 4.66666667 | 3.00256147 | DDX20 | High |
| TCGA-BC-A216-01A-11R-A155-07 | 1 | 45.0333333 | 3.01102086 | DDX20 | High |
| TCGA-UB-AA0U-01A-11R-A38B-07 | 1 | 10.9 | 3.0261909 | DDX20 | High |
| TCGA-RC-A6M3-01A-11R-A32O-07 | 1 | 0 | 3.03016212 | DDX20 | High |
| TCGA-DD-AACK-01A-11R-A41C-07 | 1 | 0.3 | 3.03743157 | DDX20 | High |
| TCGA-LG-A6GG-01A-11R-A311-07 | 1 | 12.9 | 3.04288179 | DDX20 | High |
| TCGA-DD-AACV-01A-11R-A41C-07 | 1 | 51.0333333 | 3.05366798 | DDX20 | High |
| TCGA-QA-A7B7-01A-11R-A32O-07 | 1 | 3.13333333 | 3.06770897 | DDX20 | High |
| TCGA-YA-A8S7-01A-11R-A37K-07 | 2 | 13.7333333 | 3.07279355 | DDX20 | High |
| TCGA-DD-AADB-01A-11R-A41C-07 | 1 | 41.4 | 3.10607076 | DDX20 | High |
| TCGA-CC-A5UD-01A-11R-A28V-07 | 2 | 10.1333333 | 3.10762008 | DDX20 | High |
| TCGA-CC-5264-01A-01R-A131-07 | 1 | 3.4 | 3.11924293 | DDX20 | High |
| TCGA-CC-A3MA-01A-11R-A213-07 | 2 | 10.1 | 3.14742143 | DDX20 | High |
| TCGA-CC-A3M9-01A-11R-A213-07 | 2 | 10 | 3.16947509 | DDX20 | High |
| TCGA-2Y-A9GS-01A-12R-A38B-07 | 2 | 24.1333333 | 3.18526882 | DDX20 | High |
| TCGA-DD-AA3A-01A-11R-A37K-07 | 1 | 13.6666667 | 3.19443027 | DDX20 | High |
| TCGA-DD-A1EE-01A-11R-A131-07 | 2 | 11.6333333 | 3.19885585 | DDX20 | High |
| TCGA-DD-AADC-01A-11R-A41C-07 | 2 | 14.1666667 | 3.20100255 | DDX20 | High |
| TCGA-DD-AACP-01A-11R-A41C-07 | 1 | 13.8333333 | 3.20593796 | DDX20 | High |
| TCGA-BC-4073-01B-02R-A131-07 | 1 | 28.3 | 3.20730323 | DDX20 | High |
| TCGA-DD-A1EJ-01A-11R-A155-07 | 2 | 33.5 | 3.2304366 | DDX20 | High |
| TCGA-FV-A4ZQ-01A-11R-A266-07 | 1 | 0.4 | 3.23235495 | DDX20 | High |
| TCGA-CC-A9FU-01A-11R-A37K-07 | 1 | 0 | 3.23278842 | DDX20 | High |
| TCGA-ED-A82E-01A-11R-A352-07 | 1 | 13.6 | 3.26699248 | DDX20 | High |
| TCGA-DD-AACH-01A-11R-A41C-07 | 1 | 6.5 | 3.26819925 | DDX20 | High |
| TCGA-CC-5263-01A-01R-A131-07 | 1 | 4.3 | 3.30979823 | DDX20 | High |
| TCGA-5R-AA1C-01A-11R-A41C-07 | 1 | 17.3333333 | 3.31835287 | DDX20 | High |
| TCGA-CC-5261-01A-01R-A131-07 | 1 | 3.23333333 | 3.31928826 | DDX20 | High |
| TCGA-CC-5260-01A-01R-A131-07 | 1 | 2.9 | 3.33327431 | DDX20 | High |
| TCGA-DD-AAVZ-01A-11R-A41C-07 | 1 | 63.3333333 | 3.33351095 | DDX20 | High |
| TCGA-CC-A5UE-01A-11R-A28V-07 | 2 | 9.06666667 | 3.39407369 | DDX20 | High |
| TCGA-G3-AAV7-01A-11R-A38B-07 | 1 | 12.0333333 | 3.3942764 | DDX20 | High |
| TCGA-UB-A7MB-01A-11R-A33R-07 | 1 | 20.0333333 | 3.39767131 | DDX20 | High |
| TCGA-ED-A5KG-01A-11R-A27V-07 | 1 | 28.4666667 | 3.41275128 | DDX20 | High |
| TCGA-CC-A7IK-01A-12R-A33R-07 | 2 | 8.73333333 | 3.45567856 | DDX20 | High |
| TCGA-ED-A8O5-01A-11R-A36F-07 | 1 | 13.5333333 | 3.46618197 | DDX20 | High |
| TCGA-DD-AADD-01A-11R-A41C-07 | 1 | 41.0333333 | 3.47561612 | DDX20 | High |
| TCGA-BC-A8YO-01A-11R-A37K-07 | 1 | 18.7333333 | 3.50942013 | DDX20 | High |
| TCGA-2Y-A9H2-01A-12R-A38B-07 | 1 | 57.7 | 3.52860895 | DDX20 | High |
| TCGA-DD-A114-01A-11R-A131-07 | 1 | 38.3 | 3.5485037 | DDX20 | High |
| TCGA-CC-A7IJ-01A-11R-A33R-07 | 1 | 12.7333333 | 3.56201079 | DDX20 | High |
| TCGA-CC-A8HS-01A-11R-A36F-07 | 2 | 10 | 3.56658175 | DDX20 | High |
| TCGA-CC-A1HT-01A-11R-A131-07 | 1 | 3.36666667 | 3.60334041 | DDX20 | High |
| TCGA-CC-A7II-01A-11R-A33J-07 | 1 | 13.3 | 3.62753035 | DDX20 | High |
| TCGA-BC-A112-01A-11R-A131-07 | 1 | 5.1 | 3.64017422 | DDX20 | High |
| TCGA-ZP-A9D2-01A-11R-A38B-07 | 2 | 25.5 | 3.65838554 | DDX20 | High |
| TCGA-BW-A5NQ-01A-11R-A27V-07 | 1 | 0 | 3.66898541 | DDX20 | High |
| TCGA-G3-A7M9-01A-23R-A352-07 | | 1.86666667 | 4.00236897 | DDX20 | High |
| TCGA-G3-A7M6-01A-11R-A33R-07 | 1 | 21.0666667 | 4.02958059 | DDX20 | High |
| TCGA-BC-A10W-01A-11R-A131-07 | 2 | 3.03333333 | 4.9422319 | DDX20 | High |

**Supplement.4 OS median value**

| sample_id | event | time | expr | gene | group |
| --- | --- | --- | --- | --- | --- |
| TCGA-DD-AADJ-01A-11R-A41C-07 | 1 | 35.53333 | 1.064212 | DDX20 | Low |
| TCGA-DD-A3A2-01A-11R-A213-07 | 2 | 71.03333 | 1.165264 | DDX20 | Low |
| TCGA-ES-A2HS-01A-11R-A180-07 | 2 | 22.93333 | 1.170712 | DDX20 | Low |
| TCGA-FV-A4ZP-01A-12R-A266-07 | 2 | 82.86667 | 1.25593 | DDX20 | Low |
| TCGA-DD-A3A7-01A-11R-A22L-07 | 2 | 13.96667 | 1.2686 | DDX20 | Low |
| TCGA-2Y-A9H1-01A-11R-A38B-07 | 2 | 40.96667 | 1.279123 | DDX20 | Low |
| TCGA-DD-AAEB-01A-11R-A41C-07 | 1 | 15.93333 | 1.282654 | DDX20 | Low |
| TCGA-G3-AAV0-01A-11R-A37K-07 | 1 | 15.86667 | 1.295096 | DDX20 | Low |
| TCGA-DD-AADS-01A-11R-A41C-07 | 1 | 15.8 | 1.319698 | DDX20 | Low |
| TCGA-2Y-A9H3-01A-11R-A38B-07 | 1 | 50.53333 | 1.334163 | DDX20 | Low |
| TCGA-CC-5259-01A-31R-A213-07 | 1 | 8.333333 | 1.35142 | DDX20 | Low |
| TCGA-DD-A3A9-01A-11R-A266-07 | 2 | 31.03333 | 1.397461 | DDX20 | Low |
| TCGA-DD-AADO-01A-11R-A41C-07 | 1 | 15.1 | 1.400664 | DDX20 | Low |
| TCGA-ES-A2HT-01A-12R-A180-07 | 2 | 14.6 | 1.43357 | DDX20 | Low |
| TCGA-WQ-AB4B-01A-11R-A41C-07 | 1 | 13.16667 | 1.456798 | DDX20 | Low |
| TCGA-RC-A7SH-01A-11R-A38B-07 | 1 | 15.6 | 1.467304 | DDX20 | Low |
| TCGA-2Y-A9H6-01A-11R-A39D-07 | 1 | 11.9 | 1.496864 | DDX20 | Low |
| TCGA-DD-A3A3-01A-11R-A22L-07 | 2 | 17.83333 | 1.502397 | DDX20 | Low |
| TCGA-2Y-A9GU-01A-11R-A38B-07 | 1 | 64.63333 | 1.510335 | DDX20 | Low |
| TCGA-DD-A73G-01A-22R-A32O-07 | 1 | 115.9333 | 1.52873 | DDX20 | Low |
| TCGA-DD-A39X-01A-11R-A213-07 | 2 | 56.46667 | 1.547331 | DDX20 | Low |
| TCGA-NI-A4U2-01A-11R-A28V-07 | 2 | 59.7 | 1.54919 | DDX20 | Low |
| TCGA-DD-A11C-01A-11R-A131-07 | 1 | 22.06667 | 1.55171 | DDX20 | Low |
| TCGA-BC-A10R-01A-11R-A131-07 | 2 | 10.26667 | 1.556417 | DDX20 | Low |
| TCGA-DD-AAE1-01A-11R-A41C-07 | 1 | 18.4 | 1.561645 | DDX20 | Low |
| TCGA-DD-AADU-01A-11R-A41C-07 | 1 | 18.46667 | 1.584639 | DDX20 | Low |
| TCGA-NI-A8LF-01A-11R-A36F-07 | 1 | 26.63333 | 1.603869 | DDX20 | Low |
| TCGA-DD-AAC9-01A-11R-A41C-07 | 1 | 11.56667 | 1.615012 | DDX20 | Low |
| TCGA-DD-A4NG-01A-11R-A27V-07 | 2 | 26.73333 | 1.626254 | DDX20 | Low |
| TCGA-DD-A119-01A-11R-A131-07 | 2 | 7.433333 | 1.633978 | DDX20 | Low |
| TCGA-DD-A11B-01A-11R-A131-07 | 2 | 0.466667 | 1.635381 | DDX20 | Low |
| TCGA-DD-AADN-01A-11R-A41C-07 | 1 | 29.93333 | 1.638302 | DDX20 | Low |
| TCGA-DD-AADM-01A-11R-A41C-07 | 2 | 0.4 | 1.645864 | DDX20 | Low |
| TCGA-DD-A1EK-01A-11R-A213-07 | 2 | 18.6 | 1.650498 | DDX20 | Low |
| TCGA-EP-A3JL-01A-11R-A213-07 | 1 | 10.1 | 1.654744 | DDX20 | Low |
| TCGA-G3-A5SM-01A-12R-A28V-07 | 1 | 17.33333 | 1.666926 | DDX20 | Low |
| TCGA-2Y-A9GT-01A-11R-A38B-07 | 2 | 54.13333 | 1.66704 | DDX20 | Low |
| TCGA-RC-A7SF-01A-11R-A352-07 | 1 | 19.3 | 1.670233 | DDX20 | Low |
| TCGA-WX-AA47-01A-11R-A39D-07 | 2 | 18.53333 | 1.671176 | DDX20 | Low |
| TCGA-DD-A11D-01A-11R-A131-07 | 2 | 52 | 1.673865 | DDX20 | Low |
| TCGA-G3-A7M5-01A-11R-A33R-07 | 1 | 14.9 | 1.678851 | DDX20 | Low |
| TCGA-KR-A7K7-01A-11R-A33J-07 | 1 | 31.7 | 1.683113 | DDX20 | Low |
| TCGA-DD-A4NP-01A-11R-A28V-07 | 1 | 110.2667 | 1.693381 | DDX20 | Low |
| TCGA-EP-A2KB-01A-11R-A180-07 | 2 | 19.86667 | 1.69444 | DDX20 | Low |
| TCGA-5C-A9VH-01A-11R-A37K-07 | 1 | 10.73333 | 1.712315 | DDX20 | Low |
| TCGA-DD-AAE3-01A-11R-A41C-07 | 1 | 18.86667 | 1.72677 | DDX20 | Low |
| TCGA-DD-A3A6-01A-11R-A22L-07 | 2 | 108.6 | 1.727001 | DDX20 | Low |
| TCGA-LG-A9QD-01A-11R-A38B-07 | 1 | 12.2 | 1.727519 | DDX20 | Low |
| TCGA-G3-A5SK-01A-11R-A27V-07 | 1 | 24.8 | 1.731907 | DDX20 | Low |
| TCGA-DD-A4NL-01A-11R-A28V-07 | 1 | 57.03333 | 1.736204 | DDX20 | Low |
| TCGA-BC-A10S-01A-22R-A131-07 | 2 | 47.43333 | 1.753466 | DDX20 | Low |
| TCGA-G3-AAUZ-01A-11R-A38B-07 | 1 | 16 | 1.760589 | DDX20 | Low |
| TCGA-DD-AACD-01A-11R-A41C-07 | 2 | 12.7 | 1.762419 | DDX20 | Low |
| TCGA-BC-A110-01A-11R-A131-07 | 2 | 70.53333 | 1.775718 | DDX20 | Low |
| TCGA-RC-A7SB-01A-21R-A352-07 | 1 | 19.6 | 1.783421 | DDX20 | Low |
| TCGA-DD-A39Z-01A-11R-A213-07 | 2 | 20.03333 | 1.794993 | DDX20 | Low |
| TCGA-FV-A3R3-01A-11R-A22L-07 | 2 | 12.2 | 1.80246 | DDX20 | Low |
| TCGA-G3-AAV6-01A-21R-A37K-07 | 2 | 2.166667 | 1.805757 | DDX20 | Low |
| TCGA-DD-AADF-01A-11R-A41C-07 | 2 | 3.833333 | 1.813588 | DDX20 | Low |
| TCGA-DD-AADK-01A-11R-A41C-07 | 1 | 34.96667 | 1.814216 | DDX20 | Low |
| TCGA-G3-A3CJ-01A-11R-A213-07 | 1 | 19.8 | 1.816197 | DDX20 | Low |
| TCGA-G3-A25Z-01A-11R-A16W-07 | 1 | 21.83333 | 1.819328 | DDX20 | Low |
| TCGA-DD-A4NE-01A-11R-A27V-07 | 2 | 22 | 1.823071 | DDX20 | Low |
| TCGA-K7-A6G5-01A-11R-A311-07 | 1 | 17.06667 | 1.826309 | DDX20 | Low |
| TCGA-G3-A3CI-01A-11R-A213-07 | 1 | 6 | 1.832281 | DDX20 | Low |
| TCGA-CC-A123-01A-11R-A131-07 | 1 | 7.3 | 1.832514 | DDX20 | Low |
| TCGA-DD-AADA-01A-11R-A41C-07 | 1 | 41.1 | 1.835252 | DDX20 | Low |
| TCGA-DD-AAE4-01A-11R-A41C-07 | 1 | 20.26667 | 1.840678 | DDX20 | Low |
| TCGA-DD-A73A-01A-12R-A32O-07 | 1 | 24.26667 | 1.842496 | DDX20 | Low |
| TCGA-HP-A5MZ-01A-21R-A27V-07 | 2 | 3.033333 | 1.842559 | DDX20 | Low |
| TCGA-2Y-A9GW-01A-11R-A38B-07 | 2 | 42.36667 | 1.843917 | DDX20 | Low |
| TCGA-DD-A4NI-01A-11R-A27V-07 | 1 | 27.2 | 1.861462 | DDX20 | Low |
| TCGA-WJ-A86L-01A-12R-A39D-07 | 1 | 11.5 | 1.873321 | DDX20 | Low |
| TCGA-DD-AAE7-01A-11R-A41C-07 | 1 | 21.46667 | 1.880617 | DDX20 | Low |
| TCGA-DD-AAVW-01A-11R-A41C-07 | 1 | 77.23333 | 1.885077 | DDX20 | Low |
| TCGA-DD-AACI-01A-11R-A41C-07 | 1 | 53.93333 | 1.885948 | DDX20 | Low |
| TCGA-EP-A2KC-01A-11R-A213-07 | 2 | 0.633333 | 1.889377 | DDX20 | Low |
| TCGA-DD-A3A8-01A-11R-A22L-07 | 2 | 0.366667 | 1.891978 | DDX20 | Low |
| TCGA-DD-AACE-01A-11R-A41C-07 | 1 | 72.8 | 1.898557 | DDX20 | Low |
| TCGA-KR-A7K2-01A-12R-A33R-07 | 1 | 27.63333 | 1.898811 | DDX20 | Low |
| TCGA-DD-A39V-01A-11R-A213-07 | 2 | 21.43333 | 1.909601 | DDX20 | Low |
| TCGA-G3-A3CH-01A-11R-A22L-07 | 1 | 26 | 1.910645 | DDX20 | Low |
| TCGA-CC-A7IF-01A-11R-A33J-07 | 2 | 21.63333 | 1.911106 | DDX20 | Low |
| TCGA-MI-A75C-01A-11R-A32O-07 | 1 | 9.7 | 1.919003 | DDX20 | Low |
| TCGA-BC-A69H-01A-11R-A311-07 | 1 | 14.8 | 1.925231 | DDX20 | Low |
| TCGA-K7-A5RF-01A-11R-A28V-07 | 1 | 21.03333 | 1.926422 | DDX20 | Low |
| TCGA-BD-A3ER-01A-11R-A213-07 | 1 | 37.16667 | 1.928197 | DDX20 | Low |
| TCGA-DD-AAEI-01A-11R-A41C-07 | 1 | 51.03333 | 1.929191 | DDX20 | Low |
| TCGA-FV-A495-01A-11R-A266-07 | 1 | 0.033333 | 1.931661 | DDX20 | Low |
| TCGA-DD-AACW-01A-11R-A41C-07 | 1 | 47.46667 | 1.93228 | DDX20 | Low |
| TCGA-DD-AAVR-01A-11R-A41C-07 | 1 | 83.76667 | 1.934729 | DDX20 | Low |
| TCGA-DD-AACT-01A-11R-A41C-07 | 1 | 52.06667 | 1.942671 | DDX20 | Low |
| TCGA-ZS-A9CD-01A-11R-A37K-07 | 2 | 46.2 | 1.94313 | DDX20 | Low |
| TCGA-DD-AAEG-01A-11R-A39D-07 | 1 | 23.96667 | 1.950771 | DDX20 | Low |
| TCGA-WX-AA46-01A-11R-A39D-07 | 1 | 25.2 | 1.950981 | DDX20 | Low |
| TCGA-DD-AAE9-01A-11R-A41C-07 | 1 | 24.06667 | 1.953674 | DDX20 | Low |
| TCGA-G3-A7M8-01A-11R-A33R-07 | 1 | 14.33333 | 1.958727 | DDX20 | Low |
| TCGA-G3-AAV5-01A-11R-A37K-07 | 1 | 11.8 | 1.960556 | DDX20 | Low |
| TCGA-FV-A3I0-01A-11R-A22L-07 | 1 | 28.26667 | 1.970203 | DDX20 | Low |
| TCGA-2Y-A9H4-01A-11R-A38B-07 | 1 | 48.4 | 1.998543 | DDX20 | Low |
| TCGA-DD-AAVQ-01A-11R-A41C-07 | 1 | 90.93333 | 2.005929 | DDX20 | Low |
| TCGA-DD-A1ED-01A-11R-A155-07 | 1 | 76.7 | 2.007177 | DDX20 | Low |
| TCGA-5R-AAAM-01A-12R-A41C-07 | 2 | 1.533333 | 2.00738 | DDX20 | Low |
| TCGA-DD-AAD2-01A-11R-A41C-07 | 1 | 21.93333 | 2.014743 | DDX20 | Low |
| TCGA-DD-A115-01A-11R-A131-07 | 2 | 84.73333 | 2.016246 | DDX20 | Low |
| TCGA-MI-A75G-01A-11R-A32O-07 | 1 | 23.26667 | 2.017876 | DDX20 | Low |
| TCGA-DD-A3A4-01A-11R-A22L-07 | 2 | 20.4 | 2.037547 | DDX20 | Low |
| TCGA-DD-AACY-01A-11R-A41C-07 | 1 | 48.33333 | 2.037554 | DDX20 | Low |
| TCGA-RC-A6M6-01A-11R-A32O-07 | 1 | 0.3 | 2.045091 | DDX20 | Low |
| TCGA-MR-A8JO-01A-12R-A36F-07 | 1 | 11 | 2.045342 | DDX20 | Low |
| TCGA-DD-A118-01A-11R-A131-07 | 1 | 114.5667 | 2.04763 | DDX20 | Low |
| TCGA-BD-A2L6-01A-11R-A213-07 | 1 | 45.43333 | 2.049805 | DDX20 | Low |
| TCGA-MR-A520-01A-11R-A266-07 | 1 | 7.633333 | 2.055147 | DDX20 | Low |
| TCGA-CC-A3MB-01A-11R-A213-07 | 2 | 10.5 | 2.057622 | DDX20 | Low |
| TCGA-5C-AAPD-01A-21R-A39D-07 | 1 | 0.666667 | 2.058116 | DDX20 | Low |
| TCGA-O8-A75V-01A-11R-A32O-07 | 1 | 17.93333 | 2.06549 | DDX20 | Low |
| TCGA-DD-A113-01A-11R-A131-07 | 1 | 80.83333 | 2.06875 | DDX20 | Low |
| TCGA-DD-AAEA-01A-11R-A41C-07 | 1 | 19.16667 | 2.070867 | DDX20 | Low |
| TCGA-DD-A116-01A-11R-A131-07 | 2 | 54.06667 | 2.071711 | DDX20 | Low |
| TCGA-BC-A10X-01A-11R-A131-07 | 2 | 25.66667 | 2.07414 | DDX20 | Low |
| TCGA-ED-A7PZ-01A-11R-A33R-07 | 1 | 0.2 | 2.077577 | DDX20 | Low |
| TCGA-BC-A69I-01A-11R-A311-07 | 1 | 12.9 | 2.07806 | DDX20 | Low |
| TCGA-ZS-A9CG-01A-11R-A37K-07 | 1 | 11.36667 | 2.079596 | DDX20 | Low |
| TCGA-DD-AAW1-01A-11R-A41C-07 | 1 | 66.3 | 2.086794 | DDX20 | Low |
| TCGA-MI-A75E-01A-11R-A32O-07 | 1 | 16.9 | 2.090837 | DDX20 | Low |
| TCGA-G3-A7M7-01A-12R-A352-07 | 1 | 12.03333 | 2.097144 | DDX20 | Low |
| TCGA-DD-A4NV-01A-11R-A311-07 | 1 | 79.93333 | 2.098646 | DDX20 | Low |
| TCGA-DD-AAEK-01A-11R-A41C-07 | 1 | 35.56667 | 2.103257 | DDX20 | Low |
| TCGA-CC-A3MC-01A-11R-A22L-07 | 1 | 12.1 | 2.105818 | DDX20 | Low |
| TCGA-2Y-A9H5-01A-11R-A38B-07 | 2 | 18.5 | 2.123046 | DDX20 | Low |
| TCGA-DD-AACA-02B-11R-A41C-07 | 1 | 76.7 | 2.126794 | DDX20 | Low |
| TCGA-XR-A8TC-01A-11R-A36F-07 | 1 | 44.63333 | 2.130032 | DDX20 | Low |
| TCGA-DD-AACG-01A-11R-A41C-07 | 2 | 15.63333 | 2.131201 | DDX20 | Low |
| TCGA-BC-A10T-01A-11R-A131-07 | 2 | 27.9 | 2.131625 | DDX20 | Low |
| TCGA-DD-A1EI-01A-11R-A131-07 | 1 | 6.1 | 2.131986 | DDX20 | Low |
| TCGA-DD-AAD6-01A-11R-A41C-07 | 1 | 22.4 | 2.135104 | DDX20 | Low |
| TCGA-DD-A3A5-01A-11R-A22L-07 | 2 | 104.1667 | 2.135551 | DDX20 | Low |
| TCGA-DD-AACO-01A-11R-A41C-07 | 1 | 62.53333 | 2.137256 | DDX20 | Low |
| TCGA-DD-AACU-01A-11R-A41C-07 | 1 | 52.23333 | 2.137677 | DDX20 | Low |
| TCGA-DD-AAE0-01A-11R-A41C-07 | 1 | 18.5 | 2.139354 | DDX20 | Low |
| TCGA-G3-A25V-01A-11R-A16W-07 | 1 | 28.66667 | 2.147453 | DDX20 | Low |
| TCGA-DD-AAE6-01A-11R-A41C-07 | 1 | 4.7 | 2.15565 | DDX20 | Low |
| TCGA-XR-A8TD-01A-12R-A39D-07 | 1 | 34.33333 | 2.159063 | DDX20 | Low |
| TCGA-DD-AAD3-01A-11R-A41C-07 | 1 | 43.16667 | 2.166046 | DDX20 | Low |
| TCGA-EP-A2KA-01A-11R-A180-07 | 2 | 20.9 | 2.169356 | DDX20 | Low |
| TCGA-DD-A1EH-01A-11R-A131-07 | 1 | 49.83333 | 2.17956 | DDX20 | Low |
| TCGA-RC-A7SK-01A-11R-A352-07 | 1 | 15.73333 | 2.19201 | DDX20 | Low |
| TCGA-RC-A7S9-01A-11R-A33R-07 | 1 | 21.33333 | 2.192238 | DDX20 | Low |
| TCGA-DD-AACQ-01A-11R-A41C-07 | 2 | 14.4 | 2.19242 | DDX20 | Low |
| TCGA-ZP-A9CY-01A-11R-A38B-07 | 1 | 26.06667 | 2.202614 | DDX20 | Low |
| TCGA-BC-A5W4-01A-11R-A28V-07 | 2 | 18.23333 | 2.207802 | DDX20 | Low |
| TCGA-DD-A4NB-01A-12R-A266-07 | 1 | 32.96667 | 2.208422 | DDX20 | Low |
| TCGA-DD-A3A1-01A-11R-A213-07 | 2 | 7.766667 | 2.212991 | DDX20 | Low |
| TCGA-G3-A25X-01A-11R-A16W-07 | 1 | 59.3 | 2.214469 | DDX20 | Low |
| TCGA-GJ-A6C0-01A-12R-A311-07 | 2 | 1.033333 | 2.218037 | DDX20 | Low |
| TCGA-MI-A75H-01A-11R-A32O-07 | 1 | 24.9 | 2.218573 | DDX20 | Low |
| TCGA-G3-A3CG-01A-11R-A213-07 | 1 | 22.43333 | 2.226019 | DDX20 | Low |
| TCGA-ED-A7PY-01A-11R-A33R-07 | 1 | 13 | 2.226853 | DDX20 | Low |
| TCGA-2Y-A9H9-01A-21R-A39D-07 | 1 | 23.23333 | 2.230723 | DDX20 | Low |
| TCGA-WQ-A9G7-01A-11R-A37K-07 | 1 | 1 | 2.23118 | DDX20 | Low |
| TCGA-DD-AAW2-01A-11R-A41C-07 | 1 | 61.83333 | 2.2362 | DDX20 | Low |
| TCGA-DD-AAE2-01A-11R-A41C-07 | 1 | 21.26667 | 2.236835 | DDX20 | Low |
| TCGA-ZP-A9D1-01A-11R-A38B-07 | 1 | 0.7 | 2.254447 | DDX20 | Low |
| TCGA-DD-AACC-01A-11R-A41C-07 | 2 | 56.16667 | 2.255589 | DDX20 | Low |
| TCGA-GJ-A3OU-01A-31R-A38B-07 | 1 | 29.3 | 2.258461 | DDX20 | Low |
| TCGA-UB-A7MF-01A-11R-A33J-07 | 2 | 7.133333 | 2.259452 | DDX20 | Low |
| TCGA-KR-A7K8-01A-11R-A33J-07 | 1 | 30.2 | 2.259821 | DDX20 | Low |
| TCGA-DD-A73F-01A-11R-A32O-07 | 1 | 36.16667 | 2.261821 | DDX20 | Low |
| TCGA-DD-A4NK-01A-11R-A28V-07 | 2 | 40.33333 | 2.269768 | DDX20 | Low |
| TCGA-G3-A25S-01A-11R-A16W-07 | 2 | 13.86667 | 2.270313 | DDX20 | Low |
| TCGA-DD-AADQ-01A-11R-A41C-07 | 1 | 14.53333 | 2.273482 | DDX20 | Low |
| TCGA-DD-AAEH-01A-11R-A41C-07 | 1 | 26.13333 | 2.274453 | DDX20 | Low |
| TCGA-EP-A26S-01A-11R-A16W-07 | 1 | 20.26667 | 2.274795 | DDX20 | Low |
| TCGA-ZP-A9D4-01A-11R-A37K-07 | 1 | 13.16667 | 2.282661 | DDX20 | Low |
| TCGA-UB-A7MD-01A-12R-A352-07 | 2 | 1.733333 | 2.294091 | DDX20 | Low |
| TCGA-G3-A25T-01A-11R-A16W-07 | 1 | 51.76667 | 2.296382 | DDX20 | Low |
| TCGA-T1-A6J8-01A-11R-A32O-07 | 1 | 0.766667 | 2.29783 | DDX20 | Low |
| TCGA-ZS-A9CF-01A-11R-A38B-07 | 1 | 80.4 | 2.29894 | DDX20 | Low |
| TCGA-G3-AAV2-01A-11R-A37K-07 | 1 | 12.4 | 2.308426 | DDX20 | Low |
| TCGA-DD-A4NH-01A-11R-A27V-07 | 1 | 30.56667 | 2.309069 | DDX20 | Low |
| TCGA-FV-A496-01A-11R-A266-07 | 1 | 0.333333 | 2.314741 | DDX20 | Low |
| TCGA-UB-AA0V-01A-11R-A38B-07 | 1 | 10.46667 | 2.319638 | DDX20 | Low |
| TCGA-DD-AADG-01A-11R-A41C-07 | 1 | 38.16667 | 2.320855 | DDX20 | Low |
| TCGA-5R-AA1D-01A-11R-A38B-07 | 1 | 14.96667 | 2.320953 | DDX20 | Low |
| TCGA-BC-A10Q-01A-11R-A131-07 | 2 | 37.83333 | 2.327048 | DDX20 | Low |
| TCGA-ZS-A9CE-01A-11R-A37K-07 | 1 | 41.36667 | 2.337802 | DDX20 | Low |
| TCGA-DD-AAVU-01A-11R-A41C-07 | 1 | 73.4 | 2.343207 | DDX20 | Low |
| TCGA-G3-AAV3-01A-11R-A37K-07 | 1 | 13.73333 | 2.343692 | DDX20 | High |
| TCGA-RC-A6M5-01A-11R-A32O-07 | 1 | 0.5 | 2.346056 | DDX20 | High |
| TCGA-2Y-A9GZ-01A-11R-A39D-07 | 2 | 28.26667 | 2.356382 | DDX20 | High |
| TCGA-CC-A7IE-01A-21R-A38B-07 | 2 | 7.233333 | 2.359105 | DDX20 | High |
| TCGA-KR-A7K0-01A-12R-A33R-07 | 2 | 2.166667 | 2.363144 | DDX20 | High |
| TCGA-DD-AAVX-01A-11R-A41C-07 | 1 | 57.26667 | 2.363683 | DDX20 | High |
| TCGA-ZP-A9D0-01A-11R-A37K-07 | 1 | 36.36667 | 2.366984 | DDX20 | High |
| TCGA-DD-AACN-01A-11R-A41C-07 | 1 | 43.4 | 2.36742 | DDX20 | High |
| TCGA-2Y-A9H8-01A-11R-A39D-07 | 2 | 21.1 | 2.371917 | DDX20 | High |
| TCGA-CC-A9FW-01A-11R-A37K-07 | 1 | 8.266667 | 2.380384 | DDX20 | High |
| TCGA-DD-A1EG-01A-11R-A213-07 | 2 | 45.73333 | 2.380771 | DDX20 | High |
| TCGA-CC-A9FS-01A-11R-A37K-07 | 1 | 7.033333 | 2.381989 | DDX20 | High |
| TCGA-DD-AACF-01A-11R-A41C-07 | 2 | 12.16667 | 2.387686 | DDX20 | High |
| TCGA-DD-AAED-01A-12R-A41C-07 | 1 | 25.43333 | 2.394349 | DDX20 | High |
| TCGA-HP-A5N0-01A-11R-A28V-07 | 2 | 38.23333 | 2.396885 | DDX20 | High |
| TCGA-RC-A6M4-01A-11R-A32O-07 | 1 | 0.733333 | 2.398085 | DDX20 | High |
| TCGA-ED-A627-01A-12R-A311-07 | 1 | 14.1 | 2.400102 | DDX20 | High |
| TCGA-ED-A4XI-01A-11R-A266-07 | 1 | 27.3 | 2.402532 | DDX20 | High |
| TCGA-BC-A10Z-01A-11R-A131-07 | 2 | 1.133333 | 2.413489 | DDX20 | High |
| TCGA-DD-A73B-01A-12R-A32O-07 | 2 | 9.433333 | 2.418765 | DDX20 | High |
| TCGA-RG-A7D4-01A-12R-A33R-07 | 1 | 36.6 | 2.420902 | DDX20 | High |
| TCGA-CC-A5UC-01A-11R-A28V-07 | 2 | 11.56667 | 2.424085 | DDX20 | High |
| TCGA-3K-AAZ8-01A-12R-A39D-07 | 1 | 13.2 | 2.425035 | DDX20 | High |
| TCGA-G3-A5SI-01A-31R-A27V-07 | 2 | 25.6 | 2.427761 | DDX20 | High |
| TCGA-DD-A4NQ-01A-21R-A28V-07 | 2 | 12.43333 | 2.429005 | DDX20 | High |
| TCGA-DD-AACJ-01A-11R-A41C-07 | 1 | 70.06667 | 2.439551 | DDX20 | High |
| TCGA-ED-A7XO-01A-11R-A352-07 | 1 | 14.23333 | 2.440547 | DDX20 | High |
| TCGA-2Y-A9GV-01A-11R-A38B-07 | 2 | 84.4 | 2.444635 | DDX20 | High |
| TCGA-PD-A5DF-01A-11R-A27V-07 | 2 | 21.3 | 2.446993 | DDX20 | High |
| TCGA-DD-AAVP-01A-11R-A41C-07 | 1 | 91.73333 | 2.455857 | DDX20 | High |
| TCGA-DD-A1EB-01A-11R-A131-07 | 1 | 67.23333 | 2.457076 | DDX20 | High |
| TCGA-FV-A2QR-01A-11R-A213-07 | 2 | 19.36667 | 2.465747 | DDX20 | High |
| TCGA-ED-A66Y-01A-11R-A311-07 | 2 | 9.866667 | 2.467973 | DDX20 | High |
| TCGA-FV-A2QQ-01A-11R-A22L-07 | 1 | 24.3 | 2.469606 | DDX20 | High |
| TCGA-2Y-A9H0-01A-11R-A38B-07 | 1 | 122.5 | 2.473852 | DDX20 | High |
| TCGA-ED-A8O6-01A-11R-A36F-07 | 2 | 1.866667 | 2.475012 | DDX20 | High |
| TCGA-DD-AADR-01A-11R-A41C-07 | 1 | 67.6 | 2.480745 | DDX20 | High |
| TCGA-K7-A5RG-01A-11R-A28V-07 | 1 | 17.3 | 2.483274 | DDX20 | High |
| TCGA-G3-A25Y-01A-11R-A16W-07 | 2 | 15.06667 | 2.484703 | DDX20 | High |
| TCGA-LG-A9QC-01A-11R-A37K-07 | 1 | 14.16667 | 2.488079 | DDX20 | High |
| TCGA-DD-A4NO-01A-11R-A28V-07 | 1 | 74.83333 | 2.489691 | DDX20 | High |
| TCGA-CC-A7IH-01A-11R-A33J-07 | 1 | 12.16667 | 2.490134 | DDX20 | High |
| TCGA-DD-A4NN-01A-11R-A28V-07 | 2 | 29.96667 | 2.490272 | DDX20 | High |
| TCGA-DD-AADW-01A-11R-A39D-07 | 1 | 19.56667 | 2.490974 | DDX20 | High |
| TCGA-DD-A73C-01A-12R-A33J-07 | 1 | 23.36667 | 2.492981 | DDX20 | High |
| TCGA-BW-A5NO-01A-11R-A27V-07 | 1 | 0.666667 | 2.499432 | DDX20 | High |
| TCGA-WX-AA44-01A-11R-A39D-07 | 1 | 20.5 | 2.50203 | DDX20 | High |
| TCGA-2Y-A9H7-01A-11R-A39D-07 | 1 | 38.93333 | 2.504166 | DDX20 | High |
| TCGA-DD-AACS-01A-11R-A41C-07 | 1 | 60.13333 | 2.507579 | DDX20 | High |
| TCGA-CC-A7IL-01A-11R-A33R-07 | 2 | 9.266667 | 2.510761 | DDX20 | High |
| TCGA-ED-A459-01A-11R-A266-07 | 1 | 30.33333 | 2.51488 | DDX20 | High |
| TCGA-DD-AADL-01A-11R-A41C-07 | 1 | 21.2 | 2.517319 | DDX20 | High |
| TCGA-2Y-A9HB-01A-11R-A39D-07 | 1 | 8.666667 | 2.518404 | DDX20 | High |
| TCGA-2V-A95S-01A-11R-A37K-07 | 1 |  | 2.523029 | DDX20 | High |
| TCGA-DD-A73D-01A-12R-A32O-07 | 1 | 23.1 | 2.525272 | DDX20 | High |
| TCGA-DD-AAD5-01A-11R-A41C-07 | 1 | 44.83333 | 2.527005 | DDX20 | High |
| TCGA-DD-AAEE-01A-11R-A41C-07 | 1 | 27 | 2.538728 | DDX20 | High |
| TCGA-DD-AAVY-01A-11R-A41C-07 | 1 | 65.66667 | 2.540414 | DDX20 | High |
| TCGA-DD-A4ND-01A-11R-A266-07 | 1 | 91.53333 | 2.550277 | DDX20 | High |
| TCGA-2Y-A9GX-01A-11R-A38B-07 | 1 | 81.4 | 2.55572 | DDX20 | High |
| TCGA-DD-A4NF-01A-11R-A27V-07 | 1 | 31.4 | 2.556398 | DDX20 | High |
| TCGA-ED-A7XP-01A-11R-A352-07 | 1 | 13.33333 | 2.570773 | DDX20 | High |
| TCGA-UB-A7ME-01A-11R-A33J-07 | 1 | 16.2 | 2.571948 | DDX20 | High |
| TCGA-DD-AACX-01A-11R-A41C-07 | 1 | 5.666667 | 2.572475 | DDX20 | High |
| TCGA-DD-AAVV-01A-11R-A41C-07 | 1 | 81.83333 | 2.584694 | DDX20 | High |
| TCGA-MI-A75I-01A-11R-A32O-07 | 1 | 21 | 2.586244 | DDX20 | High |
| TCGA-FV-A3R2-01A-11R-A22L-07 | 2 | 6.466667 | 2.588099 | DDX20 | High |
| TCGA-5C-A9VG-01A-11R-A37K-07 | 1 | 10.93333 | 2.5903 | DDX20 | High |
| TCGA-G3-A5SL-01A-11R-A27V-07 | 1 | 20.7 | 2.605049 | DDX20 | High |
| TCGA-BC-4072-01B-11R-A155-07 | 2 | 49.66667 | 2.610121 | DDX20 | High |
| TCGA-G3-AAV1-01A-11R-A38B-07 | 2 | 11.96667 | 2.610308 | DDX20 | High |
| TCGA-CC-A7IG-01A-11R-A33J-07 | 2 | 9.966667 | 2.610682 | DDX20 | High |
| TCGA-ED-A66X-01A-11R-A311-07 | 1 | 13.53333 | 2.612797 | DDX20 | High |
| TCGA-DD-AAVS-01A-11R-A41C-07 | 1 | 60.76667 | 2.614891 | DDX20 | High |
| TCGA-DD-AACA-01A-11R-A41C-07 | 1 | 76.7 | 2.630971 | DDX20 | High |
| TCGA-ED-A7PX-01A-51R-A352-07 | 1 | 0.2 | 2.632672 | DDX20 | High |
| TCGA-DD-A1EA-01A-11R-A131-07 | 1 | 80.5 | 2.644678 | DDX20 | High |
| TCGA-FV-A3I1-01A-11R-A22L-07 | 2 | 8.233333 | 2.644725 | DDX20 | High |
| TCGA-BD-A3EP-01A-11R-A22L-07 | 1 | 13.63333 | 2.661333 | DDX20 | High |
| TCGA-UB-A7MC-01A-11R-A33R-07 | 1 | 16.66667 | 2.67892 | DDX20 | High |
| TCGA-DD-A1EL-01A-11R-A155-07 | 2 | 13.83333 | 2.683637 | DDX20 | High |
| TCGA-EP-A3RK-01A-11R-A22L-07 | 1 | 12.1 | 2.685648 | DDX20 | High |
| TCGA-DD-A1EC-01A-21R-A131-07 | 1 | 20.06667 | 2.688005 | DDX20 | High |
| TCGA-CC-A9FV-01A-11R-A37K-07 | 1 | 0 | 2.697577 | DDX20 | High |
| TCGA-BW-A5NP-01A-11R-A27V-07 | 1 | 0 | 2.700282 | DDX20 | High |
| TCGA-CC-5262-01A-01R-A131-07 | 2 | 3.433333 | 2.708128 | DDX20 | High |
| TCGA-DD-AAD8-01A-11R-A41C-07 | 1 | 40.63333 | 2.711416 | DDX20 | High |
| TCGA-DD-A4NR-01A-11R-A311-07 | 2 | 0.3 | 2.711762 | DDX20 | High |
| TCGA-DD-AACA-02A-11R-A41C-07 | 1 | 76.7 | 2.717138 | DDX20 | High |
| TCGA-DD-AAD1-01A-11R-A41C-07 | 1 | 18.8 | 2.720857 | DDX20 | High |
| TCGA-DD-A39Y-01A-11R-A213-07 | 2 | 5.7 | 2.723418 | DDX20 | High |
| TCGA-DD-AACZ-01A-11R-A41C-07 | 2 | 5.7 | 2.727084 | DDX20 | High |
| TCGA-DD-A11A-01A-11R-A131-07 | 1 | 2.633333 | 2.731149 | DDX20 | High |
| TCGA-BC-A3KG-01A-11R-A213-07 | 1 | 22.66667 | 2.732546 | DDX20 | High |
| TCGA-ED-A97K-01A-21R-A38B-07 | 1 | 0.2 | 2.736869 | DDX20 | High |
| TCGA-DD-AADV-01A-11R-A39D-07 | 1 | 19.13333 | 2.739654 | DDX20 | High |
| TCGA-XR-A8TG-01A-11R-A36F-07 | 1 | 29.93333 | 2.7496 | DDX20 | High |
| TCGA-G3-A5SJ-01A-11R-A27V-07 | 1 | 23.26667 | 2.755039 | DDX20 | High |
| TCGA-CC-5258-01A-01R-A131-07 | 2 | 4.3 | 2.755794 | DDX20 | High |
| TCGA-DD-A39W-01A-11R-A213-07 | 2 | 27.56667 | 2.756174 | DDX20 | High |
| TCGA-2Y-A9GY-01A-11R-A38B-07 | 2 | 25.23333 | 2.759647 | DDX20 | High |
| TCGA-BC-A10U-01A-11R-A131-07 | 2 | 27.9 | 2.761226 | DDX20 | High |
| TCGA-CC-A8HV-01A-11R-A36F-07 | 2 | 9.3 | 2.765863 | DDX20 | High |
| TCGA-DD-AAW3-01A-11R-A41C-07 | 1 | 54.43333 | 2.76688 | DDX20 | High |
| TCGA-GJ-A9DB-01A-11R-A37K-07 | 2 | 2.233333 | 2.768693 | DDX20 | High |
| TCGA-FV-A23B-01A-11R-A16W-07 | 2 | 61.73333 | 2.769172 | DDX20 | High |
| TCGA-G3-A25U-01A-11R-A16W-07 | 1 | 54.53333 | 2.772088 | DDX20 | High |
| TCGA-DD-AAW0-01A-11R-A41C-07 | 1 | 67.16667 | 2.778469 | DDX20 | High |
| TCGA-DD-AADP-01A-11R-A39D-07 | 1 | 15.26667 | 2.784592 | DDX20 | High |
| TCGA-DD-AADI-01A-11R-A41C-07 | 1 | 36.16667 | 2.793264 | DDX20 | High |
| TCGA-G3-A3CK-01A-11R-A213-07 | 1 | 19.5 | 2.801303 | DDX20 | High |
| TCGA-DD-AAC8-01A-11R-A41C-07 | 2 | 0.533333 | 2.817715 | DDX20 | High |
| TCGA-DD-AAD0-01A-11R-A41C-07 | 1 | 4.566667 | 2.836493 | DDX20 | High |
| TCGA-K7-AAU7-01A-11R-A38B-07 | 1 | 11.96667 | 2.837327 | DDX20 | High |
| TCGA-BC-A10Y-01A-11R-A131-07 | 2 | 23.7 | 2.841011 | DDX20 | High |
| TCGA-DD-A4NS-01A-11R-A311-07 | 2 | 81.86667 | 2.84681 | DDX20 | High |
| TCGA-DD-A4NJ-01A-11R-A27V-07 | 1 | 30.93333 | 2.854416 | DDX20 | High |
| TCGA-G3-A6UC-01A-21R-A33J-07 | 1 | 22.36667 | 2.856246 | DDX20 | High |
| TCGA-4R-AA8I-01A-11R-A38B-07 | 2 | 8.733333 | 2.864109 | DDX20 | High |
| TCGA-DD-AACL-01A-11R-A41C-07 | 2 | 3.566667 | 2.870344 | DDX20 | High |
| TCGA-DD-AADY-01A-11R-A41C-07 | 1 | 18.5 | 2.87112 | DDX20 | High |
| TCGA-G3-AAV4-01A-11R-A38B-07 | 2 | 0.9 | 2.87113 | DDX20 | High |
| TCGA-BC-A217-01A-11R-A155-07 | 2 | 46.56667 | 2.872522 | DDX20 | High |
| TCGA-ZS-A9CF-02A-11R-A38B-07 | 1 | 80.4 | 2.873139 | DDX20 | High |
| TCGA-CC-A8HU-01A-11R-A36F-07 | 2 | 11.46667 | 2.874058 | DDX20 | High |
| TCGA-XR-A8TE-01A-11R-A36F-07 | 1 | 30.83333 | 2.878668 | DDX20 | High |
| TCGA-XR-A8TF-01A-11R-A36F-07 | 2 | 23.1 | 2.897878 | DDX20 | High |
| TCGA-DD-A73E-01A-12R-A32O-07 | 1 | 1.466667 | 2.923929 | DDX20 | High |
| TCGA-BC-A3KF-01A-11R-A213-07 | 1 | 0.266667 | 2.924235 | DDX20 | High |
| TCGA-EP-A12J-01A-11R-A131-07 | 1 | 19 | 2.946757 | DDX20 | High |
| TCGA-DD-AACB-01A-11R-A41C-07 | 1 | 77.46667 | 2.94854 | DDX20 | High |
| TCGA-ZP-A9CV-01A-11R-A38B-07 | 2 | 36.26667 | 2.95214 | DDX20 | High |
| TCGA-2Y-A9HA-01A-11R-A39D-07 | 2 | 1.2 | 2.964942 | DDX20 | High |
| TCGA-UB-A7MA-01A-11R-A33R-07 | 1 | 28.26667 | 2.968877 | DDX20 | High |
| TCGA-DD-A1EF-01A-11R-A131-07 | 2 | 13.13333 | 2.969285 | DDX20 | High |
| TCGA-DD-A4NA-01A-11R-A266-07 | 1 | 33.6 | 2.992686 | DDX20 | High |
| TCGA-ZP-A9CZ-01A-11R-A38B-07 | 1 | 23.53333 | 2.999495 | DDX20 | High |
| TCGA-CC-A8HT-01A-11R-A36F-07 | 2 | 4.666667 | 3.002561 | DDX20 | High |
| TCGA-BC-A216-01A-11R-A155-07 | 1 | 45.03333 | 3.011021 | DDX20 | High |
| TCGA-UB-AA0U-01A-11R-A38B-07 | 1 | 10.9 | 3.026191 | DDX20 | High |
| TCGA-RC-A6M3-01A-11R-A32O-07 | 1 | 0 | 3.030162 | DDX20 | High |
| TCGA-DD-AACK-01A-11R-A41C-07 | 1 | 0.3 | 3.037432 | DDX20 | High |
| TCGA-LG-A6GG-01A-11R-A311-07 | 1 | 12.9 | 3.042882 | DDX20 | High |
| TCGA-DD-AACV-01A-11R-A41C-07 | 1 | 51.03333 | 3.053668 | DDX20 | High |
| TCGA-QA-A7B7-01A-11R-A32O-07 | 1 | 3.133333 | 3.067709 | DDX20 | High |
| TCGA-YA-A8S7-01A-11R-A37K-07 | 2 | 13.73333 | 3.072794 | DDX20 | High |
| TCGA-DD-AADB-01A-11R-A41C-07 | 1 | 41.4 | 3.106071 | DDX20 | High |
| TCGA-CC-A5UD-01A-11R-A28V-07 | 2 | 10.13333 | 3.10762 | DDX20 | High |
| TCGA-CC-5264-01A-01R-A131-07 | 2 | 3.4 | 3.119243 | DDX20 | High |
| TCGA-CC-A3MA-01A-11R-A213-07 | 2 | 10.1 | 3.147421 | DDX20 | High |
| TCGA-CC-A3M9-01A-11R-A213-07 | 2 | 10 | 3.169475 | DDX20 | High |
| TCGA-2Y-A9GS-01A-12R-A38B-07 | 2 | 24.13333 | 3.185269 | DDX20 | High |
| TCGA-DD-AA3A-01A-11R-A37K-07 | 2 | 13.66667 | 3.19443 | DDX20 | High |
| TCGA-DD-A1EE-01A-11R-A131-07 | 2 | 11.63333 | 3.198856 | DDX20 | High |
| TCGA-DD-AADC-01A-11R-A41C-07 | 2 | 14.16667 | 3.201003 | DDX20 | High |
| TCGA-DD-AACP-01A-11R-A41C-07 | 1 | 13.83333 | 3.205938 | DDX20 | High |
| TCGA-BC-4073-01B-02R-A131-07 | 1 | 28.3 | 3.207303 | DDX20 | High |
| TCGA-DD-A1EJ-01A-11R-A155-07 | 2 | 33.5 | 3.230437 | DDX20 | High |
| TCGA-FV-A4ZQ-01A-11R-A266-07 | 1 | 0.4 | 3.232355 | DDX20 | High |
| TCGA-CC-A9FU-01A-11R-A37K-07 | 1 | 0 | 3.232788 | DDX20 | High |
| TCGA-ED-A82E-01A-11R-A352-07 | 1 | 13.6 | 3.266992 | DDX20 | High |
| TCGA-DD-AACH-01A-11R-A41C-07 | 2 | 6.5 | 3.268199 | DDX20 | High |
| TCGA-CC-5263-01A-01R-A131-07 | 2 | 4.3 | 3.309798 | DDX20 | High |
| TCGA-5R-AA1C-01A-11R-A41C-07 | 1 | 17.33333 | 3.318353 | DDX20 | High |
| TCGA-CC-5261-01A-01R-A131-07 | 2 | 3.233333 | 3.319288 | DDX20 | High |
| TCGA-CC-5260-01A-01R-A131-07 | 2 | 2.9 | 3.333274 | DDX20 | High |
| TCGA-DD-AAVZ-01A-11R-A41C-07 | 1 | 63.33333 | 3.333511 | DDX20 | High |
| TCGA-CC-A5UE-01A-11R-A28V-07 | 2 | 9.066667 | 3.394074 | DDX20 | High |
| TCGA-G3-AAV7-01A-11R-A38B-07 | 1 | 12.03333 | 3.394276 | DDX20 | High |
| TCGA-UB-A7MB-01A-11R-A33R-07 | 1 | 20.03333 | 3.397671 | DDX20 | High |
| TCGA-ED-A5KG-01A-11R-A27V-07 | 1 | 28.46667 | 3.412751 | DDX20 | High |
| TCGA-CC-A7IK-01A-12R-A33R-07 | 2 | 8.733333 | 3.455679 | DDX20 | High |
| TCGA-ED-A8O5-01A-11R-A36F-07 | 1 | 13.53333 | 3.466182 | DDX20 | High |
| TCGA-DD-AADD-01A-11R-A41C-07 | 1 | 41.03333 | 3.475616 | DDX20 | High |
| TCGA-BC-A8YO-01A-11R-A37K-07 | 1 | 18.73333 | 3.50942 | DDX20 | High |
| TCGA-2Y-A9H2-01A-12R-A38B-07 | 1 | 57.7 | 3.528609 | DDX20 | High |
| TCGA-DD-A114-01A-11R-A131-07 | 2 | 38.3 | 3.548504 | DDX20 | High |
| TCGA-CC-A7IJ-01A-11R-A33R-07 | 1 | 12.73333 | 3.562011 | DDX20 | High |
| TCGA-CC-A8HS-01A-11R-A36F-07 | 2 | 10 | 3.566582 | DDX20 | High |
| TCGA-CC-A1HT-01A-11R-A131-07 | 2 | 3.366667 | 3.60334 | DDX20 | High |
| TCGA-CC-A7II-01A-11R-A33J-07 | 1 | 13.3 | 3.62753 | DDX20 | High |
| TCGA-BC-A112-01A-11R-A131-07 | 2 | 5.1 | 3.640174 | DDX20 | High |
| TCGA-ZP-A9D2-01A-11R-A38B-07 | 2 | 25.5 | 3.658386 | DDX20 | High |
| TCGA-BW-A5NQ-01A-11R-A27V-07 | 1 | 0 | 3.668985 | DDX20 | High |
| TCGA-G3-A7M9-01A-23R-A352-07 | 2 | 1.866667 | 4.002369 | DDX20 | High |
| TCGA-G3-A7M6-01A-11R-A33R-07 | 1 | 21.06667 | 4.029581 | DDX20 | High |
| TCGA-BC-A10W-01A-11R-A131-07 | 2 | 3.033333 | 4.942232 | DDX20 | High |

**Supplement.5 PFI median value**

| sample_id | event | time | expr | gene | group |
| --- | --- | --- | --- | --- | --- |
| TCGA-DD-AADJ-01A-11R-A41C-07 | 1 | 35.53333 | 1.064212 | DDX20 | Low |
| TCGA-DD-A3A2-01A-11R-A213-07 | 1 | 71.03333 | 1.165264 | DDX20 | Low |
| TCGA-ES-A2HS-01A-11R-A180-07 | 1 | 22.93333 | 1.170712 | DDX20 | Low |
| TCGA-FV-A4ZP-01A-12R-A266-07 | 1 | 82.86667 | 1.25593 | DDX20 | Low |
| TCGA-DD-A3A7-01A-11R-A22L-07 | 2 | 4.8 | 1.2686 | DDX20 | Low |
| TCGA-2Y-A9H1-01A-11R-A38B-07 | 2 | 10.7 | 1.279123 | DDX20 | Low |
| TCGA-DD-AAEB-01A-11R-A41C-07 | 1 | 15.93333 | 1.282654 | DDX20 | Low |
| TCGA-G3-AAV0-01A-11R-A37K-07 | 1 | 15.86667 | 1.295096 | DDX20 | Low |
| TCGA-DD-AADS-01A-11R-A41C-07 | 1 | 15.8 | 1.319698 | DDX20 | Low |
| TCGA-2Y-A9H3-01A-11R-A38B-07 | 2 | 0.733333 | 1.334163 | DDX20 | Low |
| TCGA-CC-5259-01A-31R-A213-07 | 2 | 6 | 1.35142 | DDX20 | Low |
| TCGA-DD-A3A9-01A-11R-A266-07 | 2 | 9.633333 | 1.397461 | DDX20 | Low |
| TCGA-DD-AADO-01A-11R-A41C-07 | 1 | 15.1 | 1.400664 | DDX20 | Low |
| TCGA-ES-A2HT-01A-12R-A180-07 | 1 | 14.6 | 1.43357 | DDX20 | Low |
| TCGA-WQ-AB4B-01A-11R-A41C-07 | 2 | 9.1 | 1.456798 | DDX20 | Low |
| TCGA-RC-A7SH-01A-11R-A38B-07 | 2 | 3.033333 | 1.467304 | DDX20 | Low |
| TCGA-2Y-A9H6-01A-11R-A39D-07 | 1 | 11.9 | 1.496864 | DDX20 | Low |
| TCGA-DD-A3A3-01A-11R-A22L-07 | 1 | 17.83333 | 1.502397 | DDX20 | Low |
| TCGA-2Y-A9GU-01A-11R-A38B-07 | 1 | 64.63333 | 1.510335 | DDX20 | Low |
| TCGA-DD-A73G-01A-22R-A32O-07 | 1 | 115.9333 | 1.52873 | DDX20 | Low |
| TCGA-DD-A39X-01A-11R-A213-07 | 2 | 34.4 | 1.547331 | DDX20 | Low |
| TCGA-NI-A4U2-01A-11R-A28V-07 | 2 | 3.533333 | 1.54919 | DDX20 | Low |
| TCGA-DD-A11C-01A-11R-A131-07 | 1 | 22.06667 | 1.55171 | DDX20 | Low |
| TCGA-BC-A10R-01A-11R-A131-07 | 2 | 6.633333 | 1.556417 | DDX20 | Low |
| TCGA-DD-AAE1-01A-11R-A41C-07 | 1 | 18.4 | 1.561645 | DDX20 | Low |
| TCGA-DD-AADU-01A-11R-A41C-07 | 1 | 18.46667 | 1.584639 | DDX20 | Low |
| TCGA-NI-A8LF-01A-11R-A36F-07 | 1 | 26.63333 | 1.603869 | DDX20 | Low |
| TCGA-DD-AAC9-01A-11R-A41C-07 | 1 | 11.56667 | 1.615012 | DDX20 | Low |
| TCGA-DD-A4NG-01A-11R-A27V-07 | 2 | 14.33333 | 1.626254 | DDX20 | Low |
| TCGA-DD-A119-01A-11R-A131-07 | 1 | 7.433333 | 1.633978 | DDX20 | Low |
| TCGA-DD-A11B-01A-11R-A131-07 | 1 | 0.466667 | 1.635381 | DDX20 | Low |
| TCGA-DD-AADN-01A-11R-A41C-07 | 1 | 29.93333 | 1.638302 | DDX20 | Low |
| TCGA-DD-AADM-01A-11R-A41C-07 | 1 | 0.4 | 1.645864 | DDX20 | Low |
| TCGA-DD-A1EK-01A-11R-A213-07 | 2 | 5.566667 | 1.650498 | DDX20 | Low |
| TCGA-EP-A3JL-01A-11R-A213-07 | 1 | 10.1 | 1.654744 | DDX20 | Low |
| TCGA-G3-A5SM-01A-12R-A28V-07 | 2 | 8.4 | 1.666926 | DDX20 | Low |
| TCGA-2Y-A9GT-01A-11R-A38B-07 | 2 | 36.1 | 1.66704 | DDX20 | Low |
| TCGA-RC-A7SF-01A-11R-A352-07 | 2 | 16.73333 | 1.670233 | DDX20 | Low |
| TCGA-WX-AA47-01A-11R-A39D-07 | 2 | 3.433333 | 1.671176 | DDX20 | Low |
| TCGA-DD-A11D-01A-11R-A131-07 | 2 | 16.6 | 1.673865 | DDX20 | Low |
| TCGA-G3-A7M5-01A-11R-A33R-07 | 1 | 14.9 | 1.678851 | DDX20 | Low |
| TCGA-KR-A7K7-01A-11R-A33J-07 | 2 | 7.966667 | 1.683113 | DDX20 | Low |
| TCGA-DD-A4NP-01A-11R-A28V-07 | 2 | 42.86667 | 1.693381 | DDX20 | Low |
| TCGA-EP-A2KB-01A-11R-A180-07 | 2 | 5.366667 | 1.69444 | DDX20 | Low |
| TCGA-5C-A9VH-01A-11R-A37K-07 | 1 | 10.73333 | 1.712315 | DDX20 | Low |
| TCGA-DD-AAE3-01A-11R-A41C-07 | 1 | 18.86667 | 1.72677 | DDX20 | Low |
| TCGA-DD-A3A6-01A-11R-A22L-07 | 1 | 108.6 | 1.727001 | DDX20 | Low |
| TCGA-LG-A9QD-01A-11R-A38B-07 | 1 | 12.2 | 1.727519 | DDX20 | Low |
| TCGA-G3-A5SK-01A-11R-A27V-07 | 2 | 16.1 | 1.731907 | DDX20 | Low |
| TCGA-DD-A4NL-01A-11R-A28V-07 | 1 | 57.03333 | 1.736204 | DDX20 | Low |
| TCGA-BC-A10S-01A-22R-A131-07 | 2 | 37.66667 | 1.753466 | DDX20 | Low |
| TCGA-G3-AAUZ-01A-11R-A38B-07 | 1 | 16 | 1.760589 | DDX20 | Low |
| TCGA-DD-AACD-01A-11R-A41C-07 | 2 | 5.033333 | 1.762419 | DDX20 | Low |
| TCGA-BC-A110-01A-11R-A131-07 | 2 | 68.56667 | 1.775718 | DDX20 | Low |
| TCGA-RC-A7SB-01A-21R-A352-07 | 1 | 19.6 | 1.783421 | DDX20 | Low |
| TCGA-DD-A39Z-01A-11R-A213-07 | 1 | 20.03333 | 1.794993 | DDX20 | Low |
| TCGA-FV-A3R3-01A-11R-A22L-07 | 1 | 12.2 | 1.80246 | DDX20 | Low |
| TCGA-G3-AAV6-01A-21R-A37K-07 | 2 | 1.466667 | 1.805757 | DDX20 | Low |
| TCGA-DD-AADF-01A-11R-A41C-07 | 1 | 3.833333 | 1.813588 | DDX20 | Low |
| TCGA-DD-AADK-01A-11R-A41C-07 | 1 | 34.96667 | 1.814216 | DDX20 | Low |
| TCGA-G3-A3CJ-01A-11R-A213-07 | 2 | 9.866667 | 1.816197 | DDX20 | Low |
| TCGA-G3-A25Z-01A-11R-A16W-07 | 1 | 21.83333 | 1.819328 | DDX20 | Low |
| TCGA-DD-A4NE-01A-11R-A27V-07 | 2 | 6.333333 | 1.823071 | DDX20 | Low |
| TCGA-K7-A6G5-01A-11R-A311-07 | 1 | 17.06667 | 1.826309 | DDX20 | Low |
| TCGA-G3-A3CI-01A-11R-A213-07 | 1 | 6 | 1.832281 | DDX20 | Low |
| TCGA-CC-A123-01A-11R-A131-07 | 2 | 4.833333 | 1.832514 | DDX20 | Low |
| TCGA-DD-AADA-01A-11R-A41C-07 | 1 | 41.1 | 1.835252 | DDX20 | Low |
| TCGA-DD-AAE4-01A-11R-A41C-07 | 2 | 3.7 | 1.840678 | DDX20 | Low |
| TCGA-DD-A73A-01A-12R-A32O-07 | 1 | 24.26667 | 1.842496 | DDX20 | Low |
| TCGA-HP-A5MZ-01A-21R-A27V-07 | 1 | 3.033333 | 1.842559 | DDX20 | Low |
| TCGA-2Y-A9GW-01A-11R-A38B-07 | 2 | 40.96667 | 1.843917 | DDX20 | Low |
| TCGA-DD-A4NI-01A-11R-A27V-07 | 1 | 27.2 | 1.861462 | DDX20 | Low |
| TCGA-WJ-A86L-01A-12R-A39D-07 | 1 | 11.5 | 1.873321 | DDX20 | Low |
| TCGA-DD-AAE7-01A-11R-A41C-07 | 1 | 21.46667 | 1.880617 | DDX20 | Low |
| TCGA-DD-AAVW-01A-11R-A41C-07 | 1 | 77.23333 | 1.885077 | DDX20 | Low |
| TCGA-DD-AACI-01A-11R-A41C-07 | 2 | 47.73333 | 1.885948 | DDX20 | Low |
| TCGA-EP-A2KC-01A-11R-A213-07 | 1 | 0.633333 | 1.889377 | DDX20 | Low |
| TCGA-DD-A3A8-01A-11R-A22L-07 | 1 | 0.366667 | 1.891978 | DDX20 | Low |
| TCGA-DD-AACE-01A-11R-A41C-07 | 2 | 42.63333 | 1.898557 | DDX20 | Low |
| TCGA-KR-A7K2-01A-12R-A33R-07 | 1 | 27.63333 | 1.898811 | DDX20 | Low |
| TCGA-DD-A39V-01A-11R-A213-07 | 2 | 15.16667 | 1.909601 | DDX20 | Low |
| TCGA-G3-A3CH-01A-11R-A22L-07 | 2 | 3.866667 | 1.910645 | DDX20 | Low |
| TCGA-CC-A7IF-01A-11R-A33J-07 | 2 | 8.466667 | 1.911106 | DDX20 | Low |
| TCGA-MI-A75C-01A-11R-A32O-07 | 1 | 9.7 | 1.919003 | DDX20 | Low |
| TCGA-BC-A69H-01A-11R-A311-07 | 1 | 14.8 | 1.925231 | DDX20 | Low |
| TCGA-K7-A5RF-01A-11R-A28V-07 | 2 | 6.833333 | 1.926422 | DDX20 | Low |
| TCGA-BD-A3ER-01A-11R-A213-07 | 2 | 7.533333 | 1.928197 | DDX20 | Low |
| TCGA-DD-AAEI-01A-11R-A41C-07 | 2 | 16.36667 | 1.929191 | DDX20 | Low |
| TCGA-FV-A495-01A-11R-A266-07 | 1 | 0.033333 | 1.931661 | DDX20 | Low |
| TCGA-DD-AACW-01A-11R-A41C-07 | 1 | 47.46667 | 1.93228 | DDX20 | Low |
| TCGA-DD-AAVR-01A-11R-A41C-07 | 1 | 83.76667 | 1.934729 | DDX20 | Low |
| TCGA-DD-AACT-01A-11R-A41C-07 | 1 | 52.06667 | 1.942671 | DDX20 | Low |
| TCGA-ZS-A9CD-01A-11R-A37K-07 | 2 | 12.36667 | 1.94313 | DDX20 | Low |
| TCGA-DD-AAEG-01A-11R-A39D-07 | 1 | 23.96667 | 1.950771 | DDX20 | Low |
| TCGA-WX-AA46-01A-11R-A39D-07 | 1 | 25.2 | 1.950981 | DDX20 | Low |
| TCGA-DD-AAE9-01A-11R-A41C-07 | 1 | 24.06667 | 1.953674 | DDX20 | Low |
| TCGA-G3-A7M8-01A-11R-A33R-07 | 1 | 14.33333 | 1.958727 | DDX20 | Low |
| TCGA-G3-AAV5-01A-11R-A37K-07 | 2 | 3.9 | 1.960556 | DDX20 | Low |
| TCGA-FV-A3I0-01A-11R-A22L-07 | 2 | 5.3 | 1.970203 | DDX20 | Low |
| TCGA-2Y-A9H4-01A-11R-A38B-07 | 1 | 48.4 | 1.998543 | DDX20 | Low |
| TCGA-DD-AAVQ-01A-11R-A41C-07 | 2 | 50.3 | 2.005929 | DDX20 | Low |
| TCGA-DD-A1ED-01A-11R-A155-07 | 1 | 76.7 | 2.007177 | DDX20 | Low |
| TCGA-5R-AAAM-01A-12R-A41C-07 | 1 | 1.533333 | 2.00738 | DDX20 | Low |
| TCGA-DD-AAD2-01A-11R-A41C-07 | 1 | 21.93333 | 2.014743 | DDX20 | Low |
| TCGA-DD-A115-01A-11R-A131-07 | 2 | 15.63333 | 2.016246 | DDX20 | Low |
| TCGA-MI-A75G-01A-11R-A32O-07 | 1 | 23.26667 | 2.017876 | DDX20 | Low |
| TCGA-DD-A3A4-01A-11R-A22L-07 | 2 | 2.9 | 2.037547 | DDX20 | Low |
| TCGA-DD-AACY-01A-11R-A41C-07 | 2 | 25.86667 | 2.037554 | DDX20 | Low |
| TCGA-RC-A6M6-01A-11R-A32O-07 | 1 | 0.3 | 2.045091 | DDX20 | Low |
| TCGA-MR-A8JO-01A-12R-A36F-07 | 1 | 11 | 2.045342 | DDX20 | Low |
| TCGA-DD-A118-01A-11R-A131-07 | 2 | 21.93333 | 2.04763 | DDX20 | Low |
| TCGA-BD-A2L6-01A-11R-A213-07 | 2 | 13.83333 | 2.049805 | DDX20 | Low |
| TCGA-MR-A520-01A-11R-A266-07 | 1 | 7.633333 | 2.055147 | DDX20 | Low |
| TCGA-CC-A3MB-01A-11R-A213-07 | 2 | 8.666667 | 2.057622 | DDX20 | Low |
| TCGA-5C-AAPD-01A-21R-A39D-07 | 1 | 0.666667 | 2.058116 | DDX20 | Low |
| TCGA-O8-A75V-01A-11R-A32O-07 | 1 | 17.93333 | 2.06549 | DDX20 | Low |
| TCGA-DD-A113-01A-11R-A131-07 | 2 | 55.86667 | 2.06875 | DDX20 | Low |
| TCGA-DD-AAEA-01A-11R-A41C-07 | 1 | 19.16667 | 2.070867 | DDX20 | Low |
| TCGA-DD-A116-01A-11R-A131-07 | 2 | 27.6 | 2.071711 | DDX20 | Low |
| TCGA-BC-A10X-01A-11R-A131-07 | 1 | 25.66667 | 2.07414 | DDX20 | Low |
| TCGA-ED-A7PZ-01A-11R-A33R-07 | 1 | 0.2 | 2.077577 | DDX20 | Low |
| TCGA-BC-A69I-01A-11R-A311-07 | 1 | 12.9 | 2.07806 | DDX20 | Low |
| TCGA-ZS-A9CG-01A-11R-A37K-07 | 1 | 11.36667 | 2.079596 | DDX20 | Low |
| TCGA-DD-AAW1-01A-11R-A41C-07 | 1 | 66.3 | 2.086794 | DDX20 | Low |
| TCGA-MI-A75E-01A-11R-A32O-07 | 1 | 16.9 | 2.090837 | DDX20 | Low |
| TCGA-G3-A7M7-01A-12R-A352-07 | 1 | 12.03333 | 2.097144 | DDX20 | Low |
| TCGA-DD-A4NV-01A-11R-A311-07 | 1 | 79.93333 | 2.098646 | DDX20 | Low |
| TCGA-DD-AAEK-01A-11R-A41C-07 | 1 | 35.56667 | 2.103257 | DDX20 | Low |
| TCGA-CC-A3MC-01A-11R-A22L-07 | 2 | 9.9 | 2.105818 | DDX20 | Low |
| TCGA-2Y-A9H5-01A-11R-A38B-07 | 2 | 8.6 | 2.123046 | DDX20 | Low |
| TCGA-DD-AACA-02B-11R-A41C-07 | 2 | 25.86667 | 2.126794 | DDX20 | Low |
| TCGA-XR-A8TC-01A-11R-A36F-07 | 1 | 44.63333 | 2.130032 | DDX20 | Low |
| TCGA-DD-AACG-01A-11R-A41C-07 | 2 | 4.166667 | 2.131201 | DDX20 | Low |
| TCGA-BC-A10T-01A-11R-A131-07 | 2 | 19.93333 | 2.131625 | DDX20 | Low |
| TCGA-DD-A1EI-01A-11R-A131-07 | 1 | 6.1 | 2.131986 | DDX20 | Low |
| TCGA-DD-AAD6-01A-11R-A41C-07 | 2 | 1.666667 | 2.135104 | DDX20 | Low |
| TCGA-DD-A3A5-01A-11R-A22L-07 | 2 | 2.8 | 2.135551 | DDX20 | Low |
| TCGA-DD-AACO-01A-11R-A41C-07 | 2 | 15.96667 | 2.137256 | DDX20 | Low |
| TCGA-DD-AACU-01A-11R-A41C-07 | 1 | 52.23333 | 2.137677 | DDX20 | Low |
| TCGA-DD-AAE0-01A-11R-A41C-07 | 1 | 18.5 | 2.139354 | DDX20 | Low |
| TCGA-G3-A25V-01A-11R-A16W-07 | 2 | 16.3 | 2.147453 | DDX20 | Low |
| TCGA-DD-AAE6-01A-11R-A41C-07 | 2 | 3.5 | 2.15565 | DDX20 | Low |
| TCGA-XR-A8TD-01A-12R-A39D-07 | 1 | 34.33333 | 2.159063 | DDX20 | Low |
| TCGA-DD-AAD3-01A-11R-A41C-07 | 1 | 43.16667 | 2.166046 | DDX20 | Low |
| TCGA-EP-A2KA-01A-11R-A180-07 | 2 | 9.766667 | 2.169356 | DDX20 | Low |
| TCGA-DD-A1EH-01A-11R-A131-07 | 2 | 4 | 2.17956 | DDX20 | Low |
| TCGA-RC-A7SK-01A-11R-A352-07 | 2 | 10.4 | 2.19201 | DDX20 | Low |
| TCGA-RC-A7S9-01A-11R-A33R-07 | 1 | 21.33333 | 2.192238 | DDX20 | Low |
| TCGA-DD-AACQ-01A-11R-A41C-07 | 2 | 3.5 | 2.19242 | DDX20 | Low |
| TCGA-ZP-A9CY-01A-11R-A38B-07 | 1 | 26.06667 | 2.202614 | DDX20 | Low |
| TCGA-BC-A5W4-01A-11R-A28V-07 | 2 | 12.8 | 2.207802 | DDX20 | Low |
| TCGA-DD-A4NB-01A-12R-A266-07 | 2 | 30.4 | 2.208422 | DDX20 | Low |
| TCGA-DD-A3A1-01A-11R-A213-07 | 1 | 7.766667 | 2.212991 | DDX20 | Low |
| TCGA-G3-A25X-01A-11R-A16W-07 | 1 | 59.3 | 2.214469 | DDX20 | Low |
| TCGA-GJ-A6C0-01A-12R-A311-07 | 1 | 1.033333 | 2.218037 | DDX20 | Low |
| TCGA-MI-A75H-01A-11R-A32O-07 | 2 | 10.03333 | 2.218573 | DDX20 | Low |
| TCGA-G3-A3CG-01A-11R-A213-07 | 2 | 10.96667 | 2.226019 | DDX20 | Low |
| TCGA-ED-A7PY-01A-11R-A33R-07 | 1 | 13 | 2.226853 | DDX20 | Low |
| TCGA-2Y-A9H9-01A-21R-A39D-07 | 2 | 1.966667 | 2.230723 | DDX20 | Low |
| TCGA-WQ-A9G7-01A-11R-A37K-07 | 1 | 1 | 2.23118 | DDX20 | Low |
| TCGA-DD-AAW2-01A-11R-A41C-07 | 2 | 13.03333 | 2.2362 | DDX20 | Low |
| TCGA-DD-AAE2-01A-11R-A41C-07 | 1 | 21.26667 | 2.236835 | DDX20 | Low |
| TCGA-ZP-A9D1-01A-11R-A38B-07 | 1 | 0.7 | 2.254447 | DDX20 | Low |
| TCGA-DD-AACC-01A-11R-A41C-07 | 2 | 17.9 | 2.255589 | DDX20 | Low |
| TCGA-GJ-A3OU-01A-31R-A38B-07 | 2 | 29.3 | 2.258461 | DDX20 | Low |
| TCGA-UB-A7MF-01A-11R-A33J-07 | 2 | 4.433333 | 2.259452 | DDX20 | Low |
| TCGA-KR-A7K8-01A-11R-A33J-07 | 1 | 30.2 | 2.259821 | DDX20 | Low |
| TCGA-DD-A73F-01A-11R-A32O-07 | 1 | 36.16667 | 2.261821 | DDX20 | Low |
| TCGA-DD-A4NK-01A-11R-A28V-07 | 2 | 2.966667 | 2.269768 | DDX20 | Low |
| TCGA-G3-A25S-01A-11R-A16W-07 | 2 | 1.233333 | 2.270313 | DDX20 | Low |
| TCGA-DD-AADQ-01A-11R-A41C-07 | 1 | 14.53333 | 2.273482 | DDX20 | Low |
| TCGA-DD-AAEH-01A-11R-A41C-07 | 2 | 21.46667 | 2.274453 | DDX20 | Low |
| TCGA-EP-A26S-01A-11R-A16W-07 | 1 | 20.26667 | 2.274795 | DDX20 | Low |
| TCGA-ZP-A9D4-01A-11R-A37K-07 | 1 | 13.16667 | 2.282661 | DDX20 | Low |
| TCGA-UB-A7MD-01A-12R-A352-07 | 1 | 1.733333 | 2.294091 | DDX20 | Low |
| TCGA-G3-A25T-01A-11R-A16W-07 | 2 | 15.06667 | 2.296382 | DDX20 | Low |
| TCGA-T1-A6J8-01A-11R-A32O-07 | 1 | 0.766667 | 2.29783 | DDX20 | Low |
| TCGA-ZS-A9CF-01A-11R-A38B-07 | 2 | 21.2 | 2.29894 | DDX20 | Low |
| TCGA-G3-AAV2-01A-11R-A37K-07 | 1 | 12.4 | 2.308426 | DDX20 | Low |
| TCGA-DD-A4NH-01A-11R-A27V-07 | 2 | 5.366667 | 2.309069 | DDX20 | Low |
| TCGA-FV-A496-01A-11R-A266-07 | 1 | 0.333333 | 2.314741 | DDX20 | Low |
| TCGA-UB-AA0V-01A-11R-A38B-07 | 1 | 10.46667 | 2.319638 | DDX20 | Low |
| TCGA-DD-AADG-01A-11R-A41C-07 | 2 | 9 | 2.320855 | DDX20 | Low |
| TCGA-5R-AA1D-01A-11R-A38B-07 | 1 | 14.96667 | 2.320953 | DDX20 | Low |
| TCGA-BC-A10Q-01A-11R-A131-07 | 2 | 11.33333 | 2.327048 | DDX20 | Low |
| TCGA-ZS-A9CE-01A-11R-A37K-07 | 2 | 29.73333 | 2.337802 | DDX20 | Low |
| TCGA-DD-AAVU-01A-11R-A41C-07 | 2 | 54.33333 | 2.343207 | DDX20 | Low |
| TCGA-G3-AAV3-01A-11R-A37K-07 | 1 | 13.73333 | 2.343692 | DDX20 | High |
| TCGA-RC-A6M5-01A-11R-A32O-07 | 1 | 0.5 | 2.346056 | DDX20 | High |
| TCGA-2Y-A9GZ-01A-11R-A39D-07 | 2 | 23.96667 | 2.356382 | DDX20 | High |
| TCGA-CC-A7IE-01A-21R-A38B-07 | 2 | 5.633333 | 2.359105 | DDX20 | High |
| TCGA-KR-A7K0-01A-12R-A33R-07 | 1 | 2.166667 | 2.363144 | DDX20 | High |
| TCGA-DD-AAVX-01A-11R-A41C-07 | 2 | 13.1 | 2.363683 | DDX20 | High |
| TCGA-ZP-A9D0-01A-11R-A37K-07 | 1 | 36.36667 | 2.366984 | DDX20 | High |
| TCGA-DD-AACN-01A-11R-A41C-07 | 2 | 11.9 | 2.36742 | DDX20 | High |
| TCGA-2Y-A9H8-01A-11R-A39D-07 | 2 | 13.26667 | 2.371917 | DDX20 | High |
| TCGA-CC-A9FW-01A-11R-A37K-07 | 2 | 5.1 | 2.380384 | DDX20 | High |
| TCGA-DD-A1EG-01A-11R-A213-07 | 2 | 29.16667 | 2.380771 | DDX20 | High |
| TCGA-CC-A9FS-01A-11R-A37K-07 | 2 | 3.633333 | 2.381989 | DDX20 | High |
| TCGA-DD-AACF-01A-11R-A41C-07 | 2 | 3.966667 | 2.387686 | DDX20 | High |
| TCGA-DD-AAED-01A-12R-A41C-07 | 2 | 6.6 | 2.394349 | DDX20 | High |
| TCGA-HP-A5N0-01A-11R-A28V-07 | 2 | 5.966667 | 2.396885 | DDX20 | High |
| TCGA-RC-A6M4-01A-11R-A32O-07 | 1 | 0.733333 | 2.398085 | DDX20 | High |
| TCGA-ED-A627-01A-12R-A311-07 | 1 | 14.1 | 2.400102 | DDX20 | High |
| TCGA-ED-A4XI-01A-11R-A266-07 | 1 | 27.3 | 2.402532 | DDX20 | High |
| TCGA-BC-A10Z-01A-11R-A131-07 | 1 | 1.133333 | 2.413489 | DDX20 | High |
| TCGA-DD-A73B-01A-12R-A32O-07 | 2 | 8 | 2.418765 | DDX20 | High |
| TCGA-RG-A7D4-01A-12R-A33R-07 | 1 | 36.6 | 2.420902 | DDX20 | High |
| TCGA-CC-A5UC-01A-11R-A28V-07 | 2 | 10.4 | 2.424085 | DDX20 | High |
| TCGA-3K-AAZ8-01A-12R-A39D-07 | 2 | 5.033333 | 2.425035 | DDX20 | High |
| TCGA-G3-A5SI-01A-31R-A27V-07 | 2 | 11.6 | 2.427761 | DDX20 | High |
| TCGA-DD-A4NQ-01A-21R-A28V-07 | 2 | 4.2 | 2.429005 | DDX20 | High |
| TCGA-DD-AACJ-01A-11R-A41C-07 | 2 | 3.333333 | 2.439551 | DDX20 | High |
| TCGA-ED-A7XO-01A-11R-A352-07 | 2 | 8.733333 | 2.440547 | DDX20 | High |
| TCGA-2Y-A9GV-01A-11R-A38B-07 | 2 | 58.16667 | 2.444635 | DDX20 | High |
| TCGA-PD-A5DF-01A-11R-A27V-07 | 2 | 21.3 | 2.446993 | DDX20 | High |
| TCGA-DD-AAVP-01A-11R-A41C-07 | 2 | 30.1 | 2.455857 | DDX20 | High |
| TCGA-DD-A1EB-01A-11R-A131-07 | 2 | 16.83333 | 2.457076 | DDX20 | High |
| TCGA-FV-A2QR-01A-11R-A213-07 | 2 | 18.7 | 2.465747 | DDX20 | High |
| TCGA-ED-A66Y-01A-11R-A311-07 | 1 | 9.866667 | 2.467973 | DDX20 | High |
| TCGA-FV-A2QQ-01A-11R-A22L-07 | 1 | 24.3 | 2.469606 | DDX20 | High |
| TCGA-2Y-A9H0-01A-11R-A38B-07 | 1 | 122.5 | 2.473852 | DDX20 | High |
| TCGA-ED-A8O6-01A-11R-A36F-07 | 1 | 1.866667 | 2.475012 | DDX20 | High |
| TCGA-DD-AADR-01A-11R-A41C-07 | 2 | 56.66667 | 2.480745 | DDX20 | High |
| TCGA-K7-A5RG-01A-11R-A28V-07 | 1 | 17.3 | 2.483274 | DDX20 | High |
| TCGA-G3-A25Y-01A-11R-A16W-07 | 2 | 11.83333 | 2.484703 | DDX20 | High |
| TCGA-LG-A9QC-01A-11R-A37K-07 | 1 | 14.16667 | 2.488079 | DDX20 | High |
| TCGA-DD-A4NO-01A-11R-A28V-07 | 2 | 33 | 2.489691 | DDX20 | High |
| TCGA-CC-A7IH-01A-11R-A33J-07 | 1 | 12.16667 | 2.490134 | DDX20 | High |
| TCGA-DD-A4NN-01A-11R-A28V-07 | 2 | 5.533333 | 2.490272 | DDX20 | High |
| TCGA-DD-AADW-01A-11R-A39D-07 | 2 | 12.86667 | 2.490974 | DDX20 | High |
| TCGA-DD-A73C-01A-12R-A33J-07 | 2 | 18.86667 | 2.492981 | DDX20 | High |
| TCGA-BW-A5NO-01A-11R-A27V-07 | 1 | 0.666667 | 2.499432 | DDX20 | High |
| TCGA-WX-AA44-01A-11R-A39D-07 | 2 | 8.2 | 2.50203 | DDX20 | High |
| TCGA-2Y-A9H7-01A-11R-A39D-07 | 2 | 37.23333 | 2.504166 | DDX20 | High |
| TCGA-DD-AACS-01A-11R-A41C-07 | 1 | 60.13333 | 2.507579 | DDX20 | High |
| TCGA-CC-A7IL-01A-11R-A33R-07 | 2 | 5.966667 | 2.510761 | DDX20 | High |
| TCGA-ED-A459-01A-11R-A266-07 | 1 | 30.33333 | 2.51488 | DDX20 | High |
| TCGA-DD-AADL-01A-11R-A41C-07 | 1 | 21.2 | 2.517319 | DDX20 | High |
| TCGA-2Y-A9HB-01A-11R-A39D-07 | 1 | 8.666667 | 2.518404 | DDX20 | High |
| TCGA-2V-A95S-01A-11R-A37K-07 | 1 |  | 2.523029 | DDX20 | High |
| TCGA-DD-A73D-01A-12R-A32O-07 | 2 | 19.73333 | 2.525272 | DDX20 | High |
| TCGA-DD-AAD5-01A-11R-A41C-07 | 2 | 4.333333 | 2.527005 | DDX20 | High |
| TCGA-DD-AAEE-01A-11R-A41C-07 | 2 | 7.466667 | 2.538728 | DDX20 | High |
| TCGA-DD-AAVY-01A-11R-A41C-07 | 1 | 65.66667 | 2.540414 | DDX20 | High |
| TCGA-DD-A4ND-01A-11R-A266-07 | 2 | 0.5 | 2.550277 | DDX20 | High |
| TCGA-2Y-A9GX-01A-11R-A38B-07 | 2 | 71.1 | 2.55572 | DDX20 | High |
| TCGA-DD-A4NF-01A-11R-A27V-07 | 2 | 21.86667 | 2.556398 | DDX20 | High |
| TCGA-ED-A7XP-01A-11R-A352-07 | 2 | 6.233333 | 2.570773 | DDX20 | High |
| TCGA-UB-A7ME-01A-11R-A33J-07 | 1 | 16.2 | 2.571948 | DDX20 | High |
| TCGA-DD-AACX-01A-11R-A41C-07 | 2 | 1.4 | 2.572475 | DDX20 | High |
| TCGA-DD-AAVV-01A-11R-A41C-07 | 1 | 81.83333 | 2.584694 | DDX20 | High |
| TCGA-MI-A75I-01A-11R-A32O-07 | 2 | 8.833333 | 2.586244 | DDX20 | High |
| TCGA-FV-A3R2-01A-11R-A22L-07 | 1 | 6.466667 | 2.588099 | DDX20 | High |
| TCGA-5C-A9VG-01A-11R-A37K-07 | 1 | 10.93333 | 2.5903 | DDX20 | High |
| TCGA-G3-A5SL-01A-11R-A27V-07 | 1 | 20.7 | 2.605049 | DDX20 | High |
| TCGA-BC-4072-01B-11R-A155-07 | 2 | 15.83333 | 2.610121 | DDX20 | High |
| TCGA-G3-AAV1-01A-11R-A38B-07 | 2 | 1.633333 | 2.610308 | DDX20 | High |
| TCGA-CC-A7IG-01A-11R-A33J-07 | 2 | 6.5 | 2.610682 | DDX20 | High |
| TCGA-ED-A66X-01A-11R-A311-07 | 2 | 4.133333 | 2.612797 | DDX20 | High |
| TCGA-DD-AAVS-01A-11R-A41C-07 | 1 | 60.76667 | 2.614891 | DDX20 | High |
| TCGA-DD-AACA-01A-11R-A41C-07 | 2 | 25.86667 | 2.630971 | DDX20 | High |
| TCGA-ED-A7PX-01A-51R-A352-07 | 1 | 0.2 | 2.632672 | DDX20 | High |
| TCGA-DD-A1EA-01A-11R-A131-07 | 2 | 25.13333 | 2.644678 | DDX20 | High |
| TCGA-FV-A3I1-01A-11R-A22L-07 | 1 | 8.233333 | 2.644725 | DDX20 | High |
| TCGA-BD-A3EP-01A-11R-A22L-07 | 1 | 13.63333 | 2.661333 | DDX20 | High |
| TCGA-UB-A7MC-01A-11R-A33R-07 | 2 | 11.96667 | 2.67892 | DDX20 | High |
| TCGA-DD-A1EL-01A-11R-A155-07 | 2 | 13.83333 | 2.683637 | DDX20 | High |
| TCGA-EP-A3RK-01A-11R-A22L-07 | 2 | 10.66667 | 2.685648 | DDX20 | High |
| TCGA-DD-A1EC-01A-21R-A131-07 | 2 | 9.966667 | 2.688005 | DDX20 | High |
| TCGA-CC-A9FV-01A-11R-A37K-07 | 1 | 0 | 2.697577 | DDX20 | High |
| TCGA-BW-A5NP-01A-11R-A27V-07 | 2 | 3.4 | 2.700282 | DDX20 | High |
| TCGA-CC-5262-01A-01R-A131-07 | 1 | 3.433333 | 2.708128 | DDX20 | High |
| TCGA-DD-AAD8-01A-11R-A41C-07 | 1 | 40.63333 | 2.711416 | DDX20 | High |
| TCGA-DD-A4NR-01A-11R-A311-07 | 1 | 0.3 | 2.711762 | DDX20 | High |
| TCGA-DD-AACA-02A-11R-A41C-07 | 2 | 25.86667 | 2.717138 | DDX20 | High |
| TCGA-DD-AAD1-01A-11R-A41C-07 | 2 | 4.233333 | 2.720857 | DDX20 | High |
| TCGA-DD-A39Y-01A-11R-A213-07 | 1 | 5.7 | 2.723418 | DDX20 | High |
| TCGA-DD-AACZ-01A-11R-A41C-07 | 2 | 2.933333 | 2.727084 | DDX20 | High |
| TCGA-DD-A11A-01A-11R-A131-07 | 1 | 2.633333 | 2.731149 | DDX20 | High |
| TCGA-BC-A3KG-01A-11R-A213-07 | 2 | 6.966667 | 2.732546 | DDX20 | High |
| TCGA-ED-A97K-01A-21R-A38B-07 | 1 | 0.2 | 2.736869 | DDX20 | High |
| TCGA-DD-AADV-01A-11R-A39D-07 | 1 | 19.13333 | 2.739654 | DDX20 | High |
| TCGA-XR-A8TG-01A-11R-A36F-07 | 2 | 11.66667 | 2.7496 | DDX20 | High |
| TCGA-G3-A5SJ-01A-11R-A27V-07 | 2 | 10.26667 | 2.755039 | DDX20 | High |
| TCGA-CC-5258-01A-01R-A131-07 | 1 | 4.3 | 2.755794 | DDX20 | High |
| TCGA-DD-A39W-01A-11R-A213-07 | 1 | 27.56667 | 2.756174 | DDX20 | High |
| TCGA-2Y-A9GY-01A-11R-A38B-07 | 2 | 21.23333 | 2.759647 | DDX20 | High |
| TCGA-BC-A10U-01A-11R-A131-07 | 2 | 19.53333 | 2.761226 | DDX20 | High |
| TCGA-CC-A8HV-01A-11R-A36F-07 | 2 | 4.966667 | 2.765863 | DDX20 | High |
| TCGA-DD-AAW3-01A-11R-A41C-07 | 1 | 54.43333 | 2.76688 | DDX20 | High |
| TCGA-GJ-A9DB-01A-11R-A37K-07 | 1 | 2.233333 | 2.768693 | DDX20 | High |
| TCGA-FV-A23B-01A-11R-A16W-07 | 2 | 48.43333 | 2.769172 | DDX20 | High |
| TCGA-G3-A25U-01A-11R-A16W-07 | 1 | 54.53333 | 2.772088 | DDX20 | High |
| TCGA-DD-AAW0-01A-11R-A41C-07 | 1 | 67.16667 | 2.778469 | DDX20 | High |
| TCGA-DD-AADP-01A-11R-A39D-07 | 1 | 15.26667 | 2.784592 | DDX20 | High |
| TCGA-DD-AADI-01A-11R-A41C-07 | 1 | 36.16667 | 2.793264 | DDX20 | High |
| TCGA-G3-A3CK-01A-11R-A213-07 | 1 | 19.5 | 2.801303 | DDX20 | High |
| TCGA-DD-AAC8-01A-11R-A41C-07 | 1 | 0.533333 | 2.817715 | DDX20 | High |
| TCGA-DD-AAD0-01A-11R-A41C-07 | 2 | 1.566667 | 2.836493 | DDX20 | High |
| TCGA-K7-AAU7-01A-11R-A38B-07 | 2 | 1.933333 | 2.837327 | DDX20 | High |
| TCGA-BC-A10Y-01A-11R-A131-07 | 2 | 13.33333 | 2.841011 | DDX20 | High |
| TCGA-DD-A4NS-01A-11R-A311-07 | 2 | 29.76667 | 2.84681 | DDX20 | High |
| TCGA-DD-A4NJ-01A-11R-A27V-07 | 2 | 18.3 | 2.854416 | DDX20 | High |
| TCGA-G3-A6UC-01A-21R-A33J-07 | 2 | 21.3 | 2.856246 | DDX20 | High |
| TCGA-4R-AA8I-01A-11R-A38B-07 | 2 | 5.266667 | 2.864109 | DDX20 | High |
| TCGA-DD-AACL-01A-11R-A41C-07 | 1 | 3.566667 | 2.870344 | DDX20 | High |
| TCGA-DD-AADY-01A-11R-A41C-07 | 1 | 18.5 | 2.87112 | DDX20 | High |
| TCGA-G3-AAV4-01A-11R-A38B-07 | 1 | 0.9 | 2.87113 | DDX20 | High |
| TCGA-BC-A217-01A-11R-A155-07 | 1 | 46.56667 | 2.872522 | DDX20 | High |
| TCGA-ZS-A9CF-02A-11R-A38B-07 | 2 | 21.2 | 2.873139 | DDX20 | High |
| TCGA-CC-A8HU-01A-11R-A36F-07 | 2 | 10.03333 | 2.874058 | DDX20 | High |
| TCGA-XR-A8TE-01A-11R-A36F-07 | 2 | 7 | 2.878668 | DDX20 | High |
| TCGA-XR-A8TF-01A-11R-A36F-07 | 2 | 7.7 | 2.897878 | DDX20 | High |
| TCGA-DD-A73E-01A-12R-A32O-07 | 1 | 1.466667 | 2.923929 | DDX20 | High |
| TCGA-BC-A3KF-01A-11R-A213-07 | 1 | 0.266667 | 2.924235 | DDX20 | High |
| TCGA-EP-A12J-01A-11R-A131-07 | 1 | 19 | 2.946757 | DDX20 | High |
| TCGA-DD-AACB-01A-11R-A41C-07 | 1 | 77.46667 | 2.94854 | DDX20 | High |
| TCGA-ZP-A9CV-01A-11R-A38B-07 | 2 | 36.26667 | 2.95214 | DDX20 | High |
| TCGA-2Y-A9HA-01A-11R-A39D-07 | 2 | 1.2 | 2.964942 | DDX20 | High |
| TCGA-UB-A7MA-01A-11R-A33R-07 | 2 | 7.366667 | 2.968877 | DDX20 | High |
| TCGA-DD-A1EF-01A-11R-A131-07 | 2 | 13.13333 | 2.969285 | DDX20 | High |
| TCGA-DD-A4NA-01A-11R-A266-07 | 1 | 33.6 | 2.992686 | DDX20 | High |
| TCGA-ZP-A9CZ-01A-11R-A38B-07 | 1 | 23.53333 | 2.999495 | DDX20 | High |
| TCGA-CC-A8HT-01A-11R-A36F-07 | 2 | 2.766667 | 3.002561 | DDX20 | High |
| TCGA-BC-A216-01A-11R-A155-07 | 1 | 45.03333 | 3.011021 | DDX20 | High |
| TCGA-UB-AA0U-01A-11R-A38B-07 | 2 | 3.533333 | 3.026191 | DDX20 | High |
| TCGA-RC-A6M3-01A-11R-A32O-07 | 1 | 0 | 3.030162 | DDX20 | High |
| TCGA-DD-AACK-01A-11R-A41C-07 | 1 | 0.3 | 3.037432 | DDX20 | High |
| TCGA-LG-A6GG-01A-11R-A311-07 | 1 | 12.9 | 3.042882 | DDX20 | High |
| TCGA-DD-AACV-01A-11R-A41C-07 | 2 | 11.46667 | 3.053668 | DDX20 | High |
| TCGA-QA-A7B7-01A-11R-A32O-07 | 2 | 2.933333 | 3.067709 | DDX20 | High |
| TCGA-YA-A8S7-01A-11R-A37K-07 | 2 | 13.26667 | 3.072794 | DDX20 | High |
| TCGA-DD-AADB-01A-11R-A41C-07 | 1 | 41.4 | 3.106071 | DDX20 | High |
| TCGA-CC-A5UD-01A-11R-A28V-07 | 2 | 6.066667 | 3.10762 | DDX20 | High |
| TCGA-CC-5264-01A-01R-A131-07 | 1 | 3.4 | 3.119243 | DDX20 | High |
| TCGA-CC-A3MA-01A-11R-A213-07 | 2 | 8.7 | 3.147421 | DDX20 | High |
| TCGA-CC-A3M9-01A-11R-A213-07 | 2 | 7.3 | 3.169475 | DDX20 | High |
| TCGA-2Y-A9GS-01A-12R-A38B-07 | 2 | 3.4 | 3.185269 | DDX20 | High |
| TCGA-DD-AA3A-01A-11R-A37K-07 | 1 | 13.66667 | 3.19443 | DDX20 | High |
| TCGA-DD-A1EE-01A-11R-A131-07 | 2 | 1.466667 | 3.198856 | DDX20 | High |
| TCGA-DD-AADC-01A-11R-A41C-07 | 2 | 3.366667 | 3.201003 | DDX20 | High |
| TCGA-DD-AACP-01A-11R-A41C-07 | 1 | 13.83333 | 3.205938 | DDX20 | High |
| TCGA-BC-4073-01B-02R-A131-07 | 1 | 28.3 | 3.207303 | DDX20 | High |
| TCGA-DD-A1EJ-01A-11R-A155-07 | 2 | 8.766667 | 3.230437 | DDX20 | High |
| TCGA-FV-A4ZQ-01A-11R-A266-07 | 1 | 0.4 | 3.232355 | DDX20 | High |
| TCGA-CC-A9FU-01A-11R-A37K-07 | 1 | 0 | 3.232788 | DDX20 | High |
| TCGA-ED-A82E-01A-11R-A352-07 | 1 | 13.6 | 3.266992 | DDX20 | High |
| TCGA-DD-AACH-01A-11R-A41C-07 | 2 | 2.766667 | 3.268199 | DDX20 | High |
| TCGA-CC-5263-01A-01R-A131-07 | 1 | 4.3 | 3.309798 | DDX20 | High |
| TCGA-5R-AA1C-01A-11R-A41C-07 | 1 | 17.33333 | 3.318353 | DDX20 | High |
| TCGA-CC-5261-01A-01R-A131-07 | 1 | 3.233333 | 3.319288 | DDX20 | High |
| TCGA-CC-5260-01A-01R-A131-07 | 1 | 2.9 | 3.333274 | DDX20 | High |
| TCGA-DD-AAVZ-01A-11R-A41C-07 | 1 | 63.33333 | 3.333511 | DDX20 | High |
| TCGA-CC-A5UE-01A-11R-A28V-07 | 2 | 5.833333 | 3.394074 | DDX20 | High |
| TCGA-G3-AAV7-01A-11R-A38B-07 | 1 | 12.03333 | 3.394276 | DDX20 | High |
| TCGA-UB-A7MB-01A-11R-A33R-07 | 2 | 1.6 | 3.397671 | DDX20 | High |
| TCGA-ED-A5KG-01A-11R-A27V-07 | 2 | 3.666667 | 3.412751 | DDX20 | High |
| TCGA-CC-A7IK-01A-12R-A33R-07 | 2 | 5 | 3.455679 | DDX20 | High |
| TCGA-ED-A8O5-01A-11R-A36F-07 | 1 | 13.53333 | 3.466182 | DDX20 | High |
| TCGA-DD-AADD-01A-11R-A41C-07 | 1 | 41.03333 | 3.475616 | DDX20 | High |
| TCGA-BC-A8YO-01A-11R-A37K-07 | 2 | 7.333333 | 3.50942 | DDX20 | High |
| TCGA-2Y-A9H2-01A-12R-A38B-07 | 1 | 57.7 | 3.528609 | DDX20 | High |
| TCGA-DD-A114-01A-11R-A131-07 | 1 | 38.3 | 3.548504 | DDX20 | High |
| TCGA-CC-A7IJ-01A-11R-A33R-07 | 1 | 12.73333 | 3.562011 | DDX20 | High |
| TCGA-CC-A8HS-01A-11R-A36F-07 | 2 | 6.7 | 3.566582 | DDX20 | High |
| TCGA-CC-A1HT-01A-11R-A131-07 | 1 | 3.366667 | 3.60334 | DDX20 | High |
| TCGA-CC-A7II-01A-11R-A33J-07 | 2 | 9.266667 | 3.62753 | DDX20 | High |
| TCGA-BC-A112-01A-11R-A131-07 | 1 | 5.1 | 3.640174 | DDX20 | High |
| TCGA-ZP-A9D2-01A-11R-A38B-07 | 2 | 10.5 | 3.658386 | DDX20 | High |
| TCGA-BW-A5NQ-01A-11R-A27V-07 | 1 | 0 | 3.668985 | DDX20 | High |
| TCGA-G3-A7M9-01A-23R-A352-07 | 1 | 1.866667 | 4.002369 | DDX20 | High |
| TCGA-G3-A7M6-01A-11R-A33R-07 | 2 | 9.766667 | 4.029581 | DDX20 | High |
| TCGA-BC-A10W-01A-11R-A131-07 | 2 | 3.033333 | 4.942232 | DDX20 | High |

**Supplement. 6 DSS median value**

| sample_id | event | time | expr | gene | group |
| --- | --- | --- | --- | --- | --- |
| TCGA-DD-AADJ-01A-11R-A41C-07 | 1 | 35.53333 | 1.064212 | DDX20 | Low |
| TCGA-DD-A3A2-01A-11R-A213-07 | 1 | 71.03333 | 1.165264 | DDX20 | Low |
| TCGA-ES-A2HS-01A-11R-A180-07 | 1 | 22.93333 | 1.170712 | DDX20 | Low |
| TCGA-FV-A4ZP-01A-12R-A266-07 | 1 | 82.86667 | 1.25593 | DDX20 | Low |
| TCGA-DD-A3A7-01A-11R-A22L-07 | 2 | 13.96667 | 1.2686 | DDX20 | Low |
| TCGA-2Y-A9H1-01A-11R-A38B-07 | 2 | 40.96667 | 1.279123 | DDX20 | Low |
| TCGA-DD-AAEB-01A-11R-A41C-07 | 1 | 15.93333 | 1.282654 | DDX20 | Low |
| TCGA-G3-AAV0-01A-11R-A37K-07 | 1 | 15.86667 | 1.295096 | DDX20 | Low |
| TCGA-DD-AADS-01A-11R-A41C-07 | 1 | 15.8 | 1.319698 | DDX20 | Low |
| TCGA-2Y-A9H3-01A-11R-A38B-07 | 1 | 50.53333 | 1.334163 | DDX20 | Low |
| TCGA-CC-5259-01A-31R-A213-07 | 1 | 8.333333 | 1.35142 | DDX20 | Low |
| TCGA-DD-A3A9-01A-11R-A266-07 | 2 | 31.03333 | 1.397461 | DDX20 | Low |
| TCGA-DD-AADO-01A-11R-A41C-07 | 1 | 15.1 | 1.400664 | DDX20 | Low |
| TCGA-ES-A2HT-01A-12R-A180-07 | 1 | 14.6 | 1.43357 | DDX20 | Low |
| TCGA-WQ-AB4B-01A-11R-A41C-07 | 1 | 13.16667 | 1.456798 | DDX20 | Low |
| TCGA-RC-A7SH-01A-11R-A38B-07 | 1 | 15.6 | 1.467304 | DDX20 | Low |
| TCGA-2Y-A9H6-01A-11R-A39D-07 | 1 | 11.9 | 1.496864 | DDX20 | Low |
| TCGA-DD-A3A3-01A-11R-A22L-07 | | 17.83333 | 1.502397 | DDX20 | Low |
| TCGA-2Y-A9GU-01A-11R-A38B-07 | 1 | 64.63333 | 1.510335 | DDX20 | Low |
| TCGA-DD-A73G-01A-22R-A32O-07 | 1 | 115.9333 | 1.52873 | DDX20 | Low |
| TCGA-DD-A39X-01A-11R-A213-07 | 2 | 56.46667 | 1.547331 | DDX20 | Low |
| TCGA-NI-A4U2-01A-11R-A28V-07 | 2 | 59.7 | 1.54919 | DDX20 | Low |
| TCGA-DD-A11C-01A-11R-A131-07 | 1 | 22.06667 | 1.55171 | DDX20 | Low |
| TCGA-BC-A10R-01A-11R-A131-07 | 2 | 10.26667 | 1.556417 | DDX20 | Low |
| TCGA-DD-AAE1-01A-11R-A41C-07 | 1 | 18.4 | 1.561645 | DDX20 | Low |
| TCGA-DD-AADU-01A-11R-A41C-07 | 1 | 18.46667 | 1.584639 | DDX20 | Low |
| TCGA-NI-A8LF-01A-11R-A36F-07 | 1 | 26.63333 | 1.603869 | DDX20 | Low |
| TCGA-DD-AAC9-01A-11R-A41C-07 | 1 | 11.56667 | 1.615012 | DDX20 | Low |
| TCGA-DD-A4NG-01A-11R-A27V-07 | 2 | 26.73333 | 1.626254 | DDX20 | Low |
| TCGA-DD-A119-01A-11R-A131-07 | | 7.433333 | 1.633978 | DDX20 | Low |
| TCGA-DD-A11B-01A-11R-A131-07 | 1 | 0.466667 | 1.635381 | DDX20 | Low |
| TCGA-DD-AADN-01A-11R-A41C-07 | 1 | 29.93333 | 1.638302 | DDX20 | Low |
| TCGA-DD-AADM-01A-11R-A41C-07 | 1 | 0.4 | 1.645864 | DDX20 | Low |
| TCGA-DD-A1EK-01A-11R-A213-07 | 2 | 18.6 | 1.650498 | DDX20 | Low |
| TCGA-EP-A3JL-01A-11R-A213-07 | 1 | 10.1 | 1.654744 | DDX20 | Low |
| TCGA-G3-A5SM-01A-12R-A28V-07 | 1 | 17.33333 | 1.666926 | DDX20 | Low |
| TCGA-2Y-A9GT-01A-11R-A38B-07 | 2 | 54.13333 | 1.66704 | DDX20 | Low |
| TCGA-RC-A7SF-01A-11R-A352-07 | 1 | 19.3 | 1.670233 | DDX20 | Low |
| TCGA-WX-AA47-01A-11R-A39D-07 | 2 | 18.53333 | 1.671176 | DDX20 | Low |
| TCGA-DD-A11D-01A-11R-A131-07 | 2 | 52 | 1.673865 | DDX20 | Low |
| TCGA-G3-A7M5-01A-11R-A33R-07 | 1 | 14.9 | 1.678851 | DDX20 | Low |
| TCGA-KR-A7K7-01A-11R-A33J-07 | 1 | 31.7 | 1.683113 | DDX20 | Low |
| TCGA-DD-A4NP-01A-11R-A28V-07 | 1 | 110.2667 | 1.693381 | DDX20 | Low |
| TCGA-EP-A2KB-01A-11R-A180-07 | 2 | 19.86667 | 1.69444 | DDX20 | Low |
| TCGA-5C-A9VH-01A-11R-A37K-07 | 1 | 10.73333 | 1.712315 | DDX20 | Low |
| TCGA-DD-AAE3-01A-11R-A41C-07 | 1 | 18.86667 | 1.72677 | DDX20 | Low |
| TCGA-DD-A3A6-01A-11R-A22L-07 | 1 | 108.6 | 1.727001 | DDX20 | Low |
| TCGA-LG-A9QD-01A-11R-A38B-07 | 1 | 12.2 | 1.727519 | DDX20 | Low |
| TCGA-G3-A5SK-01A-11R-A27V-07 | 1 | 24.8 | 1.731907 | DDX20 | Low |
| TCGA-DD-A4NL-01A-11R-A28V-07 | 1 | 57.03333 | 1.736204 | DDX20 | Low |
| TCGA-BC-A10S-01A-22R-A131-07 | 2 | 47.43333 | 1.753466 | DDX20 | Low |
| TCGA-G3-AAUZ-01A-11R-A38B-07 | 1 | 16 | 1.760589 | DDX20 | Low |
| TCGA-DD-AACD-01A-11R-A41C-07 | 2 | 12.7 | 1.762419 | DDX20 | Low |
| TCGA-BC-A110-01A-11R-A131-07 | 2 | 70.53333 | 1.775718 | DDX20 | Low |
| TCGA-RC-A7SB-01A-21R-A352-07 | 1 | 19.6 | 1.783421 | DDX20 | Low |
| TCGA-DD-A39Z-01A-11R-A213-07 | 1 | 20.03333 | 1.794993 | DDX20 | Low |
| TCGA-FV-A3R3-01A-11R-A22L-07 | 1 | 12.2 | 1.80246 | DDX20 | Low |
| TCGA-G3-AAV6-01A-21R-A37K-07 | 2 | 2.166667 | 1.805757 | DDX20 | Low |
| TCGA-DD-AADF-01A-11R-A41C-07 | 1 | 3.833333 | 1.813588 | DDX20 | Low |
| TCGA-DD-AADK-01A-11R-A41C-07 | 1 | 34.96667 | 1.814216 | DDX20 | Low |
| TCGA-G3-A3CJ-01A-11R-A213-07 | 1 | 19.8 | 1.816197 | DDX20 | Low |
| TCGA-G3-A25Z-01A-11R-A16W-07 | 1 | 21.83333 | 1.819328 | DDX20 | Low |
| TCGA-DD-A4NE-01A-11R-A27V-07 | 2 | 22 | 1.823071 | DDX20 | Low |
| TCGA-K7-A6G5-01A-11R-A311-07 | 1 | 17.06667 | 1.826309 | DDX20 | Low |
| TCGA-G3-A3CI-01A-11R-A213-07 | 1 | 6 | 1.832281 | DDX20 | Low |
| TCGA-CC-A123-01A-11R-A131-07 | 1 | 7.3 | 1.832514 | DDX20 | Low |
| TCGA-DD-AADA-01A-11R-A41C-07 | 1 | 41.1 | 1.835252 | DDX20 | Low |
| TCGA-DD-AAE4-01A-11R-A41C-07 | 1 | 20.26667 | 1.840678 | DDX20 | Low |
| TCGA-DD-A73A-01A-12R-A32O-07 | 1 | 24.26667 | 1.842496 | DDX20 | Low |
| TCGA-HP-A5MZ-01A-21R-A27V-07 | 1 | 3.033333 | 1.842559 | DDX20 | Low |
| TCGA-2Y-A9GW-01A-11R-A38B-07 | 2 | 42.36667 | 1.843917 | DDX20 | Low |
| TCGA-DD-A4NI-01A-11R-A27V-07 | 1 | 27.2 | 1.861462 | DDX20 | Low |
| TCGA-WJ-A86L-01A-12R-A39D-07 | 1 | 11.5 | 1.873321 | DDX20 | Low |
| TCGA-DD-AAE7-01A-11R-A41C-07 | 1 | 21.46667 | 1.880617 | DDX20 | Low |
| TCGA-DD-AAVW-01A-11R-A41C-07 | 1 | 77.23333 | 1.885077 | DDX20 | Low |
| TCGA-DD-AACI-01A-11R-A41C-07 | 1 | 53.93333 | 1.885948 | DDX20 | Low |
| TCGA-EP-A2KC-01A-11R-A213-07 | 1 | 0.633333 | 1.889377 | DDX20 | Low |
| TCGA-DD-A3A8-01A-11R-A22L-07 | 1 | 0.366667 | 1.891978 | DDX20 | Low |
| TCGA-DD-AACE-01A-11R-A41C-07 | 1 | 72.8 | 1.898557 | DDX20 | Low |
| TCGA-KR-A7K2-01A-12R-A33R-07 | 1 | 27.63333 | 1.898811 | DDX20 | Low |
| TCGA-DD-A39V-01A-11R-A213-07 | 2 | 21.43333 | 1.909601 | DDX20 | Low |
| TCGA-G3-A3CH-01A-11R-A22L-07 | 1 | 26 | 1.910645 | DDX20 | Low |
| TCGA-CC-A7IF-01A-11R-A33J-07 | 2 | 21.63333 | 1.911106 | DDX20 | Low |
| TCGA-MI-A75C-01A-11R-A32O-07 | 1 | 9.7 | 1.919003 | DDX20 | Low |
| TCGA-BC-A69H-01A-11R-A311-07 | 1 | 14.8 | 1.925231 | DDX20 | Low |
| TCGA-K7-A5RF-01A-11R-A28V-07 | 1 | 21.03333 | 1.926422 | DDX20 | Low |
| TCGA-BD-A3ER-01A-11R-A213-07 | 1 | 37.16667 | 1.928197 | DDX20 | Low |
| TCGA-DD-AAEI-01A-11R-A41C-07 | 1 | 51.03333 | 1.929191 | DDX20 | Low |
| TCGA-FV-A495-01A-11R-A266-07 | 1 | 0.033333 | 1.931661 | DDX20 | Low |
| TCGA-DD-AACW-01A-11R-A41C-07 | 1 | 47.46667 | 1.93228 | DDX20 | Low |
| TCGA-DD-AAVR-01A-11R-A41C-07 | 1 | 83.76667 | 1.934729 | DDX20 | Low |
| TCGA-DD-AACT-01A-11R-A41C-07 | 1 | 52.06667 | 1.942671 | DDX20 | Low |
| TCGA-ZS-A9CD-01A-11R-A37K-07 | | 46.2 | 1.94313 | DDX20 | Low |
| TCGA-DD-AAEG-01A-11R-A39D-07 | 1 | 23.96667 | 1.950771 | DDX20 | Low |
| TCGA-WX-AA46-01A-11R-A39D-07 | 1 | 25.2 | 1.950981 | DDX20 | Low |
| TCGA-DD-AAE9-01A-11R-A41C-07 | 1 | 24.06667 | 1.953674 | DDX20 | Low |
| TCGA-G3-A7M8-01A-11R-A33R-07 | 1 | 14.33333 | 1.958727 | DDX20 | Low |
| TCGA-G3-AAV5-01A-11R-A37K-07 | 1 | 11.8 | 1.960556 | DDX20 | Low |
| TCGA-FV-A3I0-01A-11R-A22L-07 | 1 | 28.26667 | 1.970203 | DDX20 | Low |
| TCGA-2Y-A9H4-01A-11R-A38B-07 | 1 | 48.4 | 1.998543 | DDX20 | Low |
| TCGA-DD-AAVQ-01A-11R-A41C-07 | 1 | 90.93333 | 2.005929 | DDX20 | Low |
| TCGA-DD-A1ED-01A-11R-A155-07 | 1 | 76.7 | 2.007177 | DDX20 | Low |
| TCGA-5R-AAAM-01A-12R-A41C-07 | 1 | 1.533333 | 2.00738 | DDX20 | Low |
| TCGA-DD-AAD2-01A-11R-A41C-07 | 1 | 21.93333 | 2.014743 | DDX20 | Low |
| TCGA-DD-A115-01A-11R-A131-07 | 2 | 84.73333 | 2.016246 | DDX20 | Low |
| TCGA-MI-A75G-01A-11R-A32O-07 | 1 | 23.26667 | 2.017876 | DDX20 | Low |
| TCGA-DD-A3A4-01A-11R-A22L-07 | 2 | 20.4 | 2.037547 | DDX20 | Low |
| TCGA-DD-AACY-01A-11R-A41C-07 | 1 | 48.33333 | 2.037554 | DDX20 | Low |
| TCGA-RC-A6M6-01A-11R-A32O-07 | 1 | 0.3 | 2.045091 | DDX20 | Low |
| TCGA-MR-A8JO-01A-12R-A36F-07 | 1 | 11 | 2.045342 | DDX20 | Low |
| TCGA-DD-A118-01A-11R-A131-07 | 1 | 114.5667 | 2.04763 | DDX20 | Low |
| TCGA-BD-A2L6-01A-11R-A213-07 | 1 | 45.43333 | 2.049805 | DDX20 | Low |
| TCGA-MR-A520-01A-11R-A266-07 | 1 | 7.633333 | 2.055147 | DDX20 | Low |
| TCGA-CC-A3MB-01A-11R-A213-07 | 2 | 10.5 | 2.057622 | DDX20 | Low |
| TCGA-5C-AAPD-01A-21R-A39D-07 | 1 | 0.666667 | 2.058116 | DDX20 | Low |
| TCGA-O8-A75V-01A-11R-A32O-07 | 1 | 17.93333 | 2.06549 | DDX20 | Low |
| TCGA-DD-A113-01A-11R-A131-07 | 1 | 80.83333 | 2.06875 | DDX20 | Low |
| TCGA-DD-AAEA-01A-11R-A41C-07 | 1 | 19.16667 | 2.070867 | DDX20 | Low |
| TCGA-DD-A116-01A-11R-A131-07 | 1 | 54.06667 | 2.071711 | DDX20 | Low |
| TCGA-BC-A10X-01A-11R-A131-07 | 1 | 25.66667 | 2.07414 | DDX20 | Low |
| TCGA-ED-A7PZ-01A-11R-A33R-07 | 1 | 0.2 | 2.077577 | DDX20 | Low |
| TCGA-BC-A69I-01A-11R-A311-07 | 1 | 12.9 | 2.07806 | DDX20 | Low |
| TCGA-ZS-A9CG-01A-11R-A37K-07 | 1 | 11.36667 | 2.079596 | DDX20 | Low |
| TCGA-DD-AAW1-01A-11R-A41C-07 | 1 | 66.3 | 2.086794 | DDX20 | Low |
| TCGA-MI-A75E-01A-11R-A32O-07 | 1 | 16.9 | 2.090837 | DDX20 | Low |
| TCGA-G3-A7M7-01A-12R-A352-07 | 1 | 12.03333 | 2.097144 | DDX20 | Low |
| TCGA-DD-A4NV-01A-11R-A311-07 | 1 | 79.93333 | 2.098646 | DDX20 | Low |
| TCGA-DD-AAEK-01A-11R-A41C-07 | 1 | 35.56667 | 2.103257 | DDX20 | Low |
| TCGA-CC-A3MC-01A-11R-A22L-07 | 1 | 12.1 | 2.105818 | DDX20 | Low |
| TCGA-2Y-A9H5-01A-11R-A38B-07 | 2 | 18.5 | 2.123046 | DDX20 | Low |
| TCGA-DD-AACA-02B-11R-A41C-07 | 1 | 76.7 | 2.126794 | DDX20 | Low |
| TCGA-XR-A8TC-01A-11R-A36F-07 | 1 | 44.63333 | 2.130032 | DDX20 | Low |
| TCGA-DD-AACG-01A-11R-A41C-07 | 2 | 15.63333 | 2.131201 | DDX20 | Low |
| TCGA-BC-A10T-01A-11R-A131-07 | 2 | 27.9 | 2.131625 | DDX20 | Low |
| TCGA-DD-A1EI-01A-11R-A131-07 | 1 | 6.1 | 2.131986 | DDX20 | Low |
| TCGA-DD-AAD6-01A-11R-A41C-07 | 1 | 22.4 | 2.135104 | DDX20 | Low |
| TCGA-DD-A3A5-01A-11R-A22L-07 | 2 | 104.1667 | 2.135551 | DDX20 | Low |
| TCGA-DD-AACO-01A-11R-A41C-07 | 1 | 62.53333 | 2.137256 | DDX20 | Low |
| TCGA-DD-AACU-01A-11R-A41C-07 | 1 | 52.23333 | 2.137677 | DDX20 | Low |
| TCGA-DD-AAE0-01A-11R-A41C-07 | 1 | 18.5 | 2.139354 | DDX20 | Low |
| TCGA-G3-A25V-01A-11R-A16W-07 | 1 | 28.66667 | 2.147453 | DDX20 | Low |
| TCGA-DD-AAE6-01A-11R-A41C-07 | 1 | 4.7 | 2.15565 | DDX20 | Low |
| TCGA-XR-A8TD-01A-12R-A39D-07 | 1 | 34.33333 | 2.159063 | DDX20 | Low |
| TCGA-DD-AAD3-01A-11R-A41C-07 | 1 | 43.16667 | 2.166046 | DDX20 | Low |
| TCGA-EP-A2KA-01A-11R-A180-07 | 2 | 20.9 | 2.169356 | DDX20 | Low |
| TCGA-DD-A1EH-01A-11R-A131-07 | 1 | 49.83333 | 2.17956 | DDX20 | Low |
| TCGA-RC-A7SK-01A-11R-A352-07 | 1 | 15.73333 | 2.19201 | DDX20 | Low |
| TCGA-RC-A7S9-01A-11R-A33R-07 | 1 | 21.33333 | 2.192238 | DDX20 | Low |
| TCGA-DD-AACQ-01A-11R-A41C-07 | 2 | 14.4 | 2.19242 | DDX20 | Low |
| TCGA-ZP-A9CY-01A-11R-A38B-07 | 1 | 26.06667 | 2.202614 | DDX20 | Low |
| TCGA-BC-A5W4-01A-11R-A28V-07 | 1 | 18.23333 | 2.207802 | DDX20 | Low |
| TCGA-DD-A4NB-01A-12R-A266-07 | 1 | 32.96667 | 2.208422 | DDX20 | Low |
| TCGA-DD-A3A1-01A-11R-A213-07 | 1 | 7.766667 | 2.212991 | DDX20 | Low |
| TCGA-G3-A25X-01A-11R-A16W-07 | 1 | 59.3 | 2.214469 | DDX20 | Low |
| TCGA-GJ-A6C0-01A-12R-A311-07 | | 1.033333 | 2.218037 | DDX20 | Low |
| TCGA-MI-A75H-01A-11R-A32O-07 | 1 | 24.9 | 2.218573 | DDX20 | Low |
| TCGA-G3-A3CG-01A-11R-A213-07 | 1 | 22.43333 | 2.226019 | DDX20 | Low |
| TCGA-ED-A7PY-01A-11R-A33R-07 | 1 | 13 | 2.226853 | DDX20 | Low |
| TCGA-2Y-A9H9-01A-21R-A39D-07 | 1 | 23.23333 | 2.230723 | DDX20 | Low |
| TCGA-WQ-A9G7-01A-11R-A37K-07 | 1 | 1 | 2.23118 | DDX20 | Low |
| TCGA-DD-AAW2-01A-11R-A41C-07 | 1 | 61.83333 | 2.2362 | DDX20 | Low |
| TCGA-DD-AAE2-01A-11R-A41C-07 | 1 | 21.26667 | 2.236835 | DDX20 | Low |
| TCGA-ZP-A9D1-01A-11R-A38B-07 | 1 | 0.7 | 2.254447 | DDX20 | Low |
| TCGA-DD-AACC-01A-11R-A41C-07 | 2 | 56.16667 | 2.255589 | DDX20 | Low |
| TCGA-GJ-A3OU-01A-31R-A38B-07 | 1 | 29.3 | 2.258461 | DDX20 | Low |
| TCGA-UB-A7MF-01A-11R-A33J-07 | 2 | 7.133333 | 2.259452 | DDX20 | Low |
| TCGA-KR-A7K8-01A-11R-A33J-07 | 1 | 30.2 | 2.259821 | DDX20 | Low |
| TCGA-DD-A73F-01A-11R-A32O-07 | 1 | 36.16667 | 2.261821 | DDX20 | Low |
| TCGA-DD-A4NK-01A-11R-A28V-07 | 2 | 40.33333 | 2.269768 | DDX20 | Low |
| TCGA-G3-A25S-01A-11R-A16W-07 | 2 | 13.86667 | 2.270313 | DDX20 | Low |
| TCGA-DD-AADQ-01A-11R-A41C-07 | 1 | 14.53333 | 2.273482 | DDX20 | Low |
| TCGA-DD-AAEH-01A-11R-A41C-07 | 1 | 26.13333 | 2.274453 | DDX20 | Low |
| TCGA-EP-A26S-01A-11R-A16W-07 | 1 | 20.26667 | 2.274795 | DDX20 | Low |
| TCGA-ZP-A9D4-01A-11R-A37K-07 | 1 | 13.16667 | 2.282661 | DDX20 | Low |
| TCGA-UB-A7MD-01A-12R-A352-07 | 1 | 1.733333 | 2.294091 | DDX20 | Low |
| TCGA-G3-A25T-01A-11R-A16W-07 | 1 | 51.76667 | 2.296382 | DDX20 | Low |
| TCGA-T1-A6J8-01A-11R-A32O-07 | 1 | 0.766667 | 2.29783 | DDX20 | Low |
| TCGA-ZS-A9CF-01A-11R-A38B-07 | 1 | 80.4 | 2.29894 | DDX20 | Low |
| TCGA-G3-AAV2-01A-11R-A37K-07 | 1 | 12.4 | 2.308426 | DDX20 | Low |
| TCGA-DD-A4NH-01A-11R-A27V-07 | 1 | 30.56667 | 2.309069 | DDX20 | Low |
| TCGA-FV-A496-01A-11R-A266-07 | 1 | 0.333333 | 2.314741 | DDX20 | Low |
| TCGA-UB-AA0V-01A-11R-A38B-07 | 1 | 10.46667 | 2.319638 | DDX20 | Low |
| TCGA-DD-AADG-01A-11R-A41C-07 | 1 | 38.16667 | 2.320855 | DDX20 | Low |
| TCGA-5R-AA1D-01A-11R-A38B-07 | 1 | 14.96667 | 2.320953 | DDX20 | Low |
| TCGA-BC-A10Q-01A-11R-A131-07 | 2 | 37.83333 | 2.327048 | DDX20 | Low |
| TCGA-ZS-A9CE-01A-11R-A37K-07 | 1 | 41.36667 | 2.337802 | DDX20 | Low |
| TCGA-DD-AAVU-01A-11R-A41C-07 | 1 | 73.4 | 2.343207 | DDX20 | Low |
| TCGA-G3-AAV3-01A-11R-A37K-07 | 1 | 13.73333 | 2.343692 | DDX20 | High |
| TCGA-RC-A6M5-01A-11R-A32O-07 | 1 | 0.5 | 2.346056 | DDX20 | High |
| TCGA-2Y-A9GZ-01A-11R-A39D-07 | 2 | 28.26667 | 2.356382 | DDX20 | High |
| TCGA-CC-A7IE-01A-21R-A38B-07 | 2 | 7.233333 | 2.359105 | DDX20 | High |
| TCGA-KR-A7K0-01A-12R-A33R-07 | 1 | 2.166667 | 2.363144 | DDX20 | High |
| TCGA-DD-AAVX-01A-11R-A41C-07 | 1 | 57.26667 | 2.363683 | DDX20 | High |
| TCGA-ZP-A9D0-01A-11R-A37K-07 | 1 | 36.36667 | 2.366984 | DDX20 | High |
| TCGA-DD-AACN-01A-11R-A41C-07 | 1 | 43.4 | 2.36742 | DDX20 | High |
| TCGA-2Y-A9H8-01A-11R-A39D-07 | 2 | 21.1 | 2.371917 | DDX20 | High |
| TCGA-CC-A9FW-01A-11R-A37K-07 | 1 | 8.266667 | 2.380384 | DDX20 | High |
| TCGA-DD-A1EG-01A-11R-A213-07 | 2 | 45.73333 | 2.380771 | DDX20 | High |
| TCGA-CC-A9FS-01A-11R-A37K-07 | 1 | 7.033333 | 2.381989 | DDX20 | High |
| TCGA-DD-AACF-01A-11R-A41C-07 | 2 | 12.16667 | 2.387686 | DDX20 | High |
| TCGA-DD-AAED-01A-12R-A41C-07 | 1 | 25.43333 | 2.394349 | DDX20 | High |
| TCGA-HP-A5N0-01A-11R-A28V-07 | 2 | 38.23333 | 2.396885 | DDX20 | High |
| TCGA-RC-A6M4-01A-11R-A32O-07 | 1 | 0.733333 | 2.398085 | DDX20 | High |
| TCGA-ED-A627-01A-12R-A311-07 | 1 | 14.1 | 2.400102 | DDX20 | High |
| TCGA-ED-A4XI-01A-11R-A266-07 | 1 | 27.3 | 2.402532 | DDX20 | High |
| TCGA-BC-A10Z-01A-11R-A131-07 | 1 | 1.133333 | 2.413489 | DDX20 | High |
| TCGA-DD-A73B-01A-12R-A32O-07 | 2 | 9.433333 | 2.418765 | DDX20 | High |
| TCGA-RG-A7D4-01A-12R-A33R-07 | 1 | 36.6 | 2.420902 | DDX20 | High |
| TCGA-CC-A5UC-01A-11R-A28V-07 | 2 | 11.56667 | 2.424085 | DDX20 | High |
| TCGA-3K-AAZ8-01A-12R-A39D-07 | 1 | 13.2 | 2.425035 | DDX20 | High |
| TCGA-G3-A5SI-01A-31R-A27V-07 | 2 | 25.6 | 2.427761 | DDX20 | High |
| TCGA-DD-A4NQ-01A-21R-A28V-07 | 2 | 12.43333 | 2.429005 | DDX20 | High |
| TCGA-DD-AACJ-01A-11R-A41C-07 | 1 | 70.06667 | 2.439551 | DDX20 | High |
| TCGA-ED-A7XO-01A-11R-A352-07 | 1 | 14.23333 | 2.440547 | DDX20 | High |
| TCGA-2Y-A9GV-01A-11R-A38B-07 | 2 | 84.4 | 2.444635 | DDX20 | High |
| TCGA-PD-A5DF-01A-11R-A27V-07 | 2 | 21.3 | 2.446993 | DDX20 | High |
| TCGA-DD-AAVP-01A-11R-A41C-07 | 1 | 91.73333 | 2.455857 | DDX20 | High |
| TCGA-DD-A1EB-01A-11R-A131-07 | 1 | 67.23333 | 2.457076 | DDX20 | High |
| TCGA-FV-A2QR-01A-11R-A213-07 | 2 | 19.36667 | 2.465747 | DDX20 | High |
| TCGA-ED-A66Y-01A-11R-A311-07 | 1 | 9.866667 | 2.467973 | DDX20 | High |
| TCGA-FV-A2QQ-01A-11R-A22L-07 | 1 | 24.3 | 2.469606 | DDX20 | High |
| TCGA-2Y-A9H0-01A-11R-A38B-07 | 1 | 122.5 | 2.473852 | DDX20 | High |
| TCGA-ED-A8O6-01A-11R-A36F-07 | 1 | 1.866667 | 2.475012 | DDX20 | High |
| TCGA-DD-AADR-01A-11R-A41C-07 | 1 | 67.6 | 2.480745 | DDX20 | High |
| TCGA-K7-A5RG-01A-11R-A28V-07 | 1 | 17.3 | 2.483274 | DDX20 | High |
| TCGA-G3-A25Y-01A-11R-A16W-07 | 2 | 15.06667 | 2.484703 | DDX20 | High |
| TCGA-LG-A9QC-01A-11R-A37K-07 | 1 | 14.16667 | 2.488079 | DDX20 | High |
| TCGA-DD-A4NO-01A-11R-A28V-07 | 1 | 74.83333 | 2.489691 | DDX20 | High |
| TCGA-CC-A7IH-01A-11R-A33J-07 | 1 | 12.16667 | 2.490134 | DDX20 | High |
| TCGA-DD-A4NN-01A-11R-A28V-07 | 2 | 29.96667 | 2.490272 | DDX20 | High |
| TCGA-DD-AADW-01A-11R-A39D-07 | 1 | 19.56667 | 2.490974 | DDX20 | High |
| TCGA-DD-A73C-01A-12R-A33J-07 | 1 | 23.36667 | 2.492981 | DDX20 | High |
| TCGA-BW-A5NO-01A-11R-A27V-07 | 1 | 0.666667 | 2.499432 | DDX20 | High |
| TCGA-WX-AA44-01A-11R-A39D-07 | 1 | 20.5 | 2.50203 | DDX20 | High |
| TCGA-2Y-A9H7-01A-11R-A39D-07 | 1 | 38.93333 | 2.504166 | DDX20 | High |
| TCGA-DD-AACS-01A-11R-A41C-07 | 1 | 60.13333 | 2.507579 | DDX20 | High |
| TCGA-CC-A7IL-01A-11R-A33R-07 | 2 | 9.266667 | 2.510761 | DDX20 | High |
| TCGA-ED-A459-01A-11R-A266-07 | 1 | 30.33333 | 2.51488 | DDX20 | High |
| TCGA-DD-AADL-01A-11R-A41C-07 | 1 | 21.2 | 2.517319 | DDX20 | High |
| TCGA-2Y-A9HB-01A-11R-A39D-07 | 1 | 8.666667 | 2.518404 | DDX20 | High |
| TCGA-2V-A95S-01A-11R-A37K-07 | 1 |  | 2.523029 | DDX20 | High |
| TCGA-DD-A73D-01A-12R-A32O-07 | 1 | 23.1 | 2.525272 | DDX20 | High |
| TCGA-DD-AAD5-01A-11R-A41C-07 | 1 | 44.83333 | 2.527005 | DDX20 | High |
| TCGA-DD-AAEE-01A-11R-A41C-07 | 1 | 27 | 2.538728 | DDX20 | High |
| TCGA-DD-AAVY-01A-11R-A41C-07 | 1 | 65.66667 | 2.540414 | DDX20 | High |
| TCGA-DD-A4ND-01A-11R-A266-07 | 1 | 91.53333 | 2.550277 | DDX20 | High |
| TCGA-2Y-A9GX-01A-11R-A38B-07 | 1 | 81.4 | 2.55572 | DDX20 | High |
| TCGA-DD-A4NF-01A-11R-A27V-07 | 1 | 31.4 | 2.556398 | DDX20 | High |
| TCGA-ED-A7XP-01A-11R-A352-07 | 1 | 13.33333 | 2.570773 | DDX20 | High |
| TCGA-UB-A7ME-01A-11R-A33J-07 | 1 | 16.2 | 2.571948 | DDX20 | High |
| TCGA-DD-AACX-01A-11R-A41C-07 | 1 | 5.666667 | 2.572475 | DDX20 | High |
| TCGA-DD-AAVV-01A-11R-A41C-07 | 1 | 81.83333 | 2.584694 | DDX20 | High |
| TCGA-MI-A75I-01A-11R-A32O-07 | 1 | 21 | 2.586244 | DDX20 | High |
| TCGA-FV-A3R2-01A-11R-A22L-07 | 1 | 6.466667 | 2.588099 | DDX20 | High |
| TCGA-5C-A9VG-01A-11R-A37K-07 | 1 | 10.93333 | 2.5903 | DDX20 | High |
| TCGA-G3-A5SL-01A-11R-A27V-07 | 1 | 20.7 | 2.605049 | DDX20 | High |
| TCGA-BC-4072-01B-11R-A155-07 | 2 | 49.66667 | 2.610121 | DDX20 | High |
| TCGA-G3-AAV1-01A-11R-A38B-07 | 2 | 11.96667 | 2.610308 | DDX20 | High |
| TCGA-CC-A7IG-01A-11R-A33J-07 | 2 | 9.966667 | 2.610682 | DDX20 | High |
| TCGA-ED-A66X-01A-11R-A311-07 | 1 | 13.53333 | 2.612797 | DDX20 | High |
| TCGA-DD-AAVS-01A-11R-A41C-07 | 1 | 60.76667 | 2.614891 | DDX20 | High |
| TCGA-DD-AACA-01A-11R-A41C-07 | 1 | 76.7 | 2.630971 | DDX20 | High |
| TCGA-ED-A7PX-01A-51R-A352-07 | 1 | 0.2 | 2.632672 | DDX20 | High |
| TCGA-DD-A1EA-01A-11R-A131-07 | 1 | 80.5 | 2.644678 | DDX20 | High |
| TCGA-FV-A3I1-01A-11R-A22L-07 | 1 | 8.233333 | 2.644725 | DDX20 | High |
| TCGA-BD-A3EP-01A-11R-A22L-07 | 1 | 13.63333 | 2.661333 | DDX20 | High |
| TCGA-UB-A7MC-01A-11R-A33R-07 | 1 | 16.66667 | 2.67892 | DDX20 | High |
| TCGA-DD-A1EL-01A-11R-A155-07 | 2 | 13.83333 | 2.683637 | DDX20 | High |
| TCGA-EP-A3RK-01A-11R-A22L-07 | 1 | 12.1 | 2.685648 | DDX20 | High |
| TCGA-DD-A1EC-01A-21R-A131-07 | 1 | 20.06667 | 2.688005 | DDX20 | High |
| TCGA-CC-A9FV-01A-11R-A37K-07 | 1 | 0 | 2.697577 | DDX20 | High |
| TCGA-BW-A5NP-01A-11R-A27V-07 | 1 | 0 | 2.700282 | DDX20 | High |
| TCGA-CC-5262-01A-01R-A131-07 | 1 | 3.433333 | 2.708128 | DDX20 | High |
| TCGA-DD-AAD8-01A-11R-A41C-07 | 1 | 40.63333 | 2.711416 | DDX20 | High |
| TCGA-DD-A4NR-01A-11R-A311-07 | 1 | 0.3 | 2.711762 | DDX20 | High |
| TCGA-DD-AACA-02A-11R-A41C-07 | 1 | 76.7 | 2.717138 | DDX20 | High |
| TCGA-DD-AAD1-01A-11R-A41C-07 | 1 | 18.8 | 2.720857 | DDX20 | High |
| TCGA-DD-A39Y-01A-11R-A213-07 | 1 | 5.7 | 2.723418 | DDX20 | High |
| TCGA-DD-AACZ-01A-11R-A41C-07 | 1 | 5.7 | 2.727084 | DDX20 | High |
| TCGA-DD-A11A-01A-11R-A131-07 | 1 | 2.633333 | 2.731149 | DDX20 | High |
| TCGA-BC-A3KG-01A-11R-A213-07 | 1 | 22.66667 | 2.732546 | DDX20 | High |
| TCGA-ED-A97K-01A-21R-A38B-07 | 1 | 0.2 | 2.736869 | DDX20 | High |
| TCGA-DD-AADV-01A-11R-A39D-07 | 1 | 19.13333 | 2.739654 | DDX20 | High |
| TCGA-XR-A8TG-01A-11R-A36F-07 | 1 | 29.93333 | 2.7496 | DDX20 | High |
| TCGA-G3-A5SJ-01A-11R-A27V-07 | 1 | 23.26667 | 2.755039 | DDX20 | High |
| TCGA-CC-5258-01A-01R-A131-07 | 1 | 4.3 | 2.755794 | DDX20 | High |
| TCGA-DD-A39W-01A-11R-A213-07 | | 27.56667 | 2.756174 | DDX20 | High |
| TCGA-2Y-A9GY-01A-11R-A38B-07 | 2 | 25.23333 | 2.759647 | DDX20 | High |
| TCGA-BC-A10U-01A-11R-A131-07 | 2 | 27.9 | 2.761226 | DDX20 | High |
| TCGA-CC-A8HV-01A-11R-A36F-07 | 2 | 9.3 | 2.765863 | DDX20 | High |
| TCGA-DD-AAW3-01A-11R-A41C-07 | 1 | 54.43333 | 2.76688 | DDX20 | High |
| TCGA-GJ-A9DB-01A-11R-A37K-07 | | 2.233333 | 2.768693 | DDX20 | High |
| TCGA-FV-A23B-01A-11R-A16W-07 | 2 | 61.73333 | 2.769172 | DDX20 | High |
| TCGA-G3-A25U-01A-11R-A16W-07 | 1 | 54.53333 | 2.772088 | DDX20 | High |
| TCGA-DD-AAW0-01A-11R-A41C-07 | 1 | 67.16667 | 2.778469 | DDX20 | High |
| TCGA-DD-AADP-01A-11R-A39D-07 | 1 | 15.26667 | 2.784592 | DDX20 | High |
| TCGA-DD-AADI-01A-11R-A41C-07 | 1 | 36.16667 | 2.793264 | DDX20 | High |
| TCGA-G3-A3CK-01A-11R-A213-07 | 1 | 19.5 | 2.801303 | DDX20 | High |
| TCGA-DD-AAC8-01A-11R-A41C-07 | 1 | 0.533333 | 2.817715 | DDX20 | High |
| TCGA-DD-AAD0-01A-11R-A41C-07 | 1 | 4.566667 | 2.836493 | DDX20 | High |
| TCGA-K7-AAU7-01A-11R-A38B-07 | 1 | 11.96667 | 2.837327 | DDX20 | High |
| TCGA-BC-A10Y-01A-11R-A131-07 | 2 | 23.7 | 2.841011 | DDX20 | High |
| TCGA-DD-A4NS-01A-11R-A311-07 | 2 | 81.86667 | 2.84681 | DDX20 | High |
| TCGA-DD-A4NJ-01A-11R-A27V-07 | 1 | 30.93333 | 2.854416 | DDX20 | High |
| TCGA-G3-A6UC-01A-21R-A33J-07 | 1 | 22.36667 | 2.856246 | DDX20 | High |
| TCGA-4R-AA8I-01A-11R-A38B-07 | 2 | 8.733333 | 2.864109 | DDX20 | High |
| TCGA-DD-AACL-01A-11R-A41C-07 | | 3.566667 | 2.870344 | DDX20 | High |
| TCGA-DD-AADY-01A-11R-A41C-07 | 1 | 18.5 | 2.87112 | DDX20 | High |
| TCGA-G3-AAV4-01A-11R-A38B-07 | 1 | 0.9 | 2.87113 | DDX20 | High |
| TCGA-BC-A217-01A-11R-A155-07 | 1 | 46.56667 | 2.872522 | DDX20 | High |
| TCGA-ZS-A9CF-02A-11R-A38B-07 | 1 | 80.4 | 2.873139 | DDX20 | High |
| TCGA-CC-A8HU-01A-11R-A36F-07 | 2 | 11.46667 | 2.874058 | DDX20 | High |
| TCGA-XR-A8TE-01A-11R-A36F-07 | 1 | 30.83333 | 2.878668 | DDX20 | High |
| TCGA-XR-A8TF-01A-11R-A36F-07 | 1 | 23.1 | 2.897878 | DDX20 | High |
| TCGA-DD-A73E-01A-12R-A32O-07 | 1 | 1.466667 | 2.923929 | DDX20 | High |
| TCGA-BC-A3KF-01A-11R-A213-07 | 1 | 0.266667 | 2.924235 | DDX20 | High |
| TCGA-EP-A12J-01A-11R-A131-07 | 1 | 19 | 2.946757 | DDX20 | High |
| TCGA-DD-AACB-01A-11R-A41C-07 | 1 | 77.46667 | 2.94854 | DDX20 | High |
| TCGA-ZP-A9CV-01A-11R-A38B-07 | 2 | 36.26667 | 2.95214 | DDX20 | High |
| TCGA-2Y-A9HA-01A-11R-A39D-07 | 2 | 1.2 | 2.964942 | DDX20 | High |
| TCGA-UB-A7MA-01A-11R-A33R-07 | 1 | 28.26667 | 2.968877 | DDX20 | High |
| TCGA-DD-A1EF-01A-11R-A131-07 | 2 | 13.13333 | 2.969285 | DDX20 | High |
| TCGA-DD-A4NA-01A-11R-A266-07 | 1 | 33.6 | 2.992686 | DDX20 | High |
| TCGA-ZP-A9CZ-01A-11R-A38B-07 | 1 | 23.53333 | 2.999495 | DDX20 | High |
| TCGA-CC-A8HT-01A-11R-A36F-07 | 2 | 4.666667 | 3.002561 | DDX20 | High |
| TCGA-BC-A216-01A-11R-A155-07 | 1 | 45.03333 | 3.011021 | DDX20 | High |
| TCGA-UB-AA0U-01A-11R-A38B-07 | 1 | 10.9 | 3.026191 | DDX20 | High |
| TCGA-RC-A6M3-01A-11R-A32O-07 | 1 | 0 | 3.030162 | DDX20 | High |
| TCGA-DD-AACK-01A-11R-A41C-07 | 1 | 0.3 | 3.037432 | DDX20 | High |
| TCGA-LG-A6GG-01A-11R-A311-07 | 1 | 12.9 | 3.042882 | DDX20 | High |
| TCGA-DD-AACV-01A-11R-A41C-07 | 1 | 51.03333 | 3.053668 | DDX20 | High |
| TCGA-QA-A7B7-01A-11R-A32O-07 | 1 | 3.133333 | 3.067709 | DDX20 | High |
| TCGA-YA-A8S7-01A-11R-A37K-07 | 2 | 13.73333 | 3.072794 | DDX20 | High |
| TCGA-DD-AADB-01A-11R-A41C-07 | 1 | 41.4 | 3.106071 | DDX20 | High |
| TCGA-CC-A5UD-01A-11R-A28V-07 | 2 | 10.13333 | 3.10762 | DDX20 | High |
| TCGA-CC-5264-01A-01R-A131-07 | 1 | 3.4 | 3.119243 | DDX20 | High |
| TCGA-CC-A3MA-01A-11R-A213-07 | 2 | 10.1 | 3.147421 | DDX20 | High |
| TCGA-CC-A3M9-01A-11R-A213-07 | 2 | 10 | 3.169475 | DDX20 | High |
| TCGA-2Y-A9GS-01A-12R-A38B-07 | 2 | 24.13333 | 3.185269 | DDX20 | High |
| TCGA-DD-AA3A-01A-11R-A37K-07 | 1 | 13.66667 | 3.19443 | DDX20 | High |
| TCGA-DD-A1EE-01A-11R-A131-07 | 2 | 11.63333 | 3.198856 | DDX20 | High |
| TCGA-DD-AADC-01A-11R-A41C-07 | 2 | 14.16667 | 3.201003 | DDX20 | High |
| TCGA-DD-AACP-01A-11R-A41C-07 | 1 | 13.83333 | 3.205938 | DDX20 | High |
| TCGA-BC-4073-01B-02R-A131-07 | 1 | 28.3 | 3.207303 | DDX20 | High |
| TCGA-DD-A1EJ-01A-11R-A155-07 | 2 | 33.5 | 3.230437 | DDX20 | High |
| TCGA-FV-A4ZQ-01A-11R-A266-07 | 1 | 0.4 | 3.232355 | DDX20 | High |
| TCGA-CC-A9FU-01A-11R-A37K-07 | 1 | 0 | 3.232788 | DDX20 | High |
| TCGA-ED-A82E-01A-11R-A352-07 | 1 | 13.6 | 3.266992 | DDX20 | High |
| TCGA-DD-AACH-01A-11R-A41C-07 | 1 | 6.5 | 3.268199 | DDX20 | High |
| TCGA-CC-5263-01A-01R-A131-07 | 1 | 4.3 | 3.309798 | DDX20 | High |
| TCGA-5R-AA1C-01A-11R-A41C-07 | 1 | 17.33333 | 3.318353 | DDX20 | High |
| TCGA-CC-5261-01A-01R-A131-07 | 1 | 3.233333 | 3.319288 | DDX20 | High |
| TCGA-CC-5260-01A-01R-A131-07 | 1 | 2.9 | 3.333274 | DDX20 | High |
| TCGA-DD-AAVZ-01A-11R-A41C-07 | 1 | 63.33333 | 3.333511 | DDX20 | High |
| TCGA-CC-A5UE-01A-11R-A28V-07 | 2 | 9.066667 | 3.394074 | DDX20 | High |
| TCGA-G3-AAV7-01A-11R-A38B-07 | 1 | 12.03333 | 3.394276 | DDX20 | High |
| TCGA-UB-A7MB-01A-11R-A33R-07 | 1 | 20.03333 | 3.397671 | DDX20 | High |
| TCGA-ED-A5KG-01A-11R-A27V-07 | 1 | 28.46667 | 3.412751 | DDX20 | High |
| TCGA-CC-A7IK-01A-12R-A33R-07 | 2 | 8.733333 | 3.455679 | DDX20 | High |
| TCGA-ED-A8O5-01A-11R-A36F-07 | 1 | 13.53333 | 3.466182 | DDX20 | High |
| TCGA-DD-AADD-01A-11R-A41C-07 | 1 | 41.03333 | 3.475616 | DDX20 | High |
| TCGA-BC-A8YO-01A-11R-A37K-07 | 1 | 18.73333 | 3.50942 | DDX20 | High |
| TCGA-2Y-A9H2-01A-12R-A38B-07 | 1 | 57.7 | 3.528609 | DDX20 | High |
| TCGA-DD-A114-01A-11R-A131-07 | 1 | 38.3 | 3.548504 | DDX20 | High |
| TCGA-CC-A7IJ-01A-11R-A33R-07 | 1 | 12.73333 | 3.562011 | DDX20 | High |
| TCGA-CC-A8HS-01A-11R-A36F-07 | 2 | 10 | 3.566582 | DDX20 | High |
| TCGA-CC-A1HT-01A-11R-A131-07 | 1 | 3.366667 | 3.60334 | DDX20 | High |
| TCGA-CC-A7II-01A-11R-A33J-07 | 1 | 13.3 | 3.62753 | DDX20 | High |
| TCGA-BC-A112-01A-11R-A131-07 | 1 | 5.1 | 3.640174 | DDX20 | High |
| TCGA-ZP-A9D2-01A-11R-A38B-07 | 2 | 25.5 | 3.658386 | DDX20 | High |
| TCGA-BW-A5NQ-01A-11R-A27V-07 | 1 | 0 | 3.668985 | DDX20 | High |
| TCGA-G3-A7M9-01A-23R-A352-07 | | 1.866667 | 4.002369 | DDX20 | High |
| TCGA-G3-A7M6-01A-11R-A33R-07 | 1 | 21.06667 | 4.029581 | DDX20 | High |
| TCGA-BC-A10W-01A-11R-A131-07 | 2 | 3.033333 | 4.942232 | DDX20 | High |
